# Curvature induction and membrane remodeling by FAM134B reticulon homology domain assist selective ER-phagy

**DOI:** 10.1038/s41467-019-10345-3

**Published:** 2019-05-30

**Authors:** Ramachandra M. Bhaskara, Paolo Grumati, Javier Garcia-Pardo, Sissy Kalayil, Adriana Covarrubias-Pinto, Wenbo Chen, Mikhail Kudryashev, Ivan Dikic, Gerhard Hummer

**Affiliations:** 1 Department of Theoretical Biophysics, Max Planck Institute of Biophysics, Max-von-Laue Straße 3, 60438 Frankfurt am Main, Germany; 2 Institute of Biochemistry II, School of Medicine, Goethe University Frankfurt, Theoder-Stern-Kai 7, 60590 Frankfurt am Main, Germany; 3 Buchmann Institute of Molecular Life Sciences, Goethe University Frankfurt, Max-von-Laue Straße 15, 60438 Frankfurt am Main, Germany; 4 Fraunhofer Institute for Molecular Biology and Applied Ecology, Division for Translational Medicine and Pharmacology, Theodor-Stern-Kai 7, 60596 Frankfurt am Main, Germany; 5 Max Planck Institute of Biophysics, Max-von-Laue Straße 3, 60438 Frankfurt am Main, Germany; 6 Institute for Biophysics, Goethe University Frankfurt, 60438 Frankfurt am Main, Germany

**Keywords:** Molecular modelling, Autophagy

## Abstract

FAM134B/RETREG1 is a selective ER-phagy receptor that regulates the size and shape of the endoplasmic reticulum. The structure of its reticulon-homology domain (RHD), an element shared with other ER-shaping proteins, and the mechanism of membrane shaping remain poorly understood. Using molecular modeling and molecular dynamics (MD) simulations, we assemble a structural model for the RHD of FAM134B. Through MD simulations of FAM134B in flat and curved membranes, we relate the dynamic RHD structure with its two wedge-shaped transmembrane helical hairpins and two amphipathic helices to FAM134B functions in membrane-curvature induction and curvature-mediated protein sorting. FAM134B clustering, as expected to occur in autophagic puncta, amplifies the membrane-shaping effects. Electron microscopy of in vitro liposome remodeling experiments support the membrane remodeling functions of the different RHD structural elements. Disruption of the RHD structure affects selective autophagy flux and leads to disease states.

## Abstract (web summary)

FAM134B/RETREG1 is a selective ER-phagy receptor that regulates the size and shape of the endoplasmic reticulum. Here authors use molecular modeling and molecular dynamics simulations to assemble a structural model for the reticulon-homology domain of FAM134B.

## Introduction

The endoplasmic reticulum (ER) forms a large inter-connected membrane system that occupies a substantial fraction of the cell volume. The ER is the major site of protein production, plays a central role in Ca^2+^ homeostasis, and is involved in lipid synthesis. It acts as the central communication and transport hub for several intersecting cellular pathways^1^. The reticulated structure of the ER, composed of flat sheet-like and highly branched tubular networks and matrices, is organized into spatial sub-domains serving its wide variety of functions^2,3^. As a consequence, the ER size, shape, and structure are under constant spatial and temporal regulation.

The ER structure is dynamic and extensively remodeled in response to cellular stress conditions by ER-stress-activated autophagy or by selective ER-phagy^4,5^. Both pathways can also be triggered by unfolded protein response and act to restore metabolic homeostasis^6^. The more selective ER-phagy leads to ER turnover by recruiting the autophagy machinery to specific locations of the ER^5^. As a consequence, major ER components are recycled through autophagy, including lipids and misfolded proteins.

Five ER-resident proteins have been shown to function as receptors for selective ER-phagy: FAM134B^7^, SEC62^8^, RTN3^9^, CCPG1^10^, and ATL3^11^. These integral membrane proteins appear to partition into different sub-domains of the ER. All of them harbor a sequential peptide motif within the cytoplasmic region, enabling binding to LC3/GABARAP proteins associated with phagophore membrane. This specific interaction is mediated by the receptor containing LC3-interacting region (LIR) and recruits the autophagy machinery to structurally diverse ER regions^12^.

FAM134B, the first ER-phagy receptor to be identified, contains a reticulon-homology domain (RHD). FAM134B targets ER sheets for degradation by specific binding to MAP1LC3B, which is associated with the phagophore membrane via its C-terminal LIR. FAM134B is presumed to induce high-membrane curvature in cells, thereby coalescing ER membrane locally into small vesicles, which are engulfed by the growing phagophore and subsequently degraded in the autolysosomes. Cells without FAM134B display massive expansion of the ER sheets^7^.

The biological functions of FAM134B in both normal and disease states, are attributed primarily to its housekeeping function, i.e., ER maintenance via selective autophagy. Loss of function of FAM134B is associated with several diseases and disorders. Defective membrane shaping proteins, especially proteins with RHDs, are often associated with aberrant axonal development and neurodegenerative disorders^13^. In humans, genetic variants resulting in loss of FAM134B function cause severe sensory neuropathy (HSANII)^14^. FAM134B is also implicated in the suppression of viral replication during Ebola, Dengue, Zika, and West Nile viral infections^15,16^. Non-structural viral proteases such as NS2S3 specifically cleave FAM134B, thereby subverting ER-phagy^15^. Specific mutations in FAM134B and expression profiles are strongly correlated with colorectal cancers^17^. Increased expression of FAM134B is also implicated with susceptibility to vascular dementia and allergic rhinitis^17,18^.

Discerning the functions associated with the RHDs in membranes is severely restricted by the lack of 3D structure. Limited characterization so far reveals that the structural elements of the RHD and its membrane topology are common to membrane-shaping proteins (canonical RTNs, REEPS, and DP1/YOP-like)^19–22^ and selective ER-phagy receptors (FAM134B, ATG40, RTN3)^5^. FAM134B offers a unique system to study the role of RHDs. At the molecular level, the presence of both a RHD and a LIR motif within the same protein couples local curvature induction and sensing functions to organelle remodeling and maintenance associated with ER-phagy. However, the mechanistic basis of FAM134B-mediated ER-phagy remains unresolved.

Here, we use computer modeling to build a structural model of the RHD of FAM134B and to characterize its ability to sense and induce membrane curvature. With electron microscopy imaging of the membrane remodeling activities of different deletion constructs, and with in cell immunofluorescence (IF) studies of ER-phagy, we test the functional predictions of the model. We identify the mechanism of curvature induction by FAM134B and shed light on its role in selective ER-phagy. The unique domain structure of the RHD emerges as the central factor in the induction of highly curved vesicles by FAM134B in vitro. Our model also provides a molecular explanation for the loss of function associated with genetic variants and proteolytic cleavage products of FAM134B.

## Results

### Structural model for FAM134B-RHD

**Fig. 1:**
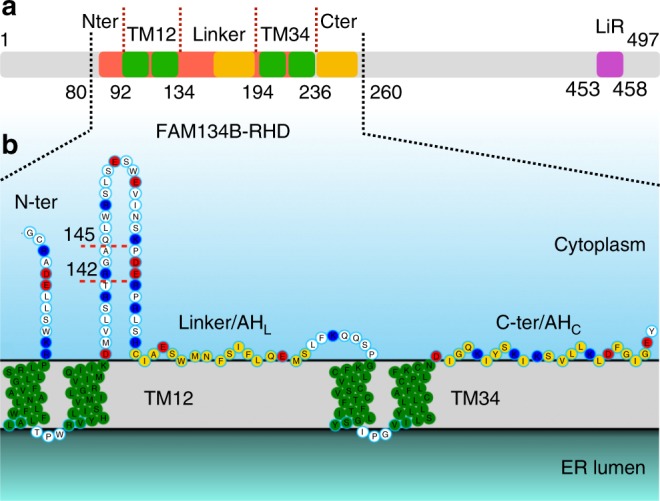
Sequence and topology of FAM134B. **a** Schematic of the full length FAM134B sequence. The RHD consists of two transmembrane segments (green, TM12 and TM34) separated by a 60-residue linker, and two additional terminal segments. The C-terminal fragment of the RHD and the linker-helix (yellow) form conserved amphipathic helices. **b** Topology of FAM134B-RHD (80–260). Charged residues (K/R blue and D/E red), TM segments (green) and amphipathic helices (yellow) are highlighted. Genetic variant Q145X and proteolytic cleavage product R142X result in truncated proteins (red dotted line) disrupting the RHD. The N-terminal and C-terminal disordered regions (not modeled, gray) flank the RHD on the cytosolic face of the ER membrane

The FAM134B sequence contains a RHD (residues 83–235; Fig. 1). The RHD resembles canonical reticulon proteins and displays a similar hydrophobicity profile indicating a likely evolutionary relationship (PF02453, bit score = 40.54; *E*-value = 0.00049; Supplementary Fig. 1; Supplementary Note 1). Using a wide range of sequence-based analysis and assignment procedures, we concluded that the RHD forms a membrane embedded, structured region of FAM134B (Supplementary Fig. 2). The RHD of FAM134B is characterized by two large transmembrane (TM) segments separated by a 60-residue long linker segment. C-terminal to the second TM segment is a conserved amphipathic helix (Fig. 1). The RHD is flanked by a variable N-terminal disordered fragment (1–80) and a C-terminal (260–497) disordered fragment containing the conserved functional LIR (Fig. 1; Supplementary Fig. 2).

**Fig. 2:**
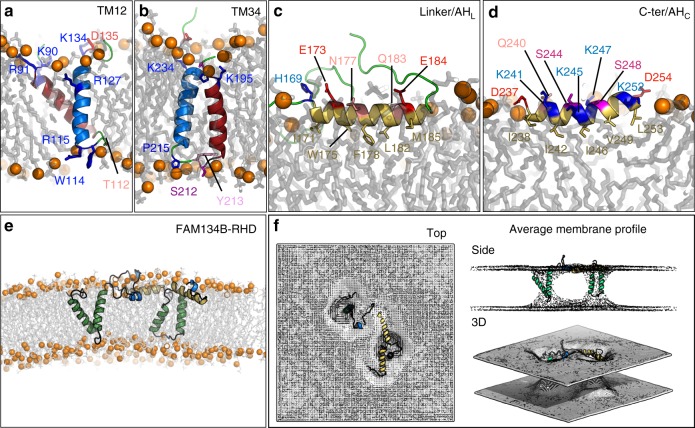
3D structural model of FAM134B-RHD from MD simulations. **a**, **b** Transmembrane fragments fold into helical hairpins (red and blue helices in (**a**) TM12 and (**b**) TM34. Flanking charged and polar residues and luminal loop residues anchor the two hairpins within the ER membrane (labeled side chains). **c**, **d** Linker and C-terminal fragments form amphipathic helices (**c**) AH_L_ and (**d**) AH_C_ (yellow cartoon). Polar (colored labels) and apolar residues (yellow labels) on opposite sides position the helices at the water–bilayer interface. **e** Overlapping, individually refined fragment structures were used to assemble the FAM134B-RHD (80–260) structural model. The model was first equilibrated using coarse-grained simulations and then refined with all-atom MD simulations. **f** Time-averaged local membrane profile (gray mesh; top, side, and 3D views) around FAM134B-RHD (colored) computed from all-atom MD simulations displays perturbations of the bilayer structure

We built a molecular model of FAM134B-RHD by integrating fragment-based modeling with extensive molecular dynamic (MD) simulations (see the section “Methods”; Fig. 2). Rough initial models of the two TM helical hairpins and the two amphipathic helices were constructed from solved structures with similar sequence and matching secondary structure (Supplementary Table 1). These initial fragment models were then subjected to extensive conformational sampling, first, using coarse-grained (CG) simulations and then by all-atom MD simulations (Supplementary Figs. 3–7; Supplementary Table 2; Supplementary Notes 2–4). Finally, the refined fragment structures with appropriate membrane positions and orientations were assembled into a structural model of FAM134B-RHD (Supplementary Table 3; Supplementary Note 5).

TM segments TM12 and TM34 of the RHD fold into TM hairpins (Fig. 2a, b; Supplementary Note 2). The short TM helices connected by a Gly/Pro-rich polar loop (3–4 residues) display conformational characteristics of helical hairpins (Supplementary Figs. 3–5). Specific pair-wise interactions across the two helices stabilize their helical hairpin structure (Supplementary Figs. 4 and 5). Charged and polar residues within their luminal loops and ends anchor the hairpins firmly into both leaflets of the ER membrane. The short hydrophobic helices (5–6 turns each), their helix–helix crossing angle (≈50–60°) and a slight tilt (≈10–15°) of the individual hairpins establish hydrophobic mismatch in PC-rich bilayers (Fig. 2a, b).

In addition to the two TM helical hairpins, the FAM134B-RHD contains two cytoplasmic amphipathic helices (AH_L_ and AH_C_; Fig. 2c, d) with predicted large hydrophobic moments (Supplementary Figs. 6 and 7; Supplementary Notes 3, 4). In CG simulations of the helical fragments AH_L_ and AH_C_, we observed membrane docking and embedding events in the presence of lipid bilayers, illustrating their amphipathic nature (Supplementary Figs. 6 and 7). The docked orientation of these fragments was consistent with the predicted hydrophobic moments $\left(\langle \mu H\rangle _{\mathrm{AH}_{\mathrm{L}}} = 0.35\;{\mathrm{and}}\;\langle \mu H\rangle _{\mathrm{AH}_{\mathrm{C}}} = 0.48 \right)$. In all-atom MD simulations of the membrane-docked fragments of AH_L_ and AH_C_, the amphipathic segments remained in helical conformations (Fig. 2c, d). By contrast, in all-atom simulations of the fragments in aqueous solution, disordered and unfolded conformations accumulated, indicating the importance of membrane-interactions for the folding of amphipathic helices (Supplementary Figs. 6 and 7).

The RHD of FAM134B is thus assembled from four major fragments (Fig. 2e; Supplementary Note 5). Two TM hairpin structures firmly anchor the protein into both leaflets of the ER membrane. A flexible cytoplasmic linker bridges the two TM hairpins. The two amphipathic helices interact strongly with the cytoplasmic leaflet and flank the TM34 segment on both sides. Structural features of these fragments, including their membrane interactions, are well preserved in both CG and all-atom MD simulations (Supplementary Table 3).

### FAM134B-RHD induces membrane curvature

FAM134B-RHD perturbs the local bilayer structure and breaks the bilayer symmetry. We quantified its effect on local membrane shape and structure by mapping the average membrane thickness profile around the RHD from all-atom MD simulations (Fig. 2f). The RHD formed a large asymmetric membrane inclusion in the ER membrane. Near the amphipathic helices, the area per lipid increased by stretching the cytoplasmic leaflet. We also observed a reduced bilayer thickness in the vicinity of the TM hairpins. The hydrophobic mismatch of the TM hairpins locally compresses the membrane (Supplementary Fig. 8).

We reasoned that local bilayer asymmetric stretching and compression by FAM134B-RHD could deform the natural bilayer shape and result in curved membrane structures. However, the inherent restriction to MD simulations under periodic boundary conditions prohibits the development of long wavelength shape fluctuations in the bilayer. To overcome this limitation and study possible large-scale membrane shape changes induced by FAM134B-RHD, we designed an in silico curvature assay using open bilayer patches (PC 34:1 or ER lipids). In MD simulations of free bilayer patches with FAM134B-RHD, we observed patch-closure and vesicle formation (Supplementary Figs. 9 and 10; Supplementary Note 6). The observed bilayer-to-vesicle transitions are a consequence of two effects: (i) the induction of membrane curvature by the protein and (ii) the unstable edge of the discontinuous bilayer disc (Supplementary movie 1). To decouple these two effects, we employed a bicelle system (DMPC + DHPC) with minimal edge tension (see the section “Methods”) to study curvature induction.

**Fig. 3:**
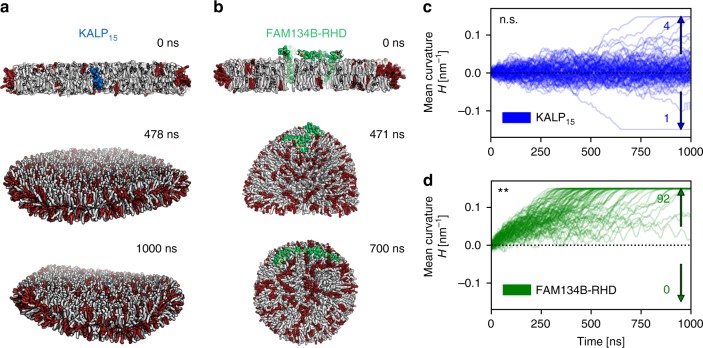
Bicelle-to-vesicle transitions. **a**, **b** Snapshots showing curvature of bicelles containing DMPC (gray) and short chain DHPC lipids (red) (**a**) with KALP_15_ peptide (blue) and (**b**) FAM134B-RHD (green). **c** KALP_15_-containing bicelles remain flat with low curvature (|*H*| = ±0.005 nm^−1^), rarely displaying vesiculation events (5/96) within 96 simulations of 1000 ns each. **d** FAM134B-RHD actively curves the bicelle to form vesicles in repeated runs (92/95). FAM134B-RHD induces strong positive curvature along the cytoplasmic leaflet resulting in positively curved vesicles (*H* = +0.16 nm^−1^). **c**, **d** Curvature time-traces (blue/green, smoothed running averages over 11 ns widows) from individual replicates quantify the bicelle shape transformation process during simulations. **Denote and n.s. denote the one-tailed probability in binomial tests for bias in number of vesiculation events with positive and negative bicelle curvatures

In the bicelle, short-chain DHPC lipids (C7) localize to the rim of the predominantly flat DMPC bilayer (C14) and stabilize the open edge (Supplementary Fig. 11a, b). Empty bicelles thus remain relatively flat (|*H*| ≤ 0.03 nm^−1^) and stable on the MD time-scale (up to 2 μs; Supplementary Fig. 11a; Supplementary movie 2). Control bicelle systems with KALP_15_ also remain more or less flat (Fig. 3a). KALP_15_ and empty bicelles seldom vesiculate (5/96 and 2/95, respectively) spontaneously and without clear directionality (Fig. 3c; Supplementary Fig. 11c). By contrast, FAM134B-RHD induces curvature of edge-stabilized bicelles (Fig. 3b). Bicelles with the RHD undergo complete vesiculation with highly curved intermediates (Fig. 3b; Supplementary movie 3). In nearly all simulations, we observed bicelle-to-vesicle transitions within the simulation time (92/95 runs). All transitions involved positive curvature (+*H*), i.e., curving away from the cytoplasmic leaflet (*n*_+_ = 92; Fig. 3d).

We express the driving force for protein-induced curvature induction by measuring rates of vesicle formation (Supplementary Table 5). We estimate that FAM134B-RHD accelerated vesicle formation by factors of 2 for open bilayer discs and 160 for bicelles (Supplementary Table 5). By comparison, the KALP_15_ peptide showed only a small acceleration in vesicle formation (1.35 for bilayer discs and 5 for bicelles). Accelerated vesicle formation in the presence of FAM134B-RHD is a result of directed curvature induction. High occupancy of FAM134B-RHD at the cusp (apex) of deformed bilayer discs and bicelles indicates a direct role of the RHD in curvature induction (Fig. 3b; Supplementary Figs. 9 and 10). Thus, the in silico curvature assays demonstrate the curvature induction capacity of FAM134B (Fig. 3).

To identify the structural elements of the RHD responsible for directional curvature induction, we examined vesiculation rates of bilayer discs and bicelles in the presence of various FAM134B-RHD fragments (Supplementary Figs. 12–14; Supplementary Note 7). By monitoring both the sign of membrane curvature (*H*(*t*)) and rates of vesicle formation from a large number of simulations (Supplementary Table 4), we determined the ability of individual fragments to induce specific and directional curvature effects (Supplementary Table 5). We found that individual TM hairpin fragments (TM12 or TM34) do not efficiently induce directional curvature in bilayers or bicelles, whereas fragments containing AH_L_ or AH_C_ induce specific and directional curvature resulting in enhanced vesiculation rates.

### FAM134B-RHD senses membrane curvature

**Fig. 4:**
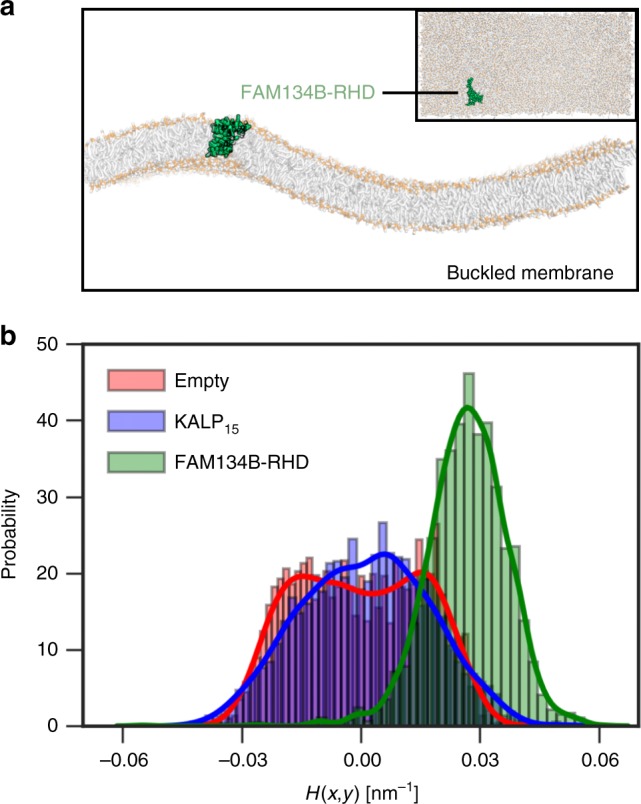
Curvature sensing by FAM134B-RHD. **a** Cut through the simulation box along the *xz* plane (inset top view) showing the buckled lipid bilayer (orange phosphate beads), with excess area (≈17 nm^2^) under edge compression. Diffusion of curvature-inducing proteins such as FAM134B-RHD (green) in the buckled membrane enables curvature sampling and estimation of intrinsic curvature preferences (see the section “Methods”). We tracked the position of proteins (*x*, *y*) along the buckle, and quantified the curvature preference (principal, mean and Gaussian; see Supplementary Fig. 15). **b** Histograms of mean curvature, *H*(*x, y*), sampled by FAM134B-RHD (green) in coarse-grained simulations (1 ns intervals for 20 μs) indicate a preference for highly curved regions of the buckle. By contrast, the KALP_15_ peptide (blue) samples regions with lower curvature along the buckle. The local mean curvature field of the empty buckled membrane (red) is obtained by random sampling of points in the *xy* plane (see Supplementary Fig. 16)

We determined the intrinsic curvature preference of FAM134B-RHD by simulating a buckled bilayer under lateral compression with periodic boundary conditions (see Supplementary Methods; Supplementary Table 6). The membrane adopts a sinusoidal folded-carpet structure with a range of local mean curvatures (*H*(*x, y*) = −0.05 to 0.05 nm^−1^). Lateral protein diffusion within this buckled membrane permits curvature sampling and quantification of local curvature preference (Fig. 4). Accordingly, we tracked protein center-of-mass positions and orientations, and computed the associated local curvature of the buckled membrane profiles (see Supplementary Methods; Supplementary Figs. 15–18).

We found that FAM134B-RHD strongly prefers regions of high local curvature (Fig. 4a; Supplementary movie 4). FAM134B-RHD was initially placed in a region of low mean curvature and oriented such that its internal orientation (long axes of AH_L_ and AH_C_) was parallel to the direction of the membrane buckle (*x*-axis). We found that FAM134B-RHD further enhances the curvature of the buckle, and occupies regions of high local mean curvature (*H*(*x, y*) ≈ 0.026 nm^−1^); Fig. 4b, green histograms). In control simulations with the KALP_15_ peptide, we observed a preference for surfaces with small mean curvature (*H*(*x, y*) ≈ 0.0011 nm^−1^); Fig. 4b, blue histograms). Detailed 2D histograms of preferred principal curvatures (*k*_1_ and *k*_2_) revealed that FAM134B-RHD associates with regions of positive principal curvatures, resembling local bulges of the buckle (Gaussian curvature *K*_G_ > 0 corresponding to ellipsoidal vesicle shapes; Supplementary Fig. 16). Control simulations with KALP_15_ showed that the peptide associates with regions of both positive and negative principal curvatures, indicating preference for local gentle saddle-like regions of the buckle (*K*_G_ < 0; Supplementary Fig. 16; Supplementary movie 5).

**Fig. 5:**
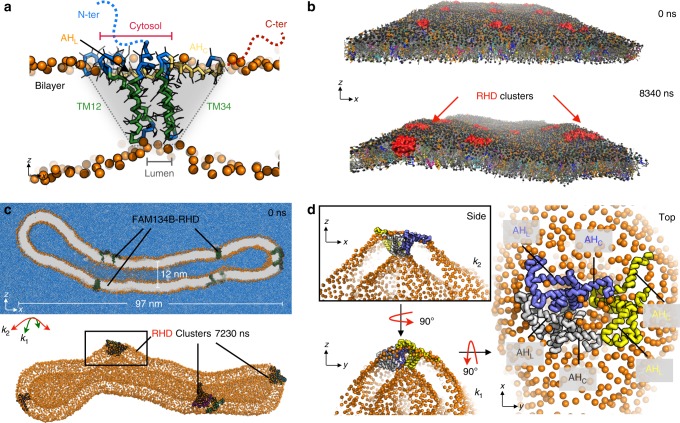
RHD unique topology drives curvature and clustering in membranes. **a** Snapshot from coarse-grained simulation. The FAM134B-RHD forms a wedge-shaped protein inclusion (gray shade) in the membrane (orange PO4 beads). Local bilayer thinning by short hairpins (TM12 and TM34; green) promotes inter-hairpin interactions (blue) at the luminal leaflet (see Supplementary Fig. 19). AH_L_ (orange/yellow sticks) separates the two hairpins on the cytosolic leaflet and, along with AH_C_ (yellow sticks) enhances curvature. **b** Simulation snapshots showing local clustering of multiple FAM134B-RHD molecules (red) on model ER membrane under periodic boundary conditions (see Supplementary Fig. 21). **c** Cross section of a closed tubular structure (*k*_1_ ≈ 0.16 nm^−1^; *k*_2_ ≈ 0 nm^−1^; gray with orange PO4 beads) containing 10 FAM134B-RHD molecules simulated in explicit solvent (~3.6 × 10^6^ water beads; blue). Deformed tubule structure (below) after ≈7 μs showing organization of RHDs into three local clusters. **d** Zoom-in on the boxed cluster containing three RHDs (see Supplementary Fig. 23 for other clusters). Side views (left) of the RHD cluster shaped as an inverted pyramid display locally curved tubule surface along principal axes, *k*_2_ and *k*_1_. Top view (right) showing the organization of AHs at the base of the pyramid. Two RHDs (blue and gray) align their AHs perpendicular to the tube axis, while the AHs of the third RHD (yellow) are parallel to the tube axis

In long simulations of the intact RHD (20 μs) in flat bilayers (POPC or ER-lipids; Supplementary Table 2), we observed the formation of a wedge-shaped structure (Fig. 5a). The two short TM hairpins (TM12 and TM34) were pulled together, likely to minimize the overall membrane perturbation. This arrangement was dynamic, bridged by a flexible linker fragment (RMSD ~  0.6–0.8 nm) between the two hairpins (Supplementary Fig.19a). The two TM hairpins interacted via their hydrophilic luminal loops, which formed a narrow tip at the luminal leaflet (*d*_TM12−TM34_ = 1.51 ± 0.67 nm; Supplementary Fig. 19b). On the cytosolic face, the AH_L_ kept the two hairpins apart (*d*_TM12−TM34_ = 2.83 ± 0.45 nm; Supplementary Fig. 19b). The organization of individual hairpins and dynamic tertiary contacts between them are also consistent with predicted interacting residue pairs from sequence covariance data (Supplementary Note 5; Supplementary Fig. 20). The resulting structure has a narrow luminal contact and a more extended cytosolic footprint resembling a wedge. The asymmetry of the wedge curves the bilayer strongly away from the cytosolic leaflet. Similar wedge-like intermediate structures were also observed in the bicelle and membrane-patch vesiculation simulations. Overall these data confirm the ability of FAM134B-RHD to sense positively curved membrane regions and to induce curved structures.

### FAM134B-RHD clusters amplify membrane deformation

Even though single FAM134B-RHD can actively induce curvature of isolated bilayer/bicelle patches and display local curvature-sensing functions, the formation of autophagic puncta suggests that RHD clusters are responsible for global remodeling of membranes during ER-phagy^7^. In our membrane patch simulations (Fig. 3), we started from a metastable system, which made it possible to observe spontaneous vesiculation. However, vesiculation from a flat membrane or tubule likely requires the action of multiple proteins.

To understand membrane remodeling by FAM134B-RHD clusters, we simulated cluster formation on flat and curved membranes (see Supplementary Methods; Supplementary Table 6). In a simulation of a large flat membrane (ER-lipids, 62 × 62 nm^2^; Fig. 5b) with 9 FAM134B-RHD molecules embedded, despite the attenuation of bilayer undulations by the boundary conditions, the bilayer was locally curved. The membrane displayed several local deformations, especially close to embedded RHD molecules and transiently formed RHD clusters. Over the course of 10 μs, we observed the formation and dissociation of several RHD clusters associated with local membrane bulging. The formation of RHD clusters in flat membranes is dynamic, with ~2–3 RHD molecules coming close together for short periods of time (~0.5−1 μs) with no specific geometry and orientation (Supplementary Fig. 21). We reasoned that the curvature-sensing function of RHDs could stabilize transiently formed RHD clusters in highly curved regions of the bilayer.

We directly tested the role of RHD topology in curvature-mediated protein sorting and clustering in simulations of buckled bilayers with two RHD molecules. We embedded two RHD molecules in correct and inverted topology, respectively (Supplementary Fig. 22). In simulations with correctly inserted RHDs, the two protein molecules diffused to the region of high local curvature and formed a loosely organized cluster on top of the buckle (Supplementary Fig. 22a). By contrast, when the second RHD was on the other membrane side, the two proteins stayed apart from each other (Supplementary Fig. 22b), localized on opposite peaks of the sinusoidal buckle. This indicated that the correct membrane topology is crucial for protein sorting and clustering in curved membranes, and essential for FAM134B function.

Simulations of 10 FAM134B-RHD molecules embedded in a closed tubular structure gave us further insight into the role of protein clustering and associated membrane remodeling (Fig. 5c, top). In the cylindrical section of the tubule, the principal curvatures were *k*_1_ ≈ +0.08 nm^−1^ and *k*_2_ ≈ 0 nm^−1^, respectively. The 10 RHD molecules were initially placed away from each other in an orientation such that their principal axes were parallel or perpendicular to the tubule’s central axis (see Supplementary movie 6; Supplementary Fig. 23a). During 10 μs of simulation, we observed the formation of several FAM134B clusters. The tubule structure was severely deformed with three distinct RHD clusters and a single RHD molecule (Fig. 5c, bottom). The clusters along the tubule axis, induced strong local deformations in both principal directions (i.e., both *k*_1_ > 0 and *k*_2_ > 0; Fig. 5d and Supplementary Fig. 23d). The third cluster and a lone FAM134B-RHD molecule occupied the highly curved caps of the tubule (Fig. 5c, bottom-left; Fig. 5d and Supplementary Fig. 23c).

The RHD clusters adopted unique inverted-pyramid-like structures in curved bilayers (Fig. 5d and Supplementary Fig. 23c, d). Three individual wedge-shaped proteins clustered and organized into a larger inverted-pyramidal structure. The six TM hairpins (from three RHDs) clustered closely with interactions mediated by their hydrophilic luminal loops forming the conical tip of the inverted pyramid on the luminal face (Fig. 5c, side views), while their amphipathic helices organized into a shallow membrane-embedded base of the inverted pyramid on the cytosolic face. This arrangement was preserved in all three RHD clusters observed and resulted in enhanced local bending of the tubular bilayer in both principal directions. Clustering therefore enhances the bi-directional curvature preference observed for individual FAM134B-RHDs.

### FAM134B-RHD remodels liposomes and fragments ER

**Fig. 6:**
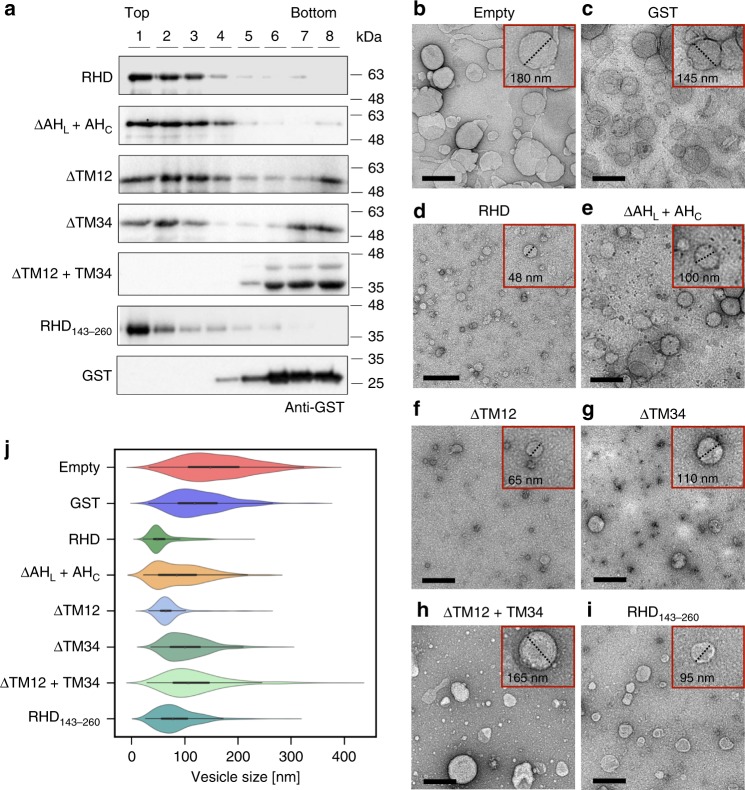
RHD structure determines in vitro membrane binding and liposome remodeling activity. **a** Liposome co-flotation assay to evaluate membrane-binding properties of FAM134B-RHD and various deletion mutants (see the section “Methods”). Purified protein samples were incubated with liposomes for 2 h at 37 °C and subjected to flotation on a sucrose cushion (top to bottom, 1–8) followed by SDS–PAGE and western blotting with anti-GST antibody. **b**–**i** Representative nsTEM micrographs of remodeled proteoliposomes (scale bars, 200 nm). Empty liposomes (**b**) were remodeled by incubation after addition of purified (**c**) GST, (**d**) wild-type RHD, (**e**) ΔAH_L_ + AH_C_, (**f**) ΔTM12, (**g**) ΔTM34, (**h**) ΔTM12 + TM34, and (**i**) RHD_143–260_ for 18 h at 22 °C. Insets (red squares); magnified micrographs showing examples of representative proteoliposomes with diameters measured (dotted lines) using ImageJ. **j** Violin plots show the measured proteoliposome size-distributions (*n* = 300 each) from nsTEM images. Violins shows a central boxplot (median with interquartile range, black lines) along with mirrored histograms on either sides (colored)

To elucidate the role of the RHD in direct membrane binding and remodeling, we cloned, expressed, and purified wild-type FAM134B-RHD along with a set of rationally designed deletion constructs (ΔTM12, ΔTM34, ΔTM12 + TM34, ΔAH_L_ + AH_C_, and RHD_143–260_). We first investigated the in vitro membrane-binding ability of the purified proteins using liposome flotation assays (Fig. 6a). We were able to detect the intact RHD in the liposome fraction (top fractions, 1–4), indicating proper membrane binding and insertion. Proteins with only a single TM hairpin segment (either ΔTM12 or ΔTM34) were detected in all the fractions (top and bottom, 1–8), indicating reduced membrane binding and insertion into liposomes. Removal of the entire TM region (ΔTM12 + 34), abolished membrane binding and insertion, similar to GST (Fig. 6a, bottom fractions 5–8). By contrast, deletion of the amphipathic helices (ΔAH_L_ + AH_C_) did not affect membrane binding significantly (top fractions, 1–5). These experiments indicate that at least a single TM hairpin fragment is required for stable membrane binding and anchoring into liposomes.

Next, to test the curvature induction and membrane shaping by the RHD, we performed in vitro liposome remodeling experiments (see the section “Methods”). We reconstituted empty liposomes (~200 nm diameter) with purified protein and imaged them by negative-stain transmission electron microscopy (nsTEM; Fig. 6b–i). We quantified the protein-induced membrane shaping by measuring the sizes of the reconstituted proteoliposomes (Fig. 6j). We found that the wild-type protein with an intact RHD drastically remodeled larger liposomes into smaller vesicles (Fig. 6d). The resulting proteoliposomes were highly curved and more homogeneous with a narrow distribution (green, Fig. 6j). This behavior is dose-dependent and increased with increasing protein concentration (Supplementary Fig. 24). By contrast the addition of either purified GST (Fig. 6c) or ΔTM12 + TM34 (Fig. 6h) to empty liposomes (Fig. 6b) showed no remodeling behavior (red, blue and light green in Fig. 6j), consistent with the expected membrane-binding ability of GST and ΔTM12 + TM34 (Fig. 6a, Supplementary Fig. 24a, c).

Deletion of the first TM hairpin (ΔTM12; Fig. 6f) led to smaller proteoliposomes with a narrow distribution (light blue in Fig. 6j). These proteoliposomes were slightly larger in comparison to the wild-type (green), indicating a minor loss of membrane-remodeling activity. Deletion of the second TM hairpin segment (ΔTM34; Fig. 6g) resulted in substantially larger proteolipososomes with a wider distribution (sea green in Fig. 6j), indicating a significant loss of the membrane remodeling activity. Interestingly, the deletion of both AH_L_ and AH_C_ (ΔAH_L_ + AH_C_; Fig. 6e) also resulted in larger proteoliposomes with wide distribution (orange in Fig. 6j), indicating that AH fragments are also required for efficient membrane shaping. The TM34 segment is flanked by amphipathic helices on both ends. The contribution of the AH_L_-TM34-AH_C_ motif to membrane shaping was further assessed by generating a truncated version, RHD_143–260_. This variant retained the ability to remodel liposomes (RHD_143–260_; Fig. 6i; dark cyan in Fig. 6j), though less than the intact RHD. These results indicate that the presence of both the hairpins along with amphipathic helices is essential for maximal membrane shaping and remodeling activity.

**Fig. 7:**
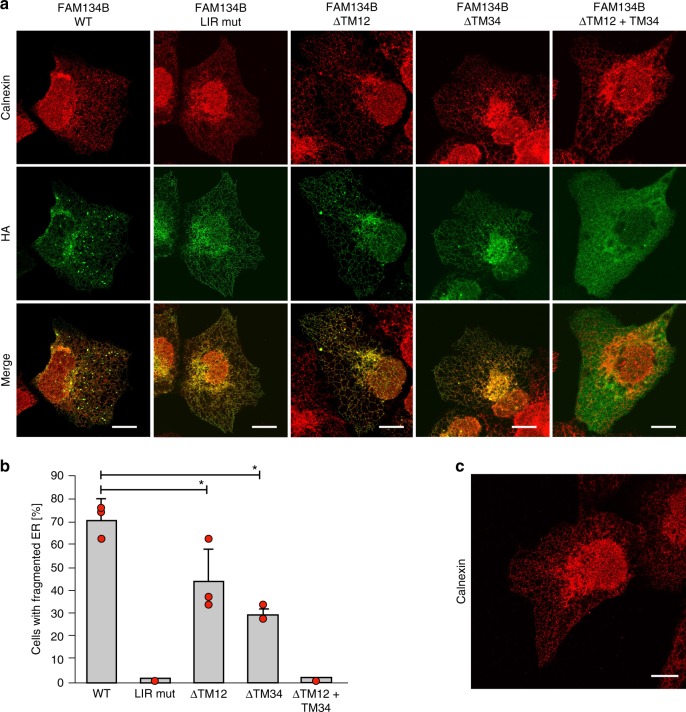
TM hairpins of FAM134B are required for ER fragmentation. **a** Immunofluorescence of HA and endogenous calnexin in U2OS cells transiently overexpressing HA-tagged FAM134B (left to right): wild-type (WT), LIR mutant (LIR mut), single hairpin deletions (ΔTM12 and ΔTM34), and the double hairpin deletion (ΔTM12 + TM34). Scale bars 10 μm. **b** Quantification of U2OS cells with fragmented ER (≥5 ER fragments per cell) after transient over-expression of wild-type and mutant forms of FAM134B. Error bars indicate s.d. from triplicates (red filled circles) and *Denotes *p* < 0.05 in two-sample Student’s *t*-tests. **c** Immunofluorescence of endogenous calnexin in un-transfected U2OS cells. Scale bar 10 μm

We related the different FAM134B-RHD structural elements to selective ER-phagy on the basis of in-cell experiments (Fig. 7). We over-expressed wild-type and deletion constructs of FAM134B in U2OS cells and evaluated the status of the ER fragmentation 24 h after transfection. The full-length protein (WT) and the mutant LIR (LIR mut) served as positive and negative controls, respectively^7^. A confocal microscopy-based assay confirmed the formation of characteristic autophagic puncta and the induction of relevant ER fragmentation in cells expressing the wild-type protein (WT, Fig. 7a, b). In cells expressing the LIR mutant, we observed that puncta formation and ER-fragmentation was completely abolished (LIR mut, Fig. 7a, b). Deletion of single TM hairpin segments from the RHD significantly reduced the extent of ER fragmentation (either ΔTM12 or ΔTM34, Fig. 7a, b). However, truncated FAM134B, was still localized in the ER as shown by the overlap with calnexin signal (Fig. 7a, c; red). Upon removal of both TM hairpin fragments, we found that the protein could no longer localize to the ER and consequently lost its ability to fragment the ER (ΔTM12 + TM34, Fig. 7b). Single deletions of AH segments (ΔAH_L_ or ΔAH_C_) and double deletion (ΔAH_L_ + AH_C_) did not affect the extent of ER fragmentation (Supplementary Fig. 25).

Fragmented RHD structures affect in vitro liposome remodeling (Fig. 6e–i) and slow down in silico membrane curvature induction (Supplementary Figs. 12–14). We found that, FAM134B-Q145X, a naturally occurring genetic truncation responsible for severe sensory neuropathy, delayed in silico curvature induction (acceleration factor 1.41 for bilayer patch; Supplementary Fig. 26; Supplementary Table 5). This truncation variant is similar to the N-terminal cleavage product of FAM134B (R142X) during Zika viral infection. Both the FAM134B-Q145X variant and the TM12 fragment display comparable in silico vesiculation behavior (acceleration factors 1.41 and 1.36; Supplementary Figs. 26, 12b, 13a) and are also inefficient in remodeling large liposomes (ΔTM34 and RHD_143–260_; Fig. 6g, i). By contrast, intact FAM134B-RHD is required and essential for in vitro liposome remodeling and cellular ER-fragmentation.

## Discussion

The integration of molecular modeling and extensive MD simulations allowed us to build a structural model of the RHD of FAM134B. Our assembly of the RHD followed principles of membrane protein organization^23^, consistent with experimentally verified topologies of closely related reticulon proteins^21,22^. This relation to reticulons and the relatively simple structure of the components (i.e., helices and helical hairpins) enabled structure modeling by fragment assembly followed by MD simulations. Our simulations show a dynamic organization of the RHD TM region. Inter-hairpin interactions are only transient, mediated by luminal loops, with TM12 and TM34 separated on the cytosolic side by the AH_L_ spacer.

FAM134B actively remodels membranes. Previous liposome flotation assays and freeze fracture EM studies demonstrated that FAM134B binds to liposomes via its RHD and induces smaller vesicles (≈100 nm; *H* ≈ 0.02 nm^−1^)^7^. With in silico curvature assays, we simulated the active protein-mediated curvature induction process. Bilayer patch-closure simulations have been used previously to study elastic properties of the membrane^24,25^. Here, we used the bicelle system to probe the active curvature induction capacity of an embedded protein inclusion. Using our RHD model, we were able to demonstrate active curvature induction in model membranes.

The RHD topology is crucial for curvature induction. The TM hairpins and the amphipathic helices have previously been implicated in membrane curvature sensing and induction^19,26^. In case of isolated RHDs, our data indicate that membrane curvature is predominantly induced by the synergistic action of the two TM hairpins and amphipathic helices. They produce an overall wedge-shaped membrane inclusion, inducing strong preferential curvature away from the cytoplasmic leaflet. Amphipathic helices are employed by several proteins to induce and sense membrane curvature^27^. They act as shallow inclusions, enhancing asymmetric bilayer stretching and scaffolding to induce curvature^28–31^. The importance of the C-terminal AH in RHDs was first observed in a C-terminal deletion of YOP1 (R137X)^21^. Complete deletion of AH segments in yeast YOP1 and Arabidopsis RTN13 affects in vitro and in cell tubule-shaping function^19,20^. In the case of isolated FAM134B-RHDs, the amphipathic helices dominate membrane shaping based on both our bicelle-vesiculation simulations and our liposome-remodeling assay.

A study of in vitro curvature induction by the yeast RTN1-M91 mutant has also implicated the TM regions^21^. Other TM mutations in RHDs showed that the topology and short length of the helical hairpins are important for tubule shaping in cells^26^. In support of TM contributions to membrane shaping, we observed TM12 and TM34 of FAM134B monomers to form distinct wedges that became even more pronounced for FAM134B oligomers. In liposome-remodeling experiments, both hairpins (TM12 + TM34) are required for maximal curvature induction.

The RHD does not only actively induce positive curvature on ER membranes, but also senses high local mean curvature. From the simulations of FAM134B-RHD on sinusoidal membrane surfaces, we could deduce its curvature preference. The high local curvature preference of FAM134B-RHD has explicit consequences for ER-phagy. Several studies report on long-range attractive forces between embedded proteins mediated by membrane deformations causing local protein clustering and self-organization^32–37^. Curvature-mediated protein sorting may be an intrinsic mechanism to concentrate curvature inducing and sensing proteins in the ER. Mutations in TM regions of RHDs implicate a role in protein localization and oligomerization^26,38^. Indeed, the bright intense fluorescence of ER puncta containing intact FAM134B-RHD indicates protein clustering. This may be a consequence of the curvature-sensing functions of FAM134B in ER membranes (with clustering favored energetically to minimize membrane deformations overall) or curvature induction (sequestered by LIR–LC3 interactions), or both. Using simulations of multiple RHDs, we showed the formation of RHD clusters in a curvature-dependent fashion. These RHD clusters organize into inverted-pyramid-shaped structures that strongly deform the closed tubular membrane.

Curvature induction and sensing by the RHD assist ER remodeling. Over-expression of FAM134B WT induces ER-fragmentation, whereas FAM134B with disrupted RHDs (ΔTMs) display reduced ER-fragmentation, implicating a direct role for the RHD in membrane deformation and protein clustering^7^. Intact RHDs are also important for the interaction between FAM134B and calnexin during selective elimination of unfolded procollagen from the ER^39^. In the disease-causing genetic truncation variant FAM134B-Q145X, the two amphipathic helices, the hairpin TM34, and the C-terminal LIR are lost^14^. We showed that a single TM hairpin is sufficient to anchor mutant FAM134B into the ER-membrane. The non-structural viral protease NS2B disrupts the RHD structure by proteolytic cleavage after R142, compromising clearance of viral proteins through selective ER-phagy during Zika and Ebola viral infections^16^. The loss of the structural integrity of FAM134B-RHD thus has drastic consequences, leading to genetic diseases and exploitation by viruses^15^.

**Fig. 8:**
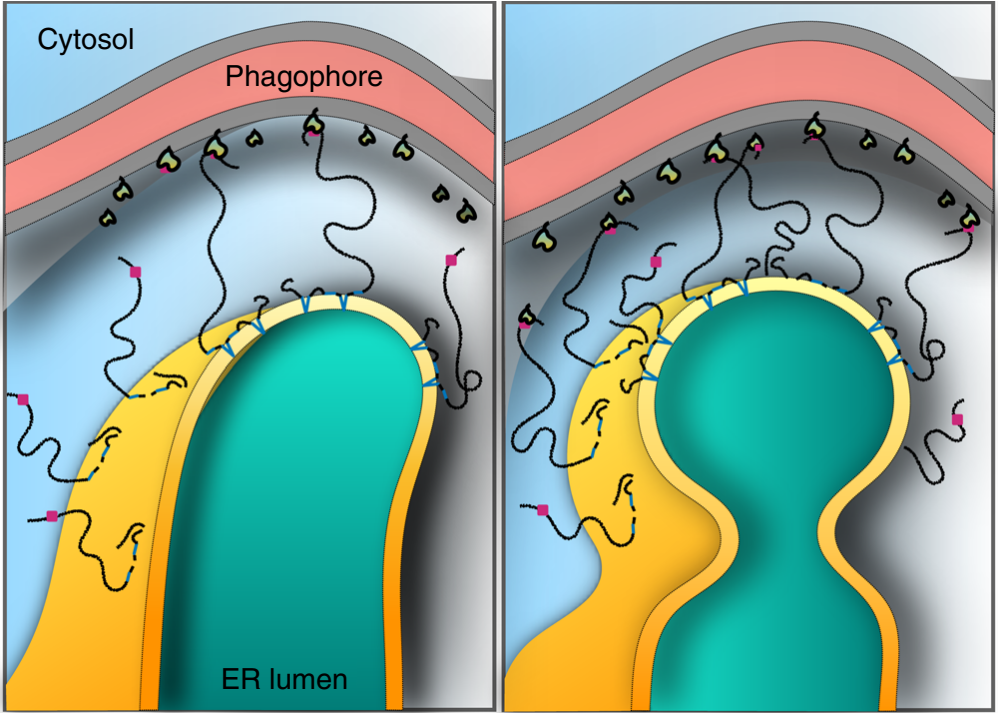
Role of FAM134B-induced membrane curvature in ER-phagy. FAM134B induces high curvature in the ER membrane (yellow/orange). The high intrinsic curvature preference of the FAM134B-RHD (dark blue helices) enables its partitioning and clustering specifically to perinuclear ER. The presence of N-terminal and C-terminal disordered fragments (black lines) enhances local curvature (yellow gradient) by providing additional steric forces to bend the ER membrane. Using the C-terminal LIR (pink box), FAM134B also forms a physical bridge between the ER membrane (orange) and phagophore membrane (gray)-associated LC3-PE (yellow/green). This interaction provides additional forces required for scission and fragmentation of ER membranes. High local membrane curvature induced by FAM134B-RHD thus lowers the barrier for membrane budding and subsequent pinch-off in FAM134B-enriched ER

Recent high-resolution imaging techniques revealed a dense and dynamic network of membrane structures in the highly curved peripheral ER^3^. Our simulation results support a model in which FAM134B mediates non-specific curvature-induced protein clustering and accumulation in the ER, primarily through its curvature-sensing function. As a result, highly curved regions of the ER, more specifically edges of sheets, would be loaded with FAM134B. The RHD induces active membrane curvature along both principal directions enabling the formation of spherical or ellipsoidal membrane buds from the ER membrane (Fig. 8).

External forces mediated by interactions between autophagosomes and FAM134B could provide pulling forces on the highly curved ER membrane and amplify the effect of the RHD (Fig. 8). We reason that LIR-mediated pulling by the C-terminal disordered tail provides additional forces, aided by the strong membrane deformations induced by FAM134B-RHD, to enable vesicle budding and subsequent pinch-off from ER-membranes. Thus, localized regions of the ER enriched with FAM134B become hot-spots for selective ER-phagy. This is consistent with findings that mutation of LIRs in FAM134B and RTN3 block ER-phagy-mediated sequestration and fragmentation of the ER^7,9^.

FAM134B effectively combines two functions to maximize the autophagic response: active curvature induction and sensing by the RHD, and direct physical binding to phagophore via the LIR. Direct linking of curvature inducing and sensing domains with LIRs elevates membrane curvature by providing forces to bend membranes. This combination of features is common with the selective ER-phagy receptors human RTN3 and yeast ATG40^9,40^. Viral proteins like influenza M2 exploit this combination of features by employing curvature-inducing amphipathic helices tethered to LIRs to subvert autophagy^41^.

In conclusion, we developed a structural model for the RHD of FAM134B that provides mechanistic insights into curvature induction and sensing in the ER and explains in vitro liposome binding and remodeling. Simulation and analysis methods developed here make it possible to model more complex and large-scale membrane remodeling processes. We hope that our structural model can serve as a basis for detailed experimental characterizations of the structure and function of FAM134B-RHD and its oligomeric assemblies in vitro and in cells.

## Methods

### Sequence analysis and annotation

Domain annotation for FAM134B (renamed RETR1, UniProt code: Q9H6L5) was performed by BLAST search of its full-length sequence against all HMM profiles in the Pfam database^42^ and PSSM profiles in the Conserved Domain Database^43^. Homologs of FAM134B were identified from the UniProt database^42^ using five iterations of PSI-BLAST^44^. Hits were filtered (using an *E*-value cut-off of 10^−4^, sequence identity range 30–90%, and query coverage ≥70%) and clustered to remove redundancy using CD-HIT^45^. Multiple sequence alignments (MSA) of FAM134B homologs were generated using MAFFT^46^, and used to compute residue-wise conservation scores (Supplementary Fig. 27)^47^. Profile-based alignments between the FAM134 family and the canonical RTN family were performed using ALignME^48^. TM, cytoplasmic, and luminal regions of the protein were identified from consensus membrane topology predictions using TOPCONS^49^. Secondary structure assignments were obtained using PSI-pred and JNetpred^50^. Discontinuities and bends within the predicted TM-regions were identified using TMKink^51^. Amphipathic helices were recognized by screening extra-membrane, helical regions with large hydrophobic moment using Heliquest^52^. Hydrophobicity and hydrophobic moments were computed for predicted helical regions from homologs of FAM134B and RTN family using in-house scripts.

### Structural modeling of FAM134B-RHD

Due to unavailability of a homolog of the RHD with known structure in the PDB, we used a fragment-based approach. Based on sequence annotation, residue conservation, predicted secondary structure, and predicted membrane topology, the structured region of FAM134B (80–260) was sub-divided into five overlapping segments: N-terminal overhang, TM12, linker, TM34, and C-terminal segment (Supplementary Table 1). We used RaptorX^53^ to identify template fragments of similar sequence with preserved secondary structure profiles (helix or helical hairpins) and known structure. The highest-scoring fragment alignments were used to build rough 3D fragment models using RaptorX, covering the region from position 80 to 260. These initial fragment structures were first subjected to conformational sampling and refinement using extensive CG and all-atom MD simulations in the presence of lipid bilayers (see Supplementary Table 2 and immediately following text). Long-lived refined fragment structures were then assembled into a single FAM134B-RHD model by remodeling overlapping segments. The loop modeling protocol of Modeller^54^ was used to stitch the fragment junctions together to obtain a structure of FAM134B-RHD (Supplementary Note 5).

### MD simulations

All-atom MD simulations were performed for refinement of fragment models and the assembled FAM134B-RHD. CG MD simulations of FAM134B-RHD(s) were performed to study its structure and flexibility, its membrane curvature induction and sensing function, and curvature-dependent sorting and cluster formation (see Supplementary Methods).

For all-atom MD simulations, the position and orientation of FAM134B-fragment models and assembled FAM134B-RHD with respect to the bilayer were obtained using the OPM database and PPM web server^55^. The resulting structures were inserted into pre-equilibrated POPC (16:0–18:1 PC) bilayers, solvated with TIP3P water and 150 mM NaCl using CHARMM-GUI: membrane builder^56,57^. After initial energy minimization, six rounds of short equilibration runs with position restraints on protein atoms were performed. Following equilibration, the systems were simulated with a 2 fs time step for ≈3 μs using the CHARMM36m force field^58^. The system temperature and pressure were maintained at 310 K and 1 atm using the Nosé–Hoover thermostat^59–61^ and the semi-isotropic Parrinello–Rahman barostat, respectively^62^.

CG MD simulations were performed using the MARTINI model (version 2.2)^63,64^. We first constructed initial CG structures of the FAM134B-fragments and FAM134B-RHD. DSSP^65^ and PSI-pred^50^ assignments were used to generate back-bone restraints that preserve local secondary structure for FAM134B-RHD and FAM134B-fragment models, respectively. CG models were embedded into POPC (16:0–18:1 PC) bilayers or model ER membranes spanning the periodic simulation box in the *xy* plane. Initial configurations for each system were assembled, and then solvated with CG-water containing 150 mM NaCl using the insane.py script^66^. Each system was first energy minimized and equilibrated using the Berendsen thermostat and barostat^67^ along with position restraints on protein backbone beads followed by production runs with a 20-fs time step. System temperature and pressure during the production phase were maintained at 310 K and 1 atm with the velocity rescaling thermostat^68^ and the semi-isotropic Parrinello–Rahman barostat^62^, respectively. All simulations were performed using gromacs (version 4.6.5)^69^ (Supplementary Tables 2 and 3).

### Plasmids, antibodies, and cell culture

For bacterial expression and purification, WT pGEX6P1-FAM134B-RHD (RHD) construct was obtained by subcloning a codon-optimized FAM134B gene (residues 1–260) into the pGEX6P1 expression vector using BamHI and SalI cloning sites. The codon-optimized synthetic gene of FAM134B was produced by Genscript. The deletion constructs of FAM134B-RHD, lacking either one or two TM hairpins (ΔTM12, Δ92–134; ΔTM34, Δ194–236; and ΔTM12 + TM34, Δ92–134, 194–236) or both amphipathic helices (ΔAH_L_ + AH_C_, Δ165–188, 238–260) were generated by site directed-mutagenesis (see Supplementary Methods for list of primers used) using the QuickChange method (Agilent Technologies). Site-directed mutagenesis was also used to generate the the N-terminal truncated variant by removing the first 142 residues (RHD_143–260_). Wild-type and deletion mutants of FAM134B were cloned as glutathione-S-transferase (GST) fusion proteins containing the endogenous N-terminal fragment (1–80) to achieve maximal protein expression.

FAM134B plasmids for mammalian expression were obtained by subcloning FAM134B Orf, fused with the HA tag at the C-terminus, into the pcDNA3.1(+) vector (Invitrogen) from pOTB7-FAM134B (MHS1011-9199640) using HindIII and XhoI cloning sites. FAM134B LIR mut and deletion constructs ΔTM12, ΔTM34, ΔTM12 + TM34, ΔAH_L_, ΔAH_C_, and ΔAH_L_ + AH_C_ were generated by site direct mutagenesis using pcDNA3.1(+)-FAM134B-HA as a template (Supplementary Fig. 25a; Supplementary Methods). U2OS cells (ATCC, HTB-96) were cultivated in standard DMEM media (Gibco) further supplemented with 10% fetal calf serum (Gibco) and containing 100 μg/ml penicillin and streptomycin (Thermo Fisher Scientific). Cells were maintained at 37 °C with 5% CO_2_ and were regularly tested for the presence of mycoplasma using LookOut Mycoplasma qPCR Detection Kit (SIGMA). U2OS cells are mycoplasma negative. Cells were regularly tested for the presence of mycoplasma using LookOut Mycoplasma qPCR Detection Kit (SIGMA).

### Protein expression and purification

The *E. coli* C41(DE3) strain (SigmaAldrich, #CMC0021) was used for heterologous protein expression and purification of FAM134B-RHD variants. Bacterial cells were transformed with the different plasmids and grown in 12 l of lysogenybroth (LB) medium with 100 μg/ml ampicillin on shakers (220 rpm), at 37 °C until the cell density reached an OD between 0.6 and 0.7 (600 nm). Then, protein expression was induced with 0.25 mM isopropyl *β*-d-1-thiogalactopyranoside (IPTG) for 16 h at 18 °C. Cells were then harvested by centrifugation and following re-suspension of cell-pellets in 120 ml of ice-cold PBS buffer (pH 7.4) with protease inhibitor cocktail (Roche). Cells were lysed by sonication and centrifuged at 10,000 × *g* for 15 min. The resulting supernatant was further centrifuged at 80,000 × *g* for 2 h. The cell pellet containing the membrane fraction was then solubilized in 75 ml ice-cold PBS (pH 7.4) with 0.05% (w/v) n-Dodecyl *β*-d-maltoside (DDM) for 2 h at 4 °C. For protein purification of the different membrane-bound FAM134-RHD variants, the spin-cleared membrane fraction was subsequently loaded onto a Glutathione SepharoseTM 4 Fast Flow resin (GE Healthcare), pre-equilibrated in PBS with 0.05% (w/v) DDM. Proteins were eluted in PBS (3× column volumes, pH 7.4) containing 0.025% (w/v) DDM and 15 mM reduced glutathione (Carl Roth). Control GST and the double deletion ΔTM12 + TM34 were purified from the soluble fraction after a second centrifugation step by using a similar purification protocol. The purified protein samples were concentrated to ~mg/ml using a centrifugal filter (Amicon Ultra-15 Centricon filter device, 10 kDa, Millipore) along with buffer-exchange to 50 mM HEPES buffer, pH 7.5 with 150 mM NaCl and 0.0075% (w/v) DDM.

### Liposome preparation and co-floatation assay

Liposomes were prepared by the thin film hydration method followed by extrusion using filters. 1,2-dioleoyl-sn-glycero-3-phosphocholine (DOPC, Avanti Polar Lipids Inc.) and 1,2-dioleoyl-sn-glycero-3-phosphoethanolamine (DOPE, Avanti Polar Lipids Inc.) were dissolved in a mixture of chloroform and methanol (4:1) and mixed in a round-bottom flask to obtain the desired molar ratio of 0.8:0.2 (DOPC:DOPE). The organic solvent was then removed by rotary evaporation to obtain a dry lipid film which was then hydrated for 2 h at room temperature with liposome buffer (50 mM HEPES, 150 mM NaCl buffer at pH 7.4) to obtain a final 10 mM lipid solution. Liposomes were formed by constant vortexing followed by sonication in an ultrasound bath. The hydrated liposomes were extruded using a lipid extruder (Avanti Polar Lipids Inc.) and 200 nm polycarbonate membranes (Avanti Polar Lipids). Liposome preparations were used for co-flotation and remodeling experiments.

Liposomes and the purified protein samples were mixed at a 3:1 lipid-to-protein ratio (LPR) and incubated for 2 h at 37 °C in 120 μl of liposome buffer. After incubation, the samples were then mixed with 145 μl of a 60% sucrose solution prepared in liposome buffer to yield a 30% sucrose concentration. The mixture was overlaid with 400 μl of 28% sucrose and 135 μl of liposome buffer. The samples were then centrifuged at 115,000 × *g* for 2 h at 20 °C. After centrifugation, eight fractions (100 μl each) were collected from top to bottom, without disturbing the layers, and analyzed by SDS–PAGE and western blot using an anti-GST rabbit antibody (Cell Signaling Technology, 91G1; #262; dilution 1:2000, see Source Data for original blots).

### Liposome-remodeling assay

Liposomes were mixed with different purified proteins (control GST, FAM134B-RHD, and FAM134B-RHD deletion mutants) at an LPR of 5:1. The liposome–protein mixtures were incubated for 18 h at 22 °C with constant agitation (600 rpm), before imaging by negative-stain electron microscopy (nsEM). The dose-dependence of protein-mediated liposome remodeling behavior was studied by incubating liposomes with increasing protein concentrations (LPR of 40:1, 15:1, and 5:1 for GST, RHD, ΔAH_L_ + AH_C_ and RHD_143–260_). All the samples were examined by negative-stain transmission electron microscopy (nsTEM). For imaging, samples were first diluted with liposome buffer to obtain a final lipid concentration of 1 mg/ml. Carbon-coated copper grids (SPI Supplies) were glow-discharged for 20 s at 15 mA and 0.38 mbar vacuum before sample deposition. 5 μl of each diluted sample was added to the grids. After 1 min incubation, the grids were washed twice with buffer and subsequently stained with 1% uranyl formate for 10 s at room temperature. Excess staining solution was removed by blotting with filter paper. 5–10 micrographs were recorded for each sample using a 120 kV Tecnai Spirit Biotwin electron microscope (FEI) equipped with a 4*k* × 4*k* CCD detector (US4000-1, Gatan). Two different nominal magnifications (×18,500 and ×49,000) with an estimated defocus of ~2–3 μm were used for sample inspection and data acquisition. Image analysis was performed by measuring sizes of proteoliposomes (diameters) formed by various protein–liposome mixtures using ImageJ software. Care was taken to make measurements on round/circular particles (*n* = 300 each) from sampled micrographs collected for each sample (see Source Data).

### Immunofluorescence microscopy

For IF analysis, cells were first grown on glass cover slips for 24 h at 37 °C. After incubation, the cells were transfected with the different plasmids using the Turbofect reagent (Thermo Fisher Scientific). 24 h after transfection, the cells were fixed with 4% (mg/ml) paraformaldehyde for 5 min. Cells were then permeabilized with 0.1% saponin solution in PBS for 5 min and blocked in PBS containing 10% fetal bovine serum (FBS) for 1 h at room temperature. Cells were incubated over night at 4 °C with primary antibody, anti-HA (Roche: #11867423001; RRID: AB_10094468; dilution 1:5000) and anti-calnexin (AbCam: #Ab22595; RRID: AB_2069006; dilution 1:2000), diluted in PBS containing 5% FBS and 0.1% saponin. Secondary antibodies, anti-rabbit Alexa 555 (Life Technologies A31572, Ober-Olm, Germany; dilution 1:500) and anti-rat Alexa 488 (Life Technologies A21208, Ober-Olm, Germany; dilution 1:500) were incubated for 1 h at room temperature. IF images were acquired with the Leica SP8 laser-scanning microscope (Leica). Representative images were obtained from IF experiments carried out at least three times. Selective ER-phagy was quantified by measuring the number of cells with ≥5 ER fragments per cell under each of the conditions (*n* ≥ 100 cells). Data are presented as sample μ ± s.d. (see Source Data). Comparisons of ER-fragmentation in cells expressing wild-type and deletion mutants were performed using parametric two-sampled Student’s *t*-test.

## Supplementary information

Supplementary Information

Supplementary Movie 1

Supplementary Movie 2

Supplementary Movie 3

Supplementary Movie 4

Supplementary Movie 5

Supplementary Movie 6

Supplementary Data 1

Description of Additional Supplementary Files

Source Data

## Data Availability

Data supporting the findings of this manuscript are available from the corresponding authors upon reasonable request. A reporting summary for this Article is available as a Supplementary Information file. The source data underlying Figs. 6a, 6j, 7b, and Supplementary Figs. 20, 24, 25c are provided as a Source Data file.

## Code availability

Custom software used in this work is available from https://github.com/bio-phys/MemCurv.

## References

[1] Schwarz DS, Blower MD (2016). The endoplasmic reticulum: structure, function and response to cellular signaling. Cell. Mol. Life Sci..

[2] Chen S, Novick P, Ferro-Novick S (2013). ER structure and function. Curr. Opin. Cell Biol..

[3] Nixon-Abell J (2016). Increased spatiotemporal resolution reveals highly dynamic dense tubular matrices in the peripheral ER. Science (80-.)..

[4] Friedman JR, Voeltz GK (2011). The ER in 3D: a multifunctional dynamic membrane network. Trends Cell Biol..

[5] Grumati P, Dikic I, Stolz A (2018). ER-phagy at a glance. J. Cell Sci..

[6] Walter P, Ron D (2011). The Unfolded Protein Response: From Stress Pathway to Homeostatic Regulation. Science (80-.)..

[7] Khaminets A (2015). Regulation of endoplasmic reticulum turnover by selective autophagy. Nature.

[8] Fumagalli F (2016). Translocon component Sec62 acts in endoplasmic reticulum turnover during stress recovery. Nat. Cell Biol..

[9] Grumati P (2017). Full length RTN3 regulates turnover of tubular endoplasmic reticulum via selective autophagy. Elife.

[10] Smith M, Wilkinson S (2017). ER homeostasis and autophagy. Essays Biochem..

[11] Chen Q (2019). ATL3 is a tubular ER-phagy receptor for GABARAP-mediated selective autophagy. Curr. Biol..

[12] Stolz A, Ernst A, Dikic I (2014). Cargo recognition and trafficking in selective autophagy. Nat. Cell Biol..

[13] O’Sullivan NC, Jahn TR, Reid E, O’Kane CJ (2012). Reticulon-like-1, the drosophila orthologue of the hereditary spastic paraplegia gene reticulon 2, is required for organization of endoplasmic reticulum and of distal motor axons. Hum. Mol. Genet..

[14] Kurth I (2009). Mutations in FAM134B, encoding a newly identified Golgi protein, cause severe sensory and autonomic neuropathy. Nat. Genet..

[15] Lennemann NJ, Coyne CB (2017). Dengue and Zika viruses subvert reticulophagy by NS2B3-mediated cleavage of FAM134B. Autophagy.

[16] Chiramel AI, Dougherty JD, Nair V, Robertson SJ, Best SM (2016). FAM134B, the selective autophagy receptor for endoplasmic reticulum turnover, inhibits replication of Ebola virus strains Makona and Mayinga. J. Infect. Dis..

[17] Islam Farhadul, Gopalan Vinod, Pillai Suja, Lu Cu-tai, Kasem Kais, Lam Alfred King-yin (2018). Promoter hypermethylation inactivate tumor suppressor FAM134B and is associated with poor prognosis in colorectal cancer. Genes, Chromosomes and Cancer.

[18] Melchiotti, R. et al. Genetic analysis of an allergic rhinitis cohort reveals an intercellular epistasis between FAM134B and CD39. *BMC Med. Genet*. **15**, 73 (2014). 10.1186/1471-2350-15-73 PMC4094447 24970562

[19] Brady JP, Claridge JK, Smith PG, Schnell JR (2015). A conserved amphipathic helix is required for membrane tubule formation by Yop1p. Proc. Natl. Acad. Sci. USA.

[20] Breeze E (2016). A C-terminal amphipathic helix is necessary for the in vivo tubule-shaping function of a plant reticulon. Proc. Natl. Acad. Sci. USA.

[21] Hu J (2008). Membrane proteins of the endoplasmic reticulum induce high-curvature tubules. Science (80-.)..

[22] Voeltz GK, Prinz WA, Shibata Y, Rist JM, Rapoport TA (2006). A class of membrane proteins shaping the tubular endoplasmic reticulum. Cell.

[23] Stansfeld PJ, Sansom MS (2011). Molecular simulation approaches to membrane proteins. Structure.

[24] Hu M, Briguglio JJ, Deserno M (2012). Determining the Gaussian curvature modulus of lipid membranes in simulations. Biophys. J..

[25] Hu M, De Jong DH, Marrink SJ, Deserno M (2013). Gaussian curvature elasticity determined from global shape transformations and local stress distributions: a comparative study using the MARTINI model. Faraday Discuss..

[26] Zurek N, Sparks L, Voeltz G (2011). Reticulon short hairpin transmembrane domains are used to shape ER tubules. Traffic.

[27] Giménez-Andrés M, Čopič A, Antonny B (2018). The many faces of amphipathic helices. Biomolecules.

[28] Boucrot E (2012). Membrane fission is promoted by insertion of amphipathic helices and is restricted by crescent BAR domains. Cell.

[29] Fuhrmans M, Marrink SJ (2012). Molecular view of the role of fusion peptides in promoting positive membrane curvature. J. Am. Chem. Soc..

[30] Cui H (2013). Understanding the role of amphipathic helices in N-bar domain driven membrane remodeling. Biophys. J..

[31] Hofbauer HF (2018). The molecular recognition of phosphatidic acid by an amphipathic helix in Opi1. J. Cell Biol..

[32] Simunovic M, Šarić A, Henderson JM, Lee KYC, Voth GA (2017). Long-range organization of membrane-curving proteins. ACS Cent. Sci..

[33] Strahl H (2015). Transmembrane protein sorting driven by membrane curvature. Nat. Commun..

[34] Tian A, Baumgart T (2009). Sorting of lipids and proteins in membrane curvature gradients. Biophys. J..

[35] Parton DL, Klingelhoefer JW, Sansom MSP (2011). Aggregation of model membrane proteins, modulated by hydrophobic mismatch, membrane curvature, and protein class. Biophys. J..

[36] Fowler PW (2016). Membrane stiffness is modified by integral membrane proteins. Soft Matter.

[37] Aimon S (2014). Membrane shape modulates transmembrane protein distribution. Dev. Cell.

[38] Shibata Y (2008). The reticulon and Dp1/Yop1p proteins form immobile oligomers in the tubular endoplasmic reticulum. J. Biol. Chem..

[39] Forrester Alison, De Leonibus Chiara, Grumati Paolo, Fasana Elisa, Piemontese Marilina, Staiano Leopoldo, Fregno Ilaria, Raimondi Andrea, Marazza Alessandro, Bruno Gemma, Iavazzo Maria, Intartaglia Daniela, Seczynska Marta, van Anken Eelco, Conte Ivan, De Matteis Maria Antonietta, Dikic Ivan, Molinari Maurizio, Settembre Carmine (2018). A selective ER‐phagy exerts procollagen quality control via a Calnexin‐FAM134B complex. The EMBO Journal.

[40] Mochida K (2015). Receptor-mediated selective autophagy degrades the endoplasmic reticulum and the nucleus. Nature.

[41] Beale R (2014). A LC3-interacting motif in the influenza A virus M2 protein is required to subvert autophagy and maintain virion stability. Cell Host Microbe.

[42] Finn RD (2016). The Pfam protein families database: towards a more sustainable future. Nucleic Acids Res..

[43] Marchler-Bauer A (2017). CDD/SPARCLE: functional classification of proteins via subfamily domain architectures. Nucleic Acids Res..

[44] Altschul SF (1997). Gapped BLAST and PSI-BLAST: a new generation of protein database search programs. Nucleic Acids Res..

[45] Li W, Godzik A (2006). Cd-hit: a fast program for clustering and comparing large sets of protein or nucleotide sequences. Bioinformatics.

[46] Katoh K, Standley DM (2013). MAFFT multiple sequence alignment software version 7: improvements in performance and usability. Mol. Biol. Evol..

[47] Pupko T, Bell RE, Mayrose I, Glaser F, Ben-Tal N (2002). Rate4Site: an algorithmic tool for the identification of functional regions in proteins by surface mapping of evolutionary determinants within their homologues. Bioinformatics.

[48] Stamm M, Staritzbichler R, Khafizov K, Forrest LR (2014). AlignMe—a membrane protein sequence alignment web server. Nucleic Acids Res..

[49] Tsirigos KD, Peters C, Shu N, Käll L, Elofsson A (2015). The TOPCONS web server for consensus prediction of membrane protein topology and signal peptides. Nucleic Acids Res..

[50] Jones DDTD (1999). Protein secondary structure prediction based on position-specific scoring matrices. J. Mol. Biol..

[51] Meruelo AD, Samish I, Bowie JU (2011). TMKink: a method to predict transmembrane helix kinks. Protein Sci..

[52] Gautier R, Douguet D, Antonny B, Drin G (2008). HELIQUEST: a web server to screen sequences with specific α-helical properties. Bioinformatics.

[53] Källberg M (2012). Template-based protein structure modeling using the RaptorX web server. Nat. Protoc..

[54] Šali A, Blundell TL (1993). Comparative protein modelling by satisfaction of spatial restraints. J. Mol. Biol..

[55] Lomize MA, Pogozheva ID, Joo H, Mosberg HI, Lomize AL (2012). OPM database and PPM web server: resources for positioning of proteins in membranes. Nucleic Acids Res..

[56] Jo S, Kim T, Iyer VG, Im W (2008). CHARMM-GUI: a web-based graphical user interface for CHARMM. J. Comput. Chem..

[57] Jo S, Lim JB, Klauda JB, Im W (2009). CHARMM-GUI membrane builder for mixed bilayers and its application to yeast membranes. Biophys. J..

[58] Huang J (2016). CHARMM36m: an improved force field for folded and intrinsically disordered proteins. Nat. Methods.

[59] Nosé S (1984). A molecular dynamics method for simulations in the canonical ensemble. Mol. Phys..

[60] Nosé S (1984). A unified formulation of the constant temperature molecular dynamics methods. J. Chem. Phys..

[61] Hoover WG (1985). Canonical dynamics: equilibrium phase-space distributions. Phys. Rev. A.

[62] Parrinello M, Rahman A (1981). Polymorphic transitions in single crystals: a new molecular dynamics method. J. Appl. Phys..

[63] Marrink SJ, Risselada HJ, Yefimov S, Tieleman DP, De Vries AH (2007). The MARTINI force field: coarse grained model for biomolecular simulations. J. Phys. Chem. B.

[64] Monticelli L (2008). The MARTINI coarse-grained force field: extension to proteins. J. Chem. Theory Comput..

[65] Kabsch W, Sander C (1983). Dictionary of protein secondary structure: pattern recognition of hydrogen-bonded and geometrical features. Biopolymers.

[66] Wassenaar Ta, Ingólfsson HI, Böckmann Ra, Tieleman DP, Marrink SJ (2015). Computational lipidomics with insane: a versatile tool for generating custom membranes for molecular simulations. J. Chem. Theory Comput..

[67] Berendsen HJC, Postma JPM, Gunsteren WF, van, DiNola A, Haak JR (1984). Molecular dynamics with coupling to an external bath. J. Chem. Phys..

[68] Bussi G, Donadio D, Parrinello M (2007). Canonical sampling through velocity rescaling. J. Chem. Phys..

[69] Pronk S (2013). GROMACS 4.5: a high-throughput and highly parallel open source molecular simulation toolkit. Bioinformatics.

