# Supplementary material for: Curvature induction and membrane remodeling by FAM134B reticulon homology domain assist selective ER-phagy
Source: Nat Commun. 2019 May 30;10:2370. doi: 10.1038/s41467-019-10345-3 (PMC6542808; doi:10.1038/s41467-019-10345-3)
Supplement: Supplementary file 1 — Supplementary Information [file 41467_2019_10345_MOESM1_ESM.pdf]

Supplementary information for: Curvature induction and membrane remodeling by FAM134B reticulon homology domain assist selective ER-phagy

Ramachandra M. Bhaskara,[1] Paolo Grumati,[2] Javier Garcia-Pardo,[3,4] Sissy Kalayil,[3] Adriana Covarrubias-Pinto,[2] Wenbo Chen,[5,3] Mikhail Kudryashev,[5,3] Ivan Dikic,[2,3,a)] and Gerhard Hummer[1,6,b)]

[1)] *Department of Theoretical Biophysics, Max Planck Institute of Biophysics, Max-von-Laue Straße 3, 60438 Frankfurt am Main, Germany*
[2)] *Institute of Biochemistry II, School of Medicine, Goethe University Frankfurt, Theoder-Stern-Kai 7, 60590 Frankfurt am Main, Germany*
[3)] *Buchmann Institute of Molecular Life Sciences, Goethe University Frankfurt, Max-von-Laue Straße 15, 60438, Frankfurt am Main, Germany*
[4)] *Fraunhofer Institute for Molecular Biology and Applied Ecology, Division for Translational Medicine and Pharmacology, Theodor-Stern-Kai 7, 60596 Frankfurt am Main*
[5)] *Max Planck Institute of Biophysics, Max-von-Laue Straße 3, 60438 Frankfurt am Main, Germany*
[6)] *Institute of Biophysics, Goethe University Frankfurt, 60438 Frankfurt am Main, Germany*

Keywords: FAM134B; ER remodeling; Membrane Curvature; Autophagy; Vesiculation

---

This document contains the following sections:

1. **Supplementary Figures** (1-27)
2. **Supplementary Tables** (1-6)
3. **Supplementary Methods**
4. **Supplementary Notes** (1-8)
5. **Supplementary References**

[a)] I.D. email: dikic@biochem2.uni-frankfurt.de
[b)] G.H. email: gerhard.hummer@biophys.mpg.de

SUPPLEMENTARY FIGURES

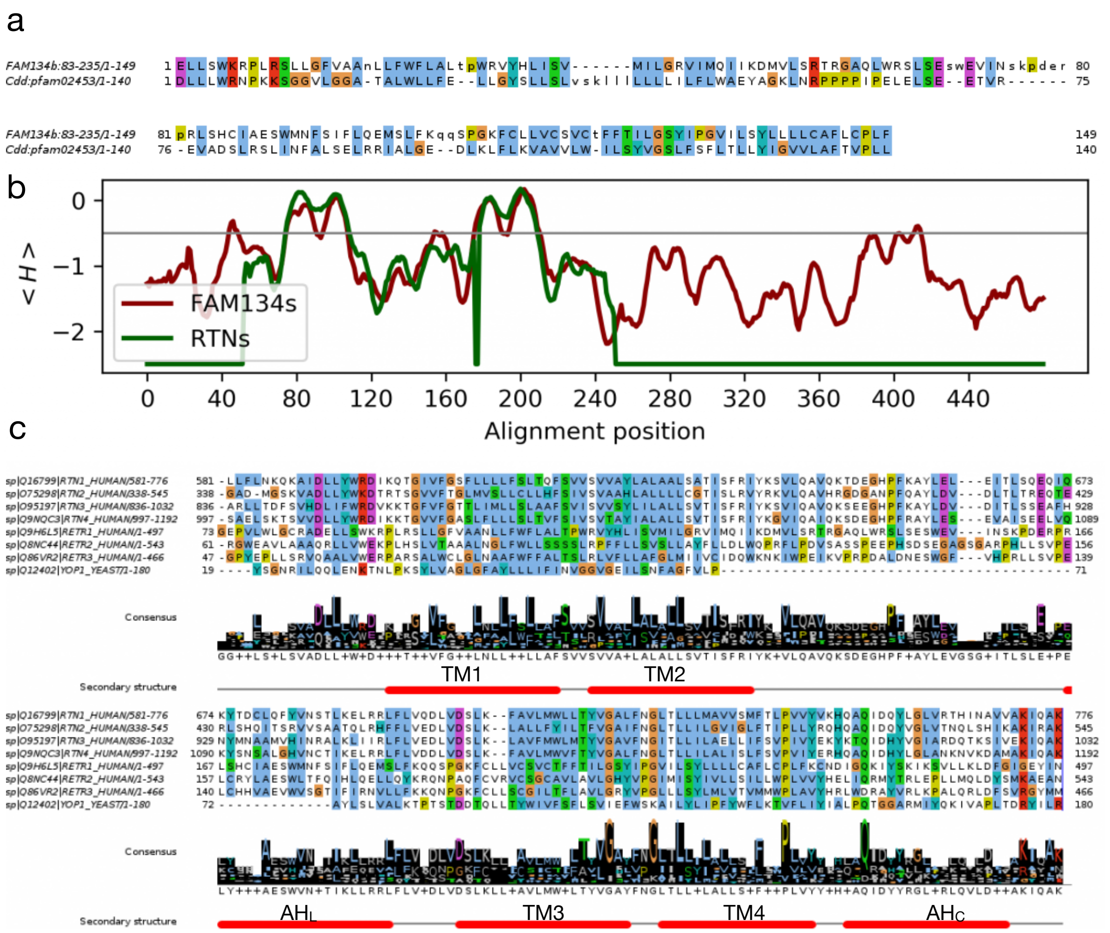

Supplementary Figure 1. **RHD signatures in FAM134B. a** Alignment of FAM134B sequence (83-235) to the RHD profile (PF02453) enabled domain annotation. **b** Alignment of hydrophobicity profiles show high degree of overlap between FAM134B homologs (red) and RTN-4 homologs (green). Consensus hydrophobicity profiles of FAM134B and RTN-4 family, computed by averaging hydrophobicity profiles over a 15-residue running window were used to align the profiles using AlignME[1]. **c** Selected human sequences from this profile-profile alignment highlighting topologically equivalent sites of the RHDs in FAM134 sequences (renamed RETR1-3) and RTNs. The alignment also includes yeast YOP1P (aligned to RETR1 using AlignME[1]). Alignments in **a** and **c** were colored according to the ClustalX scheme[2] and rendered using Jalview[3].

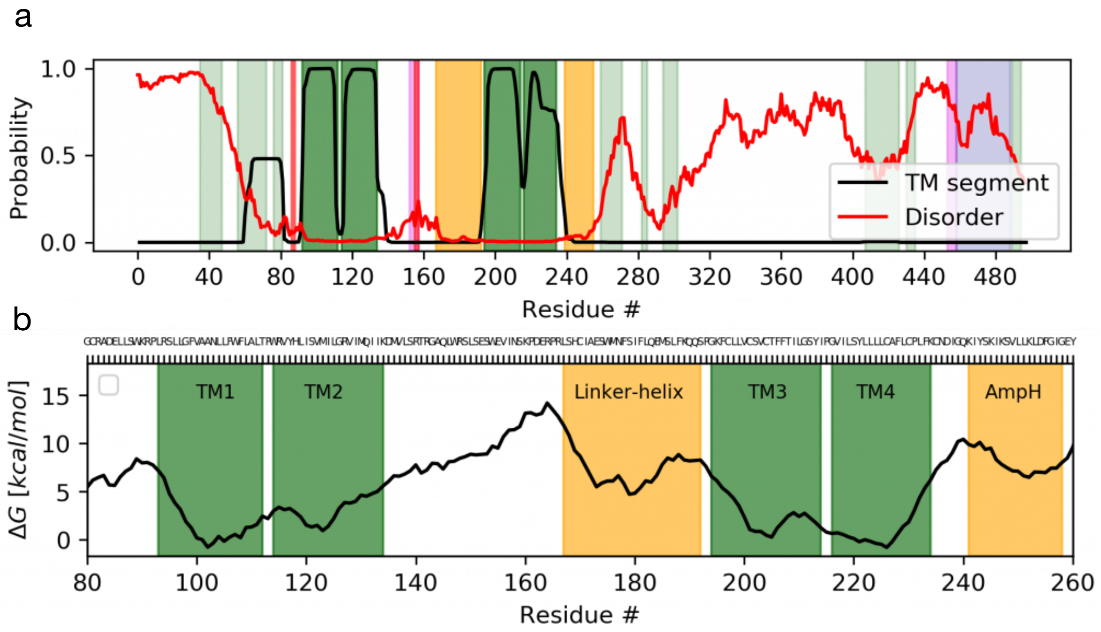

Supplementary Figure 2. **Annotation of FAM134B sequence.** (**a**) Mapping of multiple sequence-based assignments to FAM134B sequence. Helix (shaded green), strand (shaded red) assignments were derived from consensus PSI-pred and JNetPred[4]. TM region (dark green) and protein membrane topology (black line) were annotated using TopCons[5]. Amphipathic helices (shaded yellow) showing maximal hydrophobic moment were derived from Heliquest[6]. Disordered regions (red line) in FAM134B sequence were annotated using Metadisopred[7]. Predicted LIR motifs (magenta) and coiled-coil region (blue) were obtained from iLiR[8] and MARCOIL[9], respectively. (**b**) Zoom-in on the structured region (80-260) of FAM134B, encompassing the assigned RHD (83-235), showing sequence based prediction of local membrane-interaction strength (black line)[10].

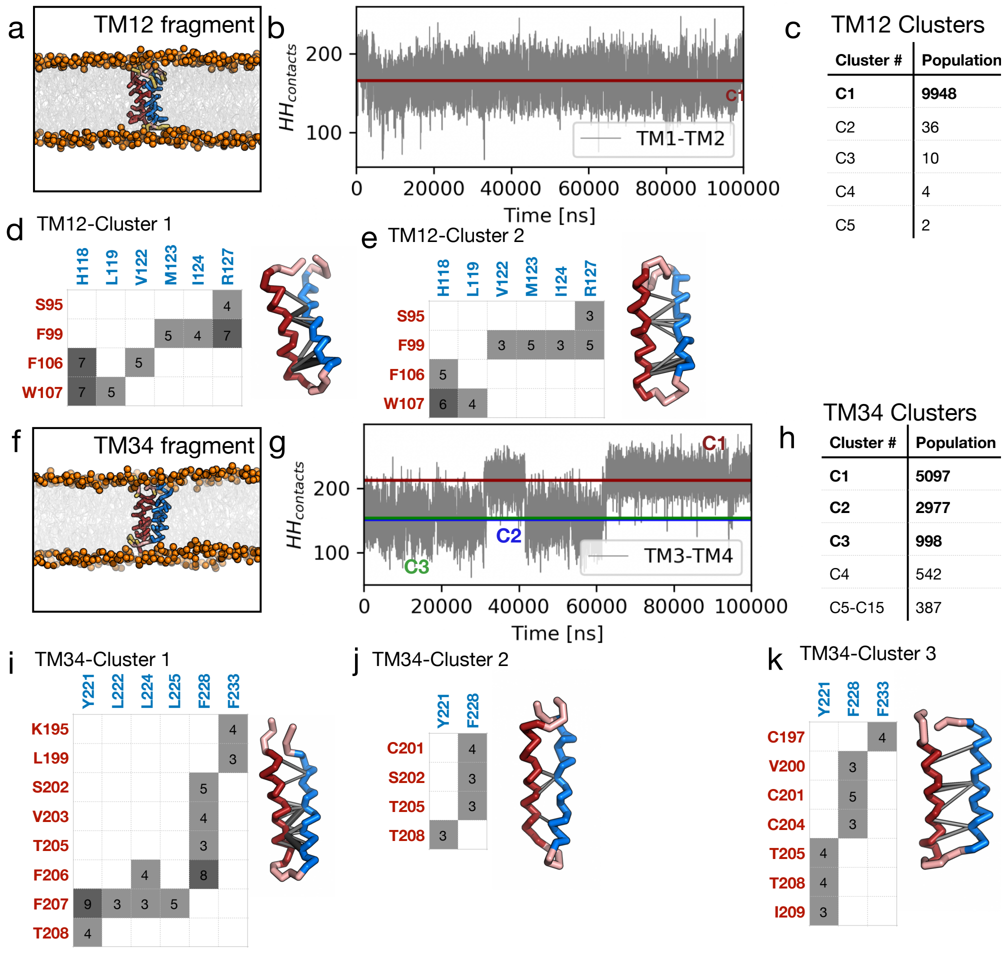

Supplementary Figure 3. **Conformational states of transmembrane hairpins.** Discrete well populated conformations of TM fragments were obtained from extensive conformational sampling (100 μs) and clustering using MD simulations of CG representations. (**a**) A representative snapshot from the CG simulation of the TM12 fragment. The hairpin structure of the TM12 fragment is stabilized by tertiary contacts between TM1 (red) and TM2 (blue) in the POPC bilayer (orange phosphate groups). (**b**) The total number of inter-helical contacts ($HH_{contacts}$) quantifies the extent of helix-helix interactions in sampled conformations. (c) Clustering of 10000 structures (using a RMSD cut-off of 3.0 Å), sampled at equal time intervals from the TM12-trajectory, resulted in a single large cluster (TM12-cluster-1). (**d-e**) Average inter-helical, pair-wise residue contact matrix highlighting strong interactions ($\geq$ 3 contacts; see Methods) and network of helix-helix interactions characterizing clusters 1 and 2 of the TM12 fragment (edges colored and scaled according to number of contacts). (**f**) A snapshot of the TM34 fragment simulation showing tertiary contacts between TM3 (red) and TM4 (blue) in a POPC bilayer. (**g**) The total number of inter-helix contacts along the trajectory shows two clear populations corresponding to clusters 1 (red line) and 2+3 combined, respectively. (**h**) 15 clusters, with the top three clusters accounting for 90.7 % of the sampled conformations, were obtained for TM34-fragment. (**i-k**) Contact matrix and helix-helix interaction networks characterizing clusters 1, 2, and 3 of the TM34 fragment (same as **d-e**). Numbers indicate $\geq$ 3 contacts across a pair of residues.

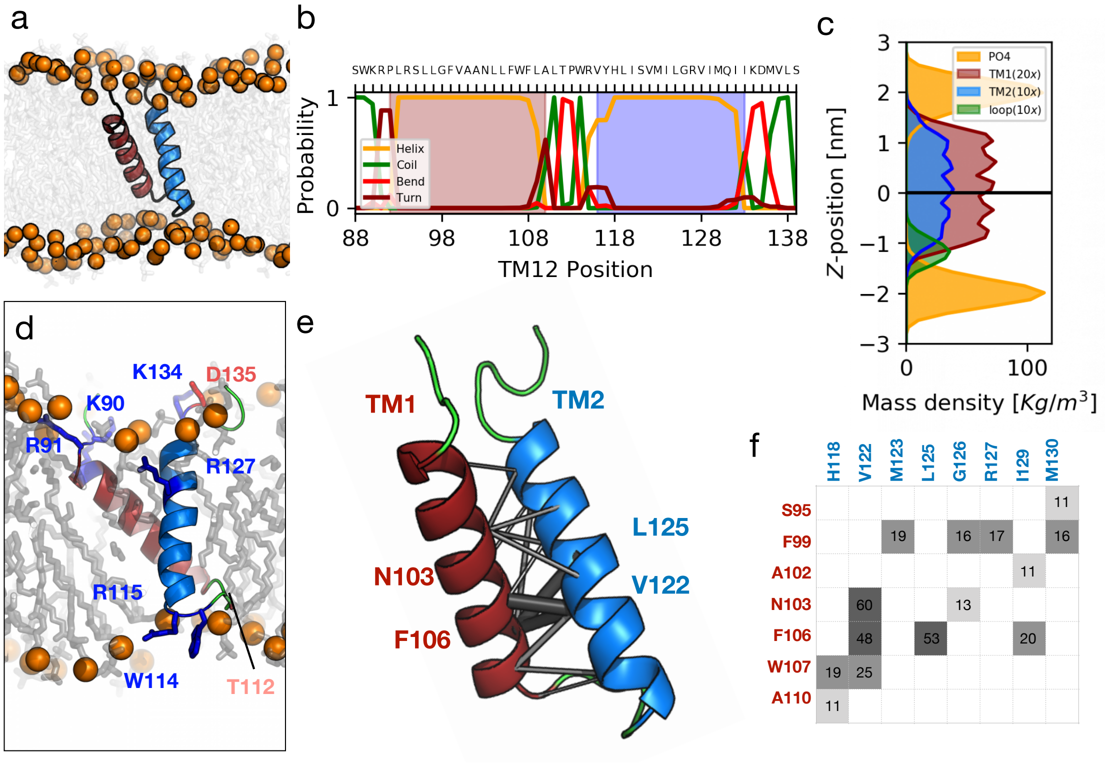

Supplementary Figure 4. **Structure of FAM134B-TM12 fragment.** (**a**) Representative snapshot of TM12 fragment structure, obtained from all-atom MD simulation (>3.0 μ s). A representative CG structure of TM12 cluster 1 was back-mapped and refined using all-atom MD simulations (Supplementary Fig. 3d). (**b**) Secondary structure distribution derived from all-atom MD simulations provides an assessment of local conformational variability in the hairpin structure. (**c**) C_α density profiles of TM1 (red), TM2 (blue) and the connecting loop (green), for clarity magnified 20x, 10x, and 10x, respectively, show their equilibrium position relative to the bilayer normal (orange, phosphate groups). The TM12 fragment assumes a tilted conformation with respect to the membrane normal, ($\theta = 16 \pm 7°$). The two interacting helices cross at an angle of 52°. (**d**) Charged/polar residues (labeled) anchor the TM12 fragment at both leaflets and stabilize its position and orientation in the membrane. (**e**) Network of helix-helix interactions stabilizing the hairpin structure (edges scaled and colored by number of contacts). (**f**) Pair-wise residue contact matrix highlighting important interactions ($\geq$ 10 heavy atom contacts; see Supplementary Methods).

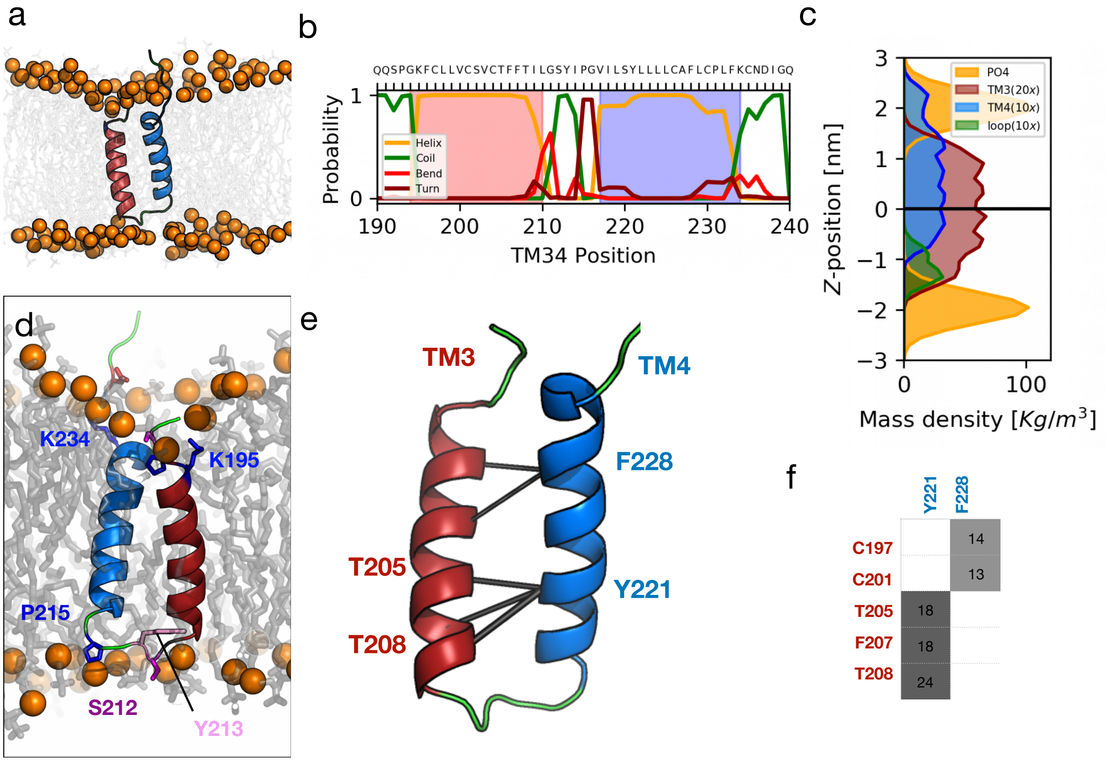

Supplementary Figure 5. **Structure of FAM134B-TM34 fragment.** (**a**) Refined structure of hairpin TM34 obtained from all-atom MD simulation (>3.0 μs) of back-mapped representative of CG cluster 3 (Supplementary Fig. 3k). (**b**) Secondary structure distribution obtained during the all-atom MD simulations quantifies local conformational variability in the hairpin structure. (**c**) C_α density profiles of TM3 (red), TM4 (blue), and the connecting loop (green), for clarity magnified 20x, 10x, and 10x, respectively, show their equilibrium position relative to the bilayer normal (orange, phosphate groups). The TM34 fragment assumes a tilted conformation with respect to the membrane normal ($\theta = 10 \pm 7°$). The two interacting helices cross at an angle of 50°. (**d**) Charged and polar residues anchor the TM34 fragment at both leaflets and stabilize its position and orientation in the membrane. (**e**) Network of helix-helix interactions stabilizing the hairpin structure (edges scaled and colored by number of contacts). (**f**) Pair-wise residue contact matrix highlighting key inter-helical interactions (>10 heavy atom contacts; see Supplementary Methods).

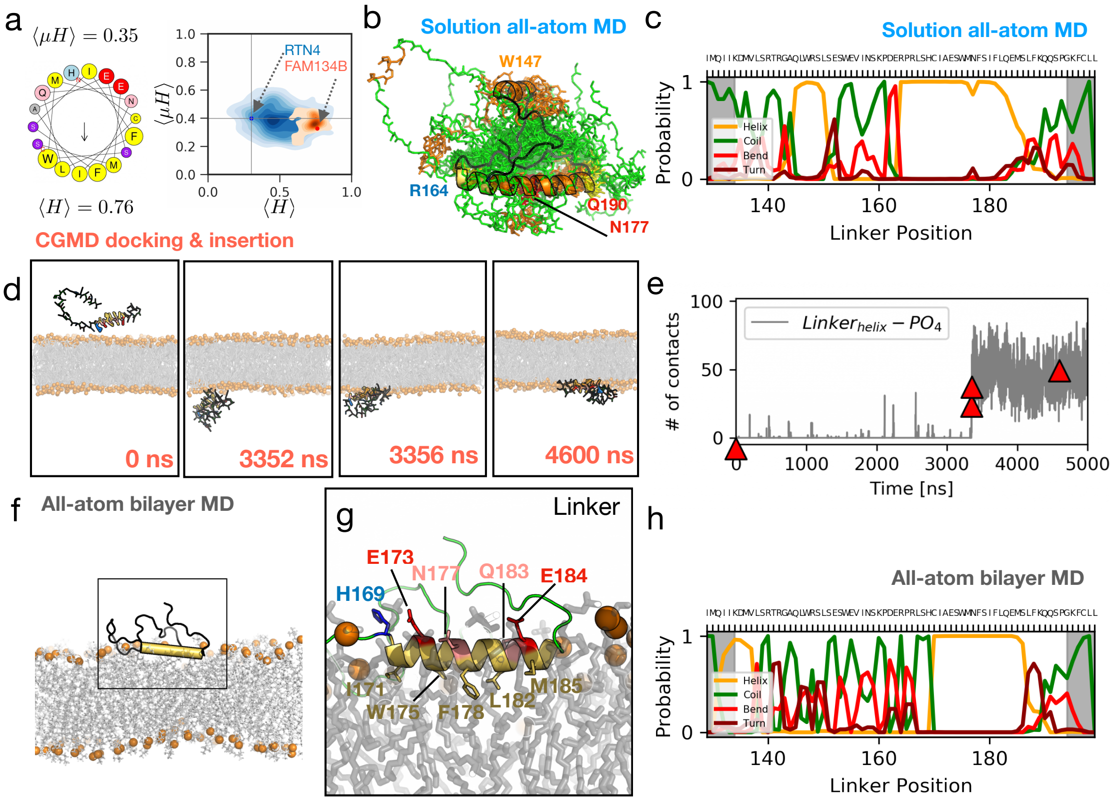

Supplementary Figure 6. **FAM134B-RHD AH$_L$ is amphipathic.** (**a**) Helical wheel representation of the designated helical stretch in FAM134B-linker fragment (AH$_L$; 164-188). Joint $\langle H\rangle$-$\langle\mu_H\rangle$ distributions computed from topologically equivalent helical regions (extracted from alignments) of proteins from FAM134 family (n=134; orange) and RTN4 family (n=500; blue). (**b**) 40 conformations sampled from all-atom solution MD simulations of FAM134B-linker fragment (129-195), superimposed after least squares fit to the helical region (164-188; yellow-orange). (**c**) Secondary structure distributions from all-atom solution simulations show that the AH$_L$ has a predominant kink at N177. (**d**) Selected snapshots from CG simulation (red triangles, right) showing intermediates along docking and insertion of AH$_L$ onto PC-rich bilayer. The predicted non-polar face of the AH$_L$ (yellow/ orange) interacts with lipid acyl chains. The charged and polar face of the AH$_L$ (blue/ red/ magenta) interacts with the head groups (orange spheres, phosphate groups) and water (not shown). (**e**) Steps in the number of contacts between AH$_L$ residues and lipid head groups indicate helix-to-membrane docking attempts. Red triangles correspond to the intermediate structures in **d**. (**f**) Snapshot of the membrane-bound linker fragment from all-atom MD simulation after insertion of AH$_L$ (indicated by yellow cylinder). (**g**) Zoom-in on **f**, showing the detailed structure of the AH$_L$ at the bilayer-water interface, and highlighting predicted non-polar residue-bilayer interactions observed in all-atom MD simulations. (**h**) Secondary structure distribution from all-atom simulations show the stabilization of the AH$_L$.

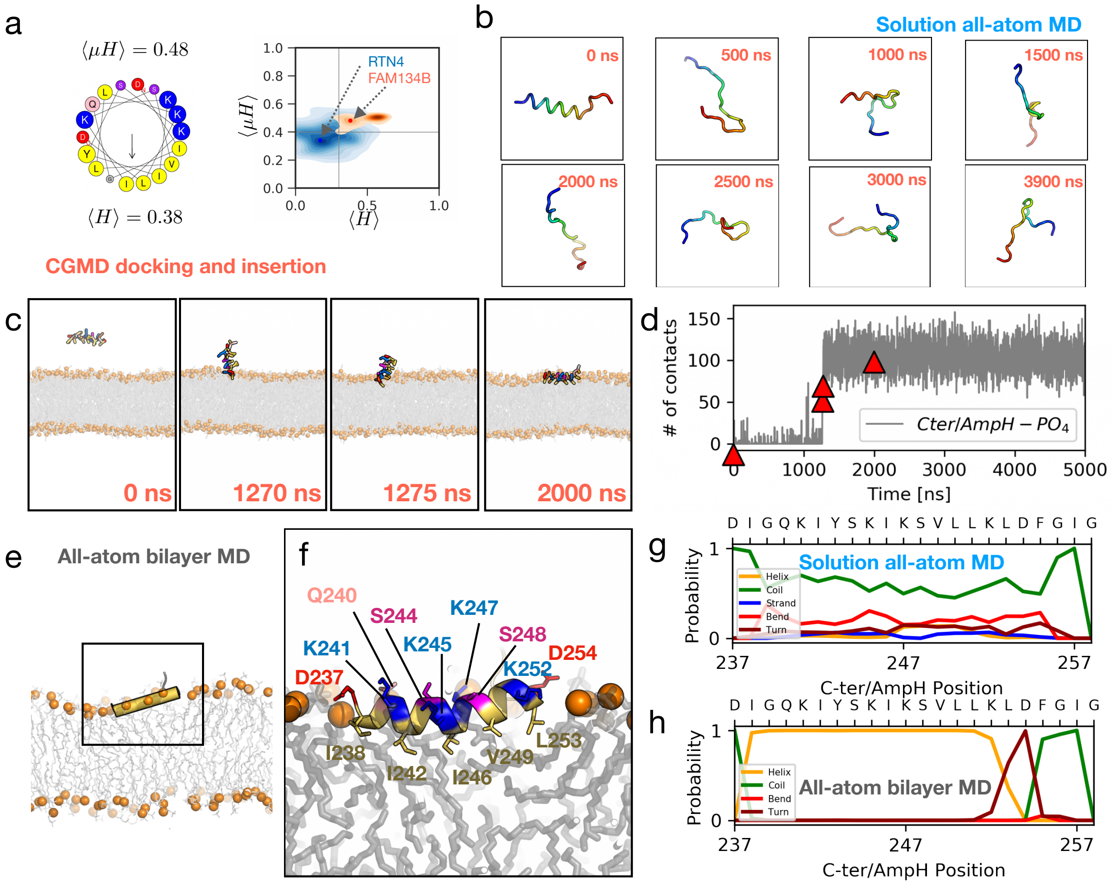

Supplementary Figure 7. **AH$_C$ is amphipathic and resembles canonical RTNs.** (**a**) Helical wheel representation of the C-terminal sequence stretch (AH$_C$) with maximal hydrophobic moment. Joint $\langle H \rangle$-$\langle \mu_H \rangle$ distributions computed from topologically equivalent helices (extracted from alignments) of proteins from FAM134 family (n=134; orange) and RTN4 family (n=500; blue). (**b**) Snapshots spanning the all-atom MD simulation of the AH$_C$ in solution. AH$_C$ unfolds in solution under physiological conditions. (**c**) Selected snapshots (red triangles) showing docking and insertion of the AH$_C$ in CG simulations. Non-polar AH$_C$ residues (yellow/ orange) interact with acyl groups while the charged/polar (blue/ red/ magenta) residues interact with the head groups (orange spheres, phosphate beads) and water (not shown) after insertion. (**d**) Increase in the number of contacts between AH$_C$ residues and lipid head groups captures docking attempts and key intermediates along insertion event (red triangles). (**e**) Snapshot of membrane bound AH$_C$ from all-atom MD simulations. Specific membrane interactions are required for AH$_C$ stabilization. (**f**) Zoom-in on **e** showing the detailed structure and membrane interactions of AH$_C$ residues observed in all-atom MD simulations. (**g-h**) Secondary structure distributions from all-atom MD simulations indicate predominantly coil-like conformations in solution (**g**). Helical conformation is stabilized only in the presence of lipid bilayer (**h**).

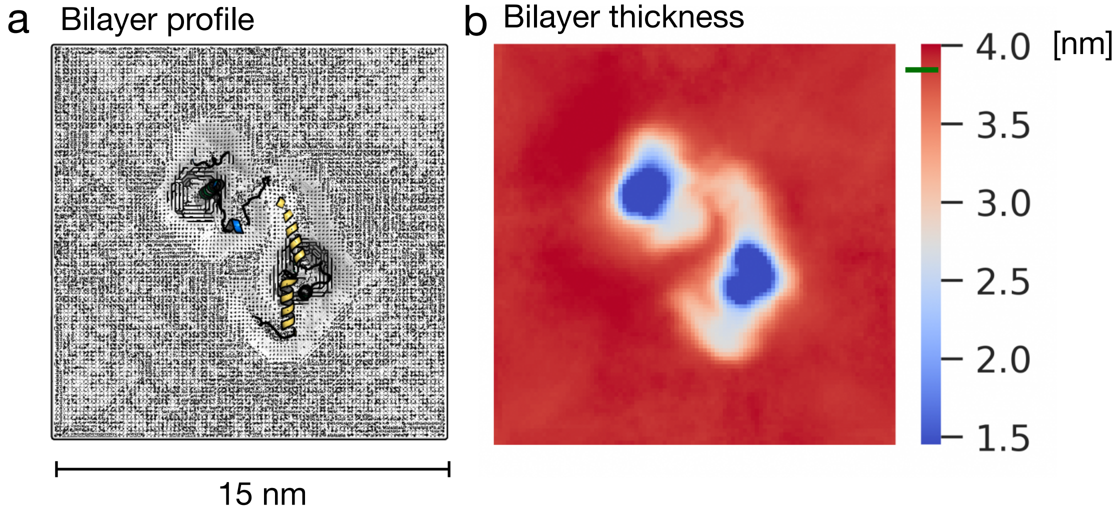

Supplementary Figure 8. **Effect of FAM134B-RHD on local membrane properties.** Local membrane properties are computed from all-atom MD simulations of FAM134B-RHD embedded in POPC bilayer. 100 bins were used to span the x- and y-axis of the 15×15 nm^2 periodic box. Time-averaged lipid positions were mapped locally to a grid and used to compute local membrane properties (heatmaps) using *g_ lomep* tool[11]. (**a**) Average bilayer profile (grey mesh, phosphate positions; top view) showing the asymmetric insertion of FAM134B-RHD (colored) in the ER membrane. (**b**) Time-averaged local bilayer thickness shows local compression of the bilayer in the vicinity of TM hairpins. Distribution of lipids around FAM134B-RHD is sparse on the cytoplasmic leaflet in comparison to the luminal leaflet. Expected values of bilayer thickness for POPC bilayer are marked (green line).

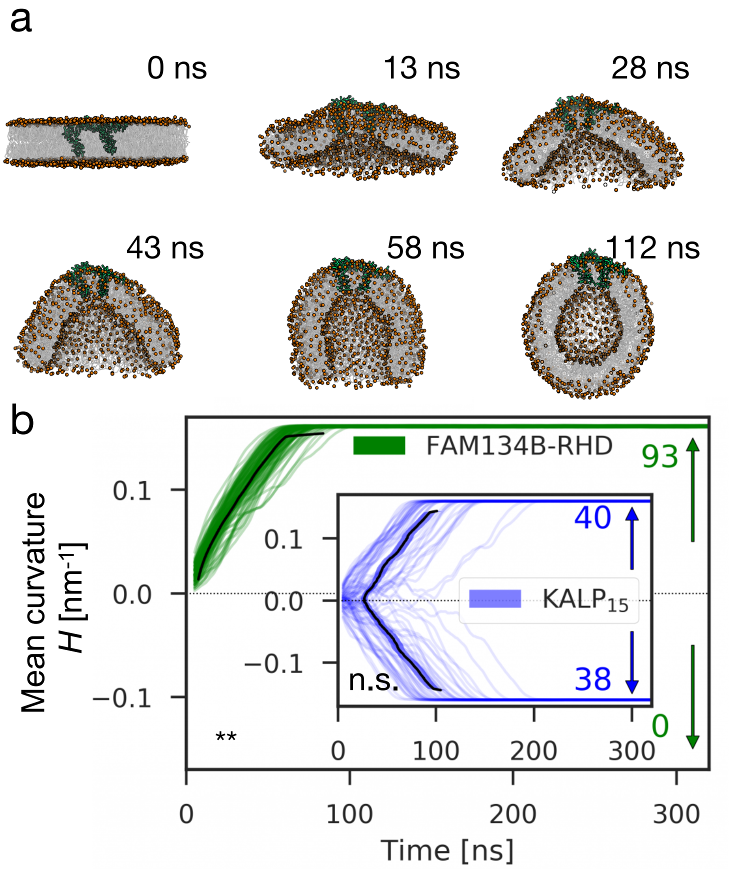

Supplementary Figure 9. **In silico curvature induction assay.** Stochastic shape transformations of open discontinuous bilayer patches (34:1 PC) in aqueous solution close the initially circular discs into vesicles. The presence of curvature-inducing proteins breaks the bilayer symmetry and affects the direction of membrane curvature ($\pm H$) and the rate of vesicle formation. (**a**) Snapshots showing intermediate structures of bilayer patches (phosphate spheres, orange; grey acyl tail groups) containing FAM134B (green) along a vesiculation pathway. (**b**) Bilayer curvature changes ($\pm H$) as a function of simulation time monitor the shape transformations observed in MD simulations en route to vesicle formation. FAM134B (green curves) actively curves bilayers away from the cytosolic/upper leaflet (green arrows), inducing fast vesiculation in repeated runs (93/93). (Inset) $KALP_{15}$ does not alter vesicle formation rates compared to empty bilayer patches (no protein; see Supplementary Table 5) and bilayer closure events are observed along both leaflets (blue arrows) with equal probability (40/78). Individual curvature-time traces computed from 100 replicates were smoothed (running averages over 11 ns windows; colored lines) and averaged to obtain mean (black line) trace ± s.e.m. (shaded region). n.s and * denote one-tailed p-values in binomial tests.

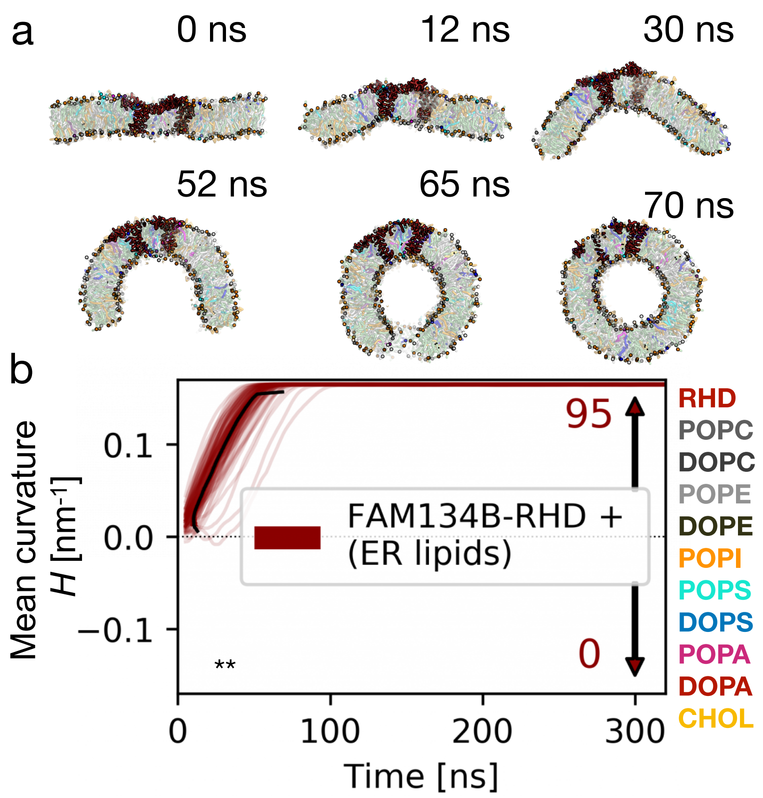

Supplementary Figure 10. **Effect of ER lipids on in silico curvature induction.** Stochastic shape transformations of open discontinuous bilayer patches (made of ER lipids) in solvent result in circular discs and subsequently closed vesicles. (**a**) Snapshots showing intermediate structures of FAM134B (dark red) containing ER membrane patch along a vesiculation pathway. (**b**) Bilayer curvature changes ($\pm H$) monitor the shape transformations observed in MD simulations, leading to vesicle formation. FAM134B (dark red curves) actively curves bilayers away from the cytosolic/upper leaflet (red arrows) inducing faster vesiculation in repeated runs (95/95). Individual curvature-time traces computed from 100 replicates were smoothed (running averages over 11 ns windows; colored lines) and averaged to obtain mean (black line) trace ± s.e.m. (shaded region). n.s and * denote one-tailed p-values in binomial tests.

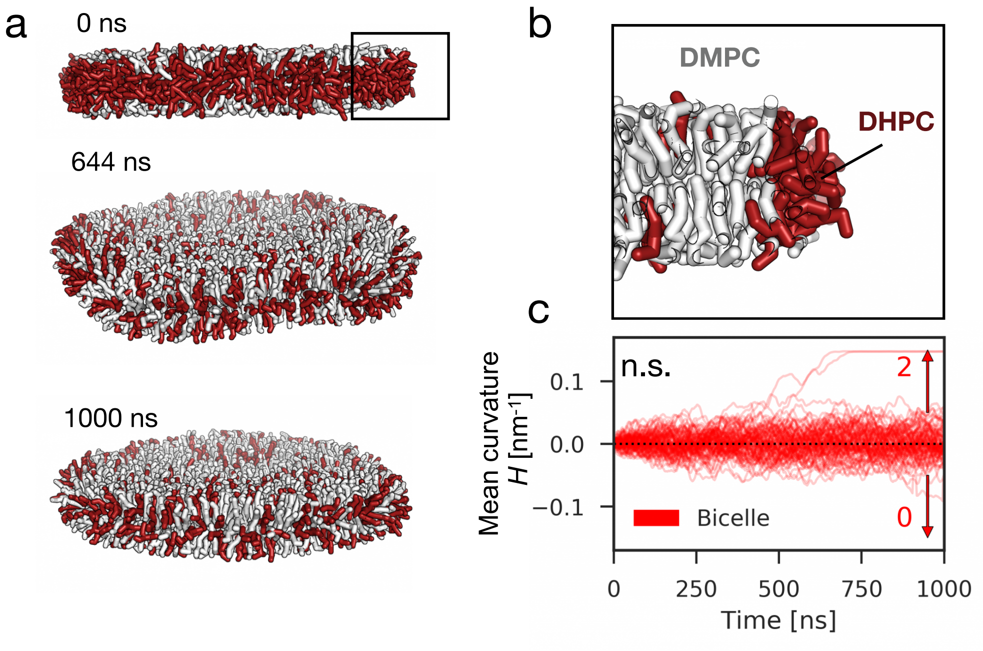

Supplementary Figure 11. **Bicelle-to-vesicle transitions.** (**a**) Snapshots from coarse-grained MD simulations, displaying intermediate bicelle structures. (**b**) Close-up view of cross section of the initial bicelle structure. Short chain DHPC lipids (red) specifically organize at the rim of the predominantly DMPC bilayer (grey) disc, stabilizing the otherwise unstable edge. (**c**) Curvature as a function of time (red lines, smoothed running averages over 11 ns widows) from individual replicates quantify the bicelle shape changes during simulations. The bicelle disc remains relatively flat ($H = \pm 0.05$ nm^{-1}), rarely displaying vesiculation events (2 out of 95 runs) within the simulation time scale (1 μs each). The numbers of vesiculation events up and down (listed) are not significantly different (n.s. denotes the one-tailed probability in binomial tests for bias in the directionality of vesiculation).

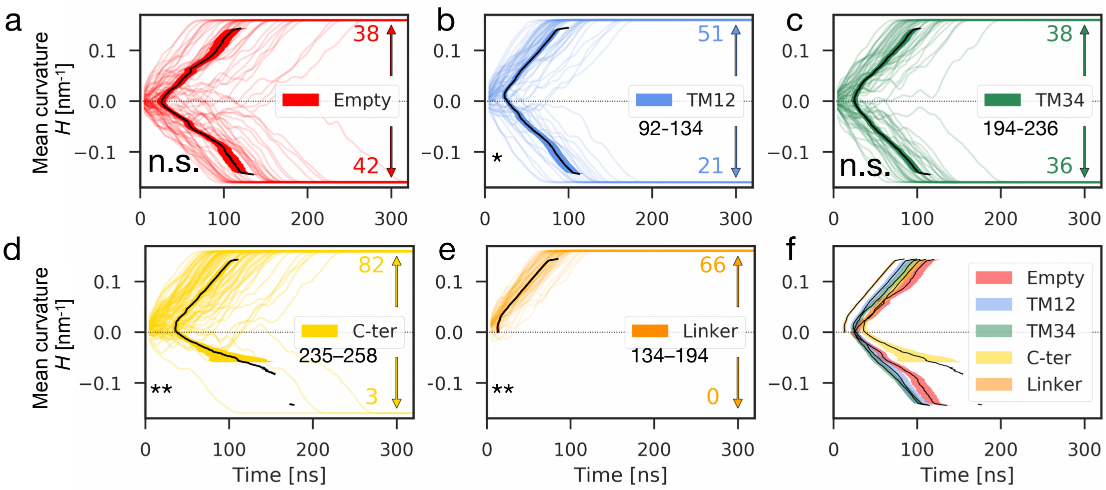

Supplementary Figure 12. **Effect of individual FAM134B-fragments on vesiculation.** (**a**) Empty bilayers. (**b-f**) Bilayers with (**b**) TM12, (**c**) TM34, (**d**) C-terminal segment containing AH_C, (**e**) linker segment containing AH_L. The time-dependent bilayer curvature changes in $n = 100$ independent trajectories each are shown as smoothed, colored lines. Black lines indicate the average. Numbers indicate the direction of the successful vesiculation events, which place the cytosolic side of the RHD fragments outside (n_+; top) or inside the vesicles (n_-; bottom). See Supplementary Figs. 13-14 and Supplementary Tables 4, 5. (**f**) Comparison of mean ± s.e.m curvature-time trace (black lines ± shaded regions) for each fragment. The peripheral binding linker fragment (colored orange) induces fast, highly directional vesiculation. Fragments containing amphipathic helices (Linker and C-ter) also show strong bias in direction of curvature induction (upper/cytosolic leaflet $p < 0.05$; binomial tests). See also Supplementary Fig. 26 for genetic variant FAM134B-Q145X and proteolytic cleavage, which disrupt the RHD and produce fragments resembling TM12 and do not show accelerated vesiculation and directional bias in curvature induction.

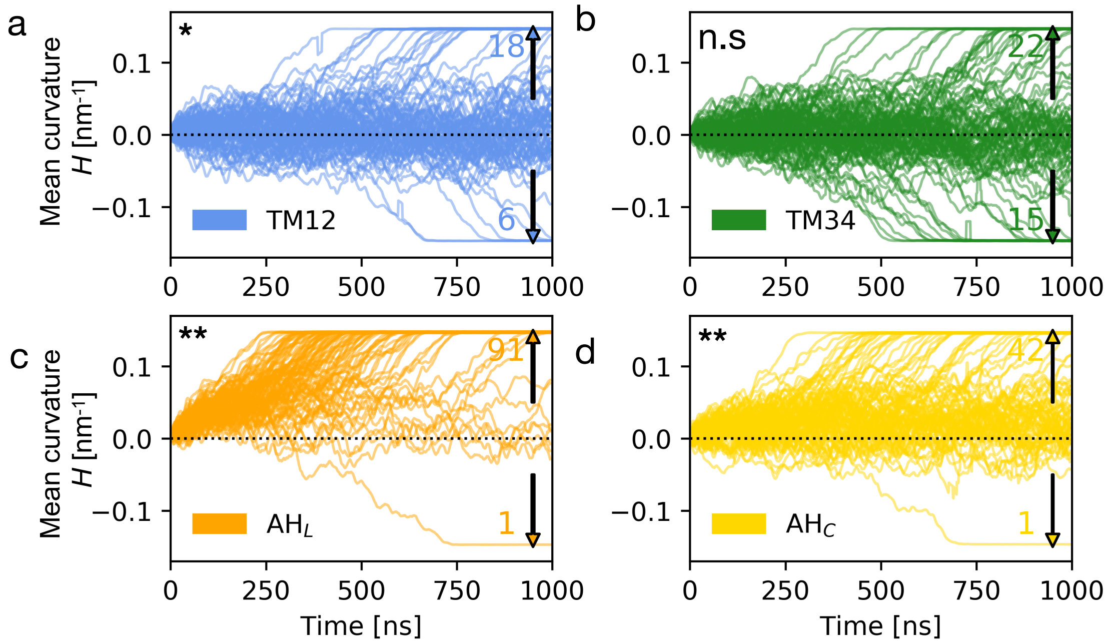

Supplementary Figure 13. **Effect of individual FAM134B-fragments on bicelle-to-vesicle transitions.** Curvature induction capacity of TM hairpins (**a-b**) and amphipathic helix containing (**c-d**) fragments of the RHD tested using bicelle-to-vesicle simulations. 100 replicates of each simulation were performed to assess the directionality and speed of vesicle formation. Linker fragment induced vesiculation events efficiently, 92/96 within the simulation time. Curvature time-traces, smoothed running averages over 11 ns widows) from individual replicates quantify the bicelle shape transformation process during simulations. ** and n.s. denote the one-tailed probability in binomial tests for bias in number of vesiculation events with positive and negative bicelle curvature.

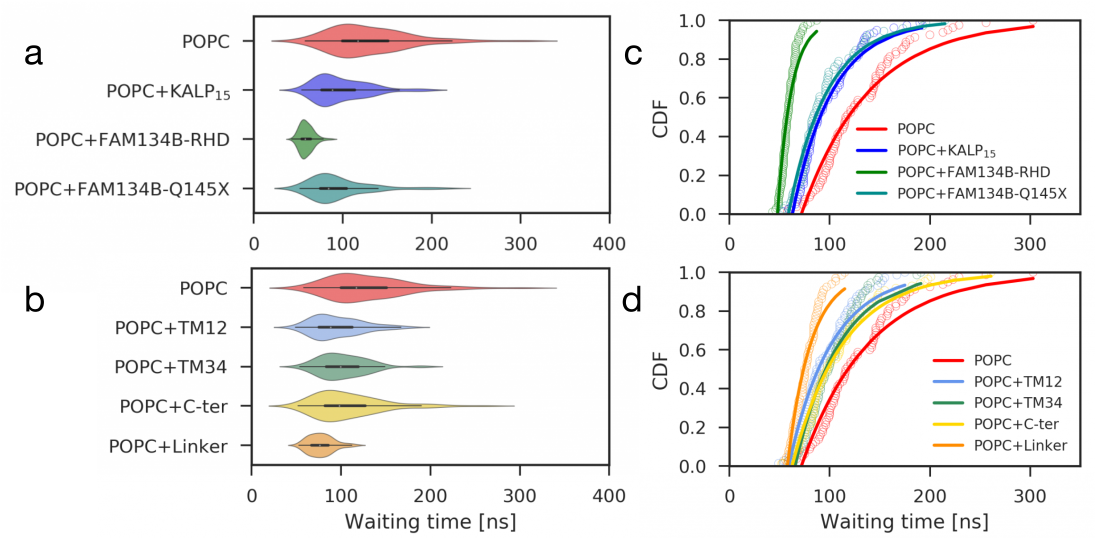

Supplementary Figure 14. **Kinetics of bilayer-to-vesicle transitions.** (**a**) Violin plots showing the distribution of total waiting times for bilayer-to-vesicle transitions for empty bilayers (POPC; red), bilayers with $KALP_{15}$ (blue), intact FAM134B-RHD (green), and FAM134B-Q145X (dark cyan). (**b**) Waiting-time distributions for the individual membrane interacting fragments of FAM134B-RHD. (**c-d**) Cumulative distribution functions of recorded waiting times for formation of vesicle (open circles: simulations; lines: single Poisson process with additional lag time). In the analysis, we considered the vesicle formation as completed when the bilayer-disc curvature exceeded a value of $|H| = 0.15\ \mathrm{nm}^{-1}$. Rate constants and acceleration factors for vesicle formation were extracted from fits of Poisson distributions with lag times.

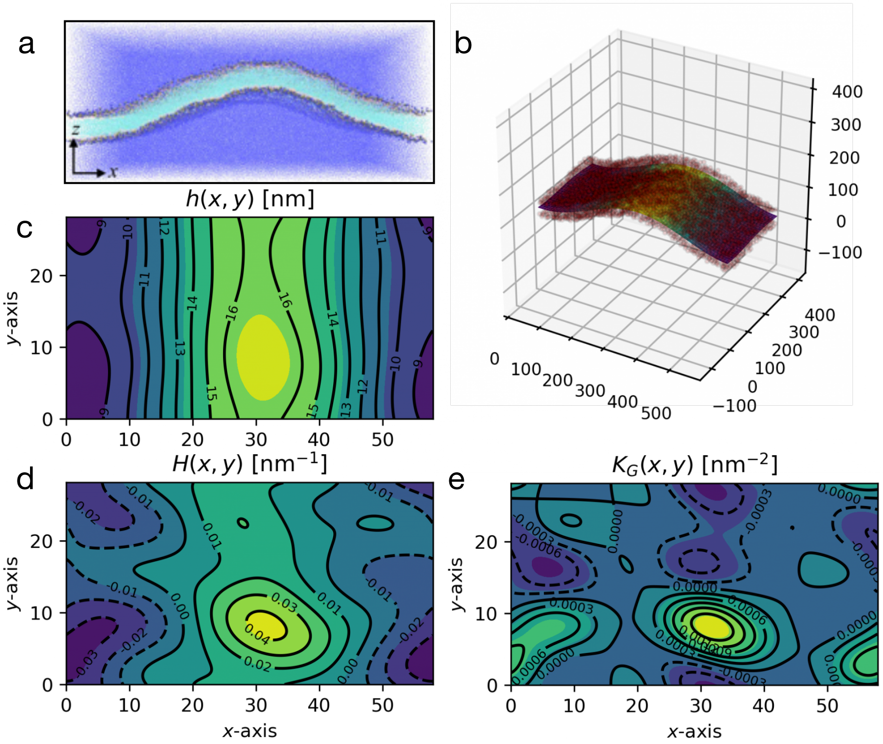

Supplementary Figure 15. **Membrane curvature in simulations of buckled empty bilayer.** (**a**) A typical snapshot from buckled empty bilayer simulations showing the lipid head groups (orange spheres), acyl chains (cyan sticks) and water (blue diffuse spheres). (**b**) The buckled membrane profile (grey surface) was approximated by height function $h(x, y)$ described by a 2D Fourier expansion. The Fourier coefficients were obtained by least-square fitting of the truncated Fourier expansion to the lipid phosphate bead positions (red transparent spheres). (**c**) Contour plot of the optimized membrane surface, evaluated on a grid spanning the periodic box (57×28 nm^2), as the local height function, $h(x, y)$. (**d-e**) Contours of mean curvature $H(x, y)$ and Gaussian curvature $K_G(x, y)$ computed as the determinant and half the trace of the local shape operator, respectively. Contour lines corresponding to positive and negative values are indicated by solid lines and dashed lines, respectively.

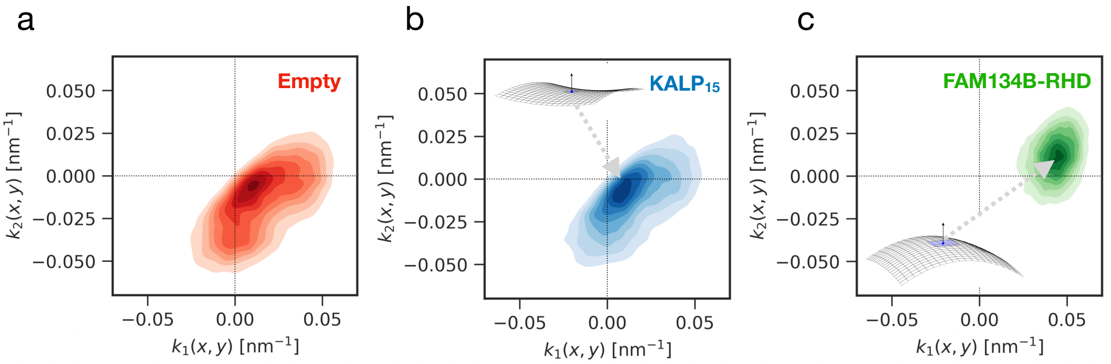

Supplementary Figure 16. **Curvature sampling on buckled membrane.** 2D-histograms (density indicated by color intensity) of the principal curvatures k_1 and k_2 for (**a**) the empty sinusoidal membrane buckle (red), and the buckle with the (**b**) KALP_{15} peptide (blue) and (**c**) FAM134B-RHD (green). The eigenvalues of the local shape operator $\mathbf{S}(x,y)$ were sorted as $k_1(x,y) \geq k_2(x,y)$. The insets shows local membrane shapes (wire frame of a 3×3 nm^2 patch) of the sampled points along the buckle (local tangent planes in blue). The RHD samples regions of high local mean curvature, $\langle H(x,y) \rangle = 0.026$ nm^{-1}, that resemble spherical caps ($k_1(x,y) > 0$ and $k_2(x,y) > 0$). The KALP_{15} peptides samples saddle-like regions of the buckle with both positive and negative curvatures ($k_1(x,y) \geq 0$ and $k_2(x,y) \leq 0$). For the empty membrane, points in the xy plane were sampled randomly. Due to the lateral compression of the buckled membrane, the empty bilayer favors gentle saddle shapes ($k_1(x,y) \geq 0$ and $k_2(x,y) \leq 0$) that minimize the total mean curvature ($H(x,y) \approx 0$).

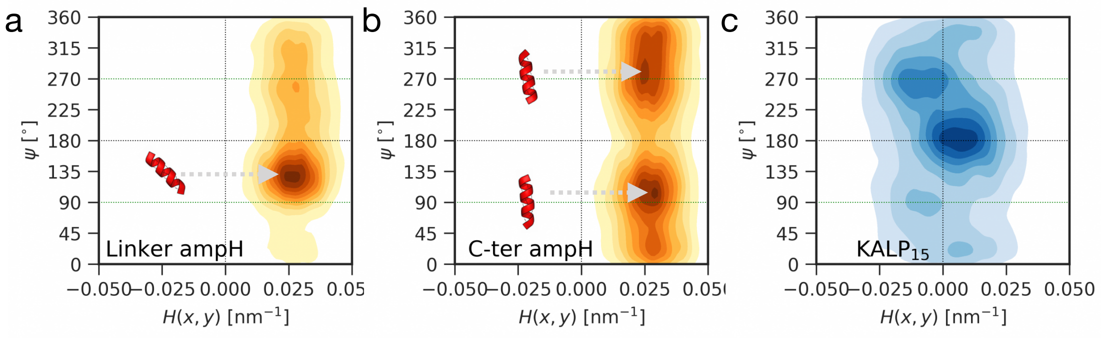

Supplementary Figure 17. **FAM134B-RHD orientation in buckled membranes.** (**a**) AH_L and (**b**) AH_C of the FAM134B-RHD display specific orientations with respect to the local membrane curvature field defined by $\mathbf{S}(x, y)$. The orientation of the amphipathic helix orientation was quantified in terms of the angle ψ between the projection of the helix long-axis onto the xy plane and the unit eigenvector $\hat{\mathbf{k}}_1$ of $\mathbf{S}$ corresponding to eigenvalue k_1, evaluated at the protein center-of-mass position. 2D-histograms (density contours orange) of the angle ψ show that AH_L orients diagonally ($\psi \approx 135°$) with respect to the principal curvature $\hat{\mathbf{k}}_1$. AH_C prefers a more perpendicular orientation ($\psi \approx 90°$; $\psi \approx 270°$). (**c**) For the $KALP_{15}$ peptide, the projection of the tilted TM helix long-axis onto the xy plane was used to compute its orientation with respect to the local principal curvature axis $\hat{\mathbf{k}}_1$. The dominant peak in the distribution indicates a preferred alignment of the TM helix-tilt with the local principal curvature ($\psi \approx 180°$).

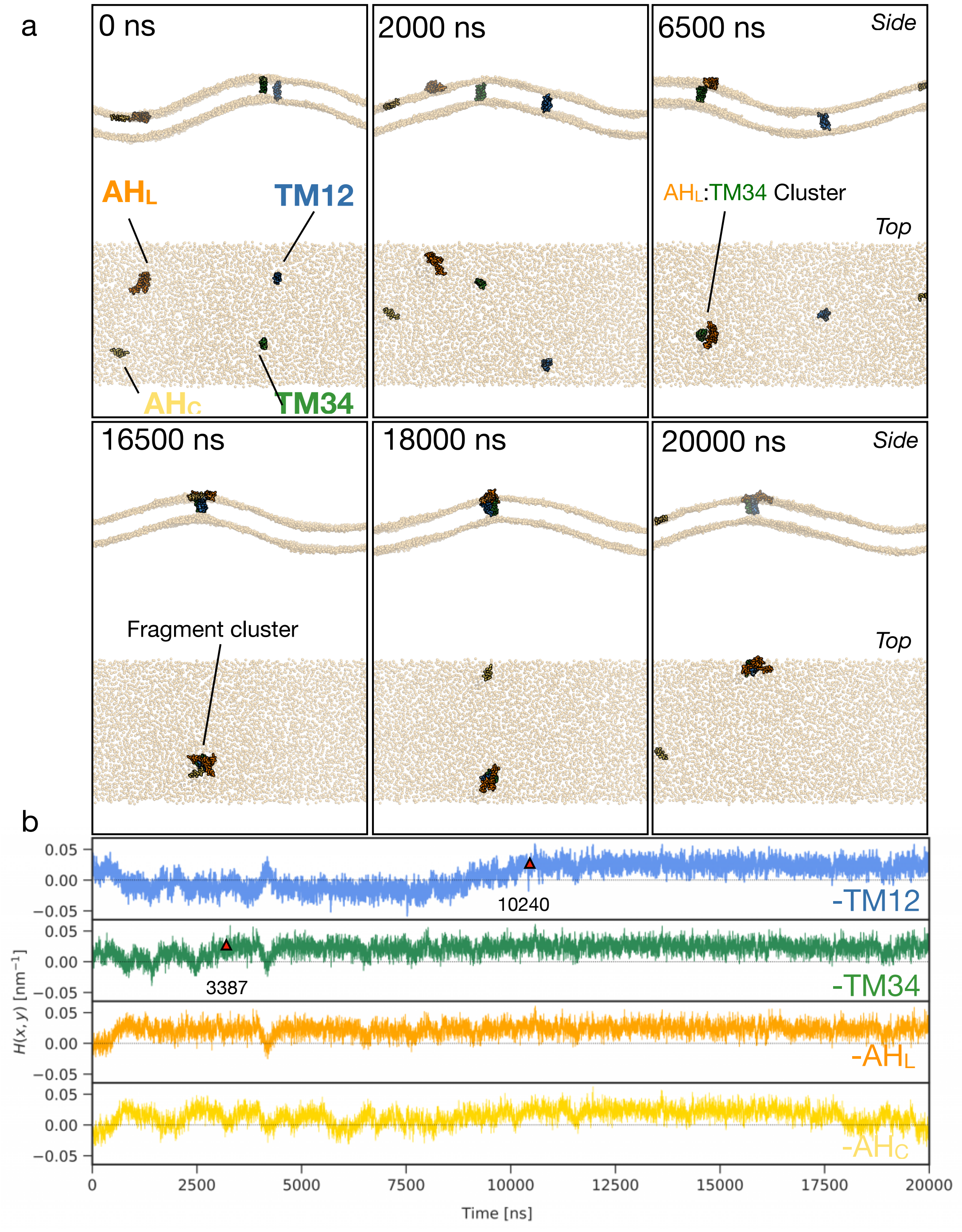

Supplementary Figure 18. **Curvature sampling by FAM134B-RHD fragments.** (**a**) Coarse-grained simulations of individual FAM134B-RHD fragments embedded in buckled membrane. TM hairpins (TM12 and TM34) and AH containing fragments (AH_L and AH_C) were initially placed in high and low curvature regions of the buckle. Fragments preferentially diffuse to highly curved region and cluster with the linker/AH_L fragment (orange). (**b**) Time series of local curvatures sampled by individual fragments. Red triangles indicate times when individual hairpin fragments (blue/green) clustered with linker/AH_L fragment on top of the buckle. The short terminal AH_c fragment (yellow) displays several association and disassociation events with the rest of the AH_c:TM34:TM12-cluster.

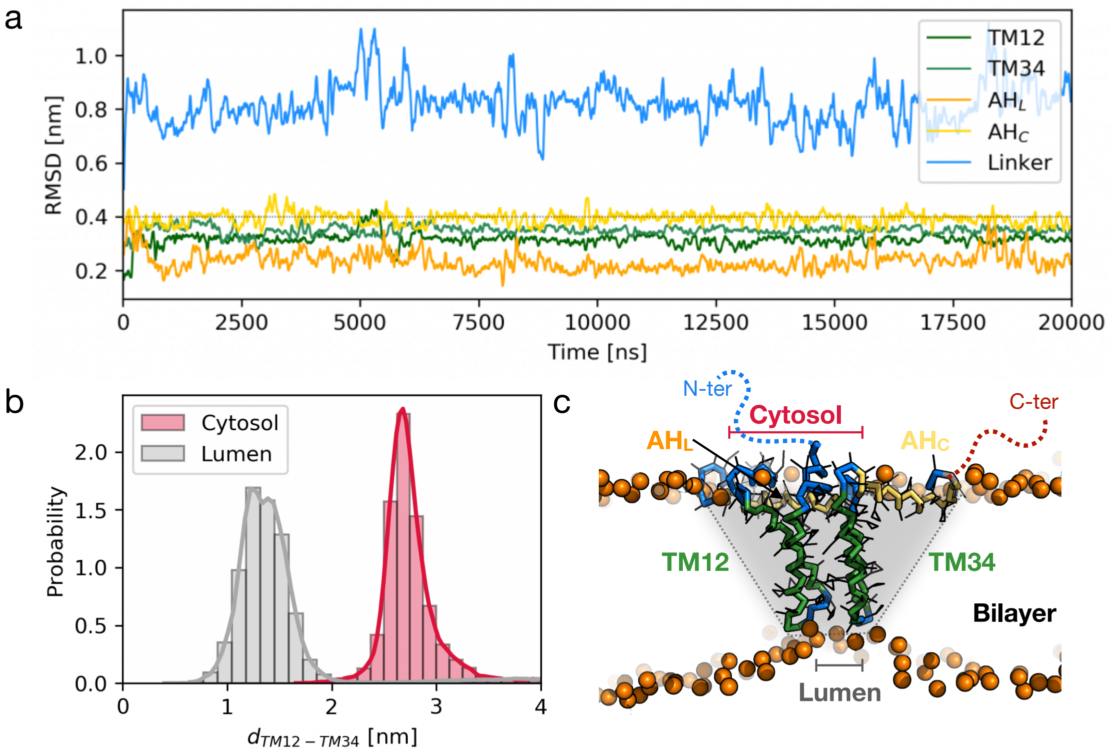

Supplementary Figure 19. **FAM134B-RHD forms a wedge-shaped membrane inclusion.** (**a**) RMSD of backbone beads of individual fragments computed using refined fragment structures as reference in long coarse-grained simulations of the FAM134B-RHD. Conformational changes are prominent in the water-exposed linker fragment (blue) despite the presence of a rigid AH_L (RMSD < 4.0 ; orange). The rest of the fragments, i.e. TM regions (green) and the AH_C show no drastic structural changes (RMSD < 4.0). (**b**) Inter-hairpin distance varies across the cytosolic leaflet (crimson; $d = 2.83 \pm 0.45$ nm) and luminal leaflets (grey; $d = 1.51 \pm 0.67$ nm). (**c**) Structure of FAM134B-RHD from coarse-grained simulations in flat bilayers with PBC. The unique topology of the RHD promotes an overall wedge-shaped inclusion (grey outlined) driving membrane curvature. Bilayers coupled by short TM hairpin structures are asymmetrically stretched by the action of amphipathic helices at the cytosolic leaflet leading to enhanced curvature induction and sensing.

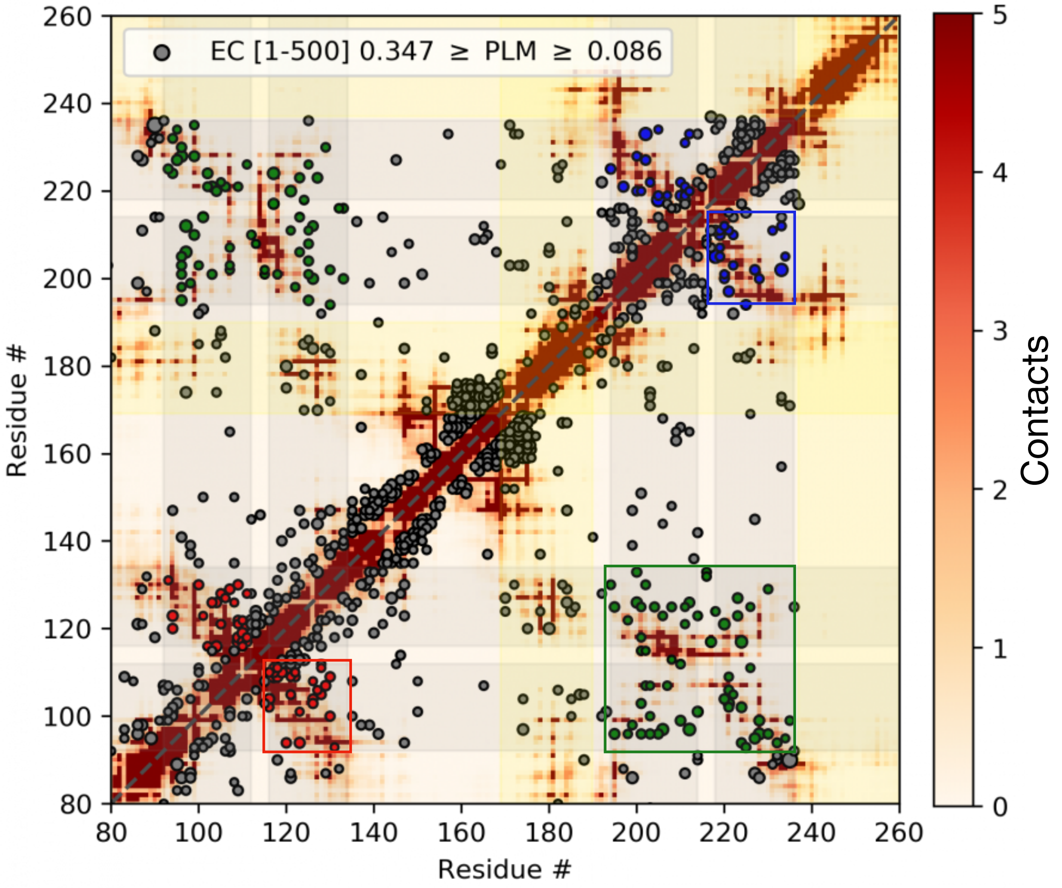

Supplementary Figure 20. **Overlap of residue contacts and evolutionary residue covariance within FAM134B-RHD.** Predicted coevolving residue pairs (filled circles) are spatially proximal and display residue contacts within the RHD structure (heat map, orange-to-red). Non-trivial contacts within the TM regions (grey background) and AH regions (yellow background) stabilize the RHD structure. The structural residue-contact map was obtained by averaging pair-wise residue interactions (CG-bead contacts within 1 nm) from MD simulation of FAM134B-RHD (20 μs). Dark maroon indicates a high number of inter-residue contacts on average (scale on right). The contact map is superimposed with the top 500 coevolving residue pairs (circles scaled by the strength of the contact in the range $0.347 \geq$ PLM ≥ 0.086) computed from RHD-containing homologs ($n = 1380$) using EVFold[12,13]. Structural residue interactions between TM regions (colored squares) show good overlap with predicted coevolving residue pairs, i.e., TM1-TM2 (red circles), TM3-TM4 (blue circles), and TM12-TM34 (green circles).

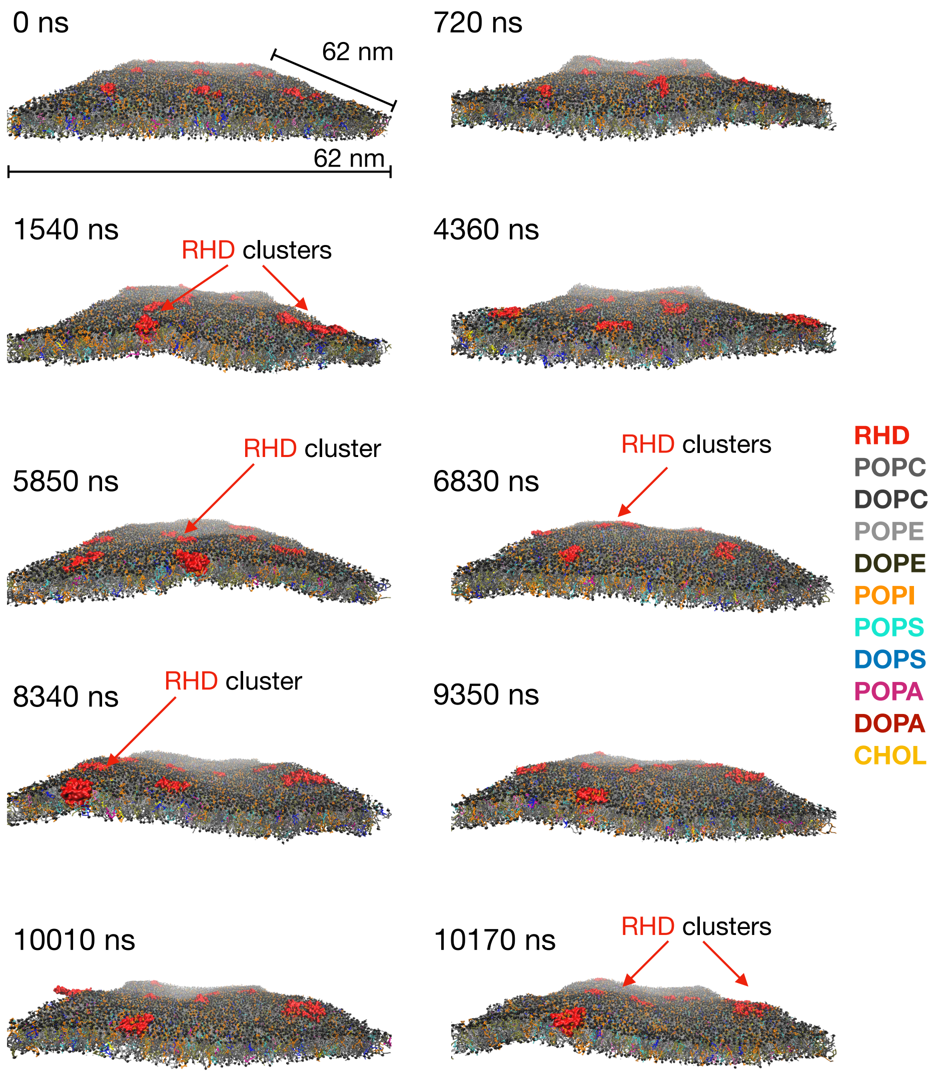

Supplementary Figure 21. **RHD clustering in flat membranes under periodic boundary conditions.** Selected snapshots of coarse-grained simulation of large membrane patch (62 x 62 nm; black PO4 beads) with ER-lipid composition (colored legends). Nine FAM134B-RHD molecules (red), initially placed 2 nm apart on a rectangular grid diffused laterally along the membrane inducing local curvature. Although PBC of the simulation box attenuate curvature effects, the transient clusters (labeled; red arrows) formed by the the RHDs result in locally curved membrane shapes.

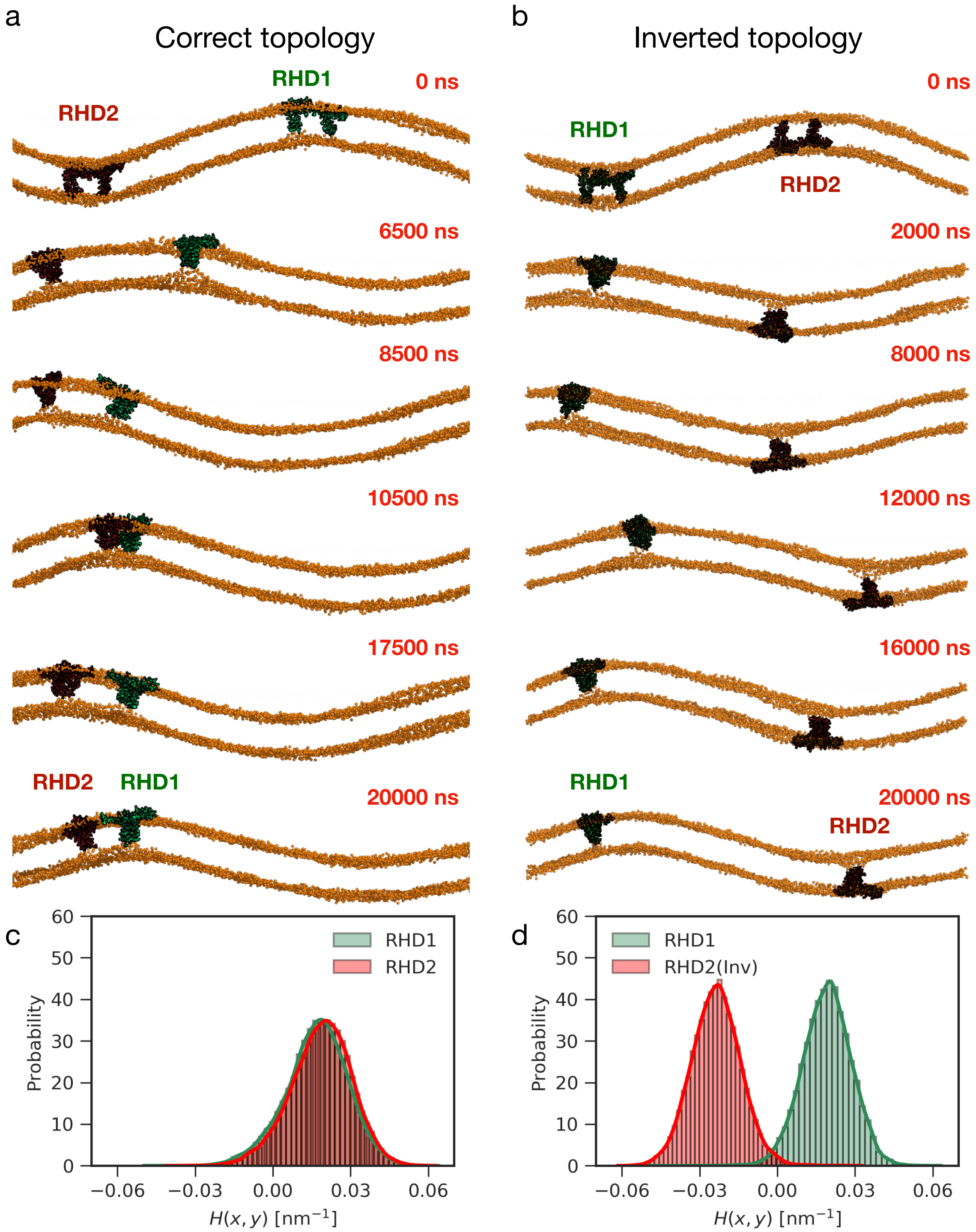

Supplementary Figure 22. **Protein sorting and RHD-clustering in curved membranes.** Selected snapshots from simulations of two FAM134B-RHD molecules (red and green) embedded in buckled membrane (orange PO4 beads). Second FAM134B molecule (RHD2: red) is embedded in (**a**) correct and (**b**) inverted orientation to assess the effect of protein topology on curvature mediated clustering. (c and d) Probability distributions of local mean curvatures $H(x, y)$ sampled by the two RHD molecules are sampled every 1 ns from the 20 μs trajectories.

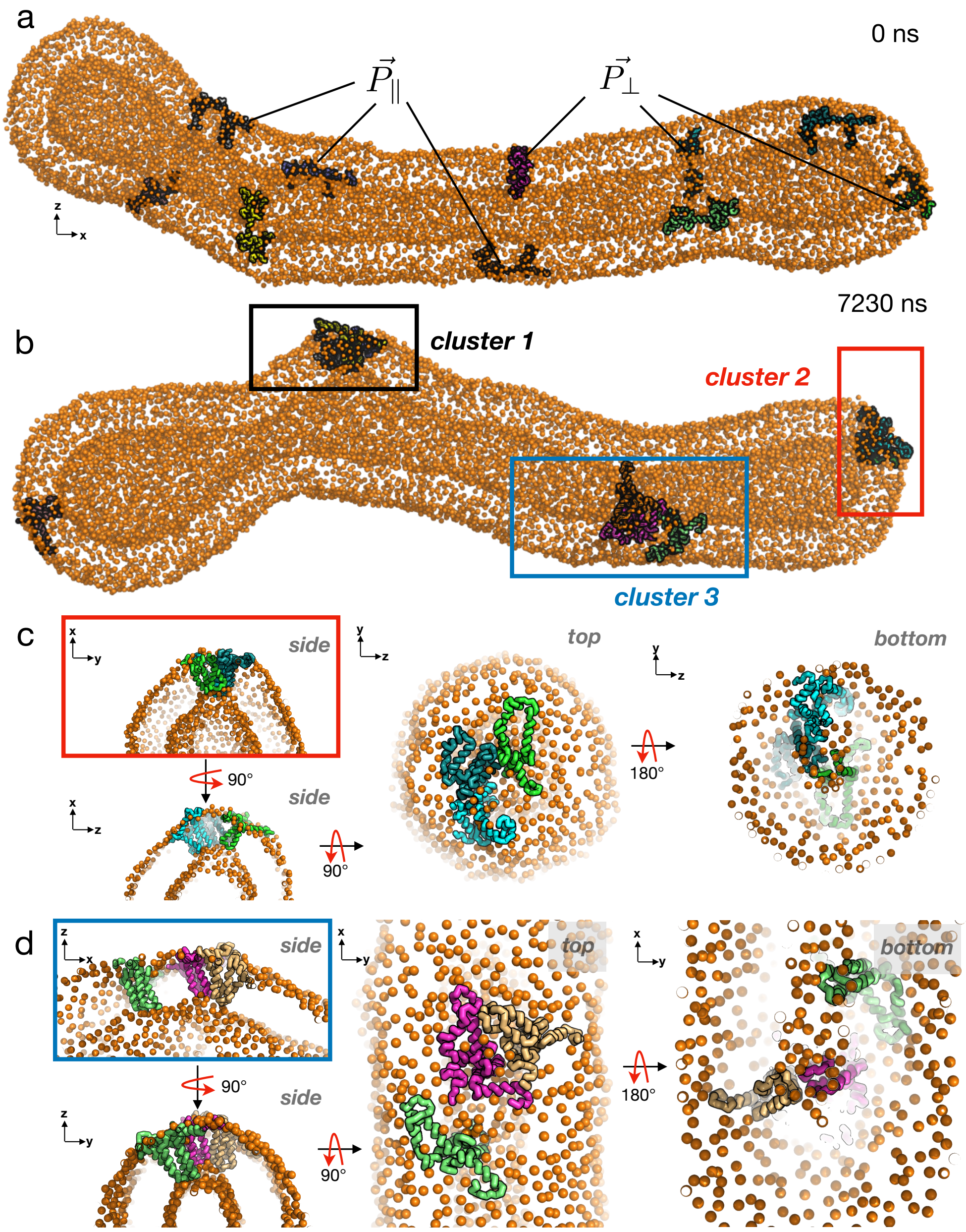

Supplementary Figure 23. **RHD-clusters form inverted pyramid-shaped inclusions and deform tubule structure.** (**a**) Initial configuration of a closed tubular membrane (orange) with 10 FAM134B-RHD molecules (colored chains) used for MD simulation with explicit solvent (3.6×10^6 water beads; not shown). RHDs were initially embedded such that there were equidistantly spaced from each other and in parallel ($\vec{P}_{\parallel}$) and perpendicular ($\vec{P}_{\perp}$) orientations to the tubule long-axis (labelled). Curvature mediated sorting results in protein clusters at highly curved regions of the tube. (**b**) Deformed tubule structure showing three protein-clusters (clusters 1-3), each containing 3 RHDs. (**c-d**) Zoom-up of the cluster-2 and 3 with cross sections showing side, top and bottom views of the clusters (see Fig. 5 for cluster 1). Cluster 1 and 2 resemble inverted pyramidal wedges, cluster 3 formation is incomplete with 2 strongly interacting RHDs and a weekly associated RHD (green chain in **d**). Close contact among the luminal hydrophilic loops of the RHD-cluster form a conical protein projection into the lumen, while the rigid AHs act as spacers on the cytosolic face forming an inverted pyramid shaped wedge in the membrane.

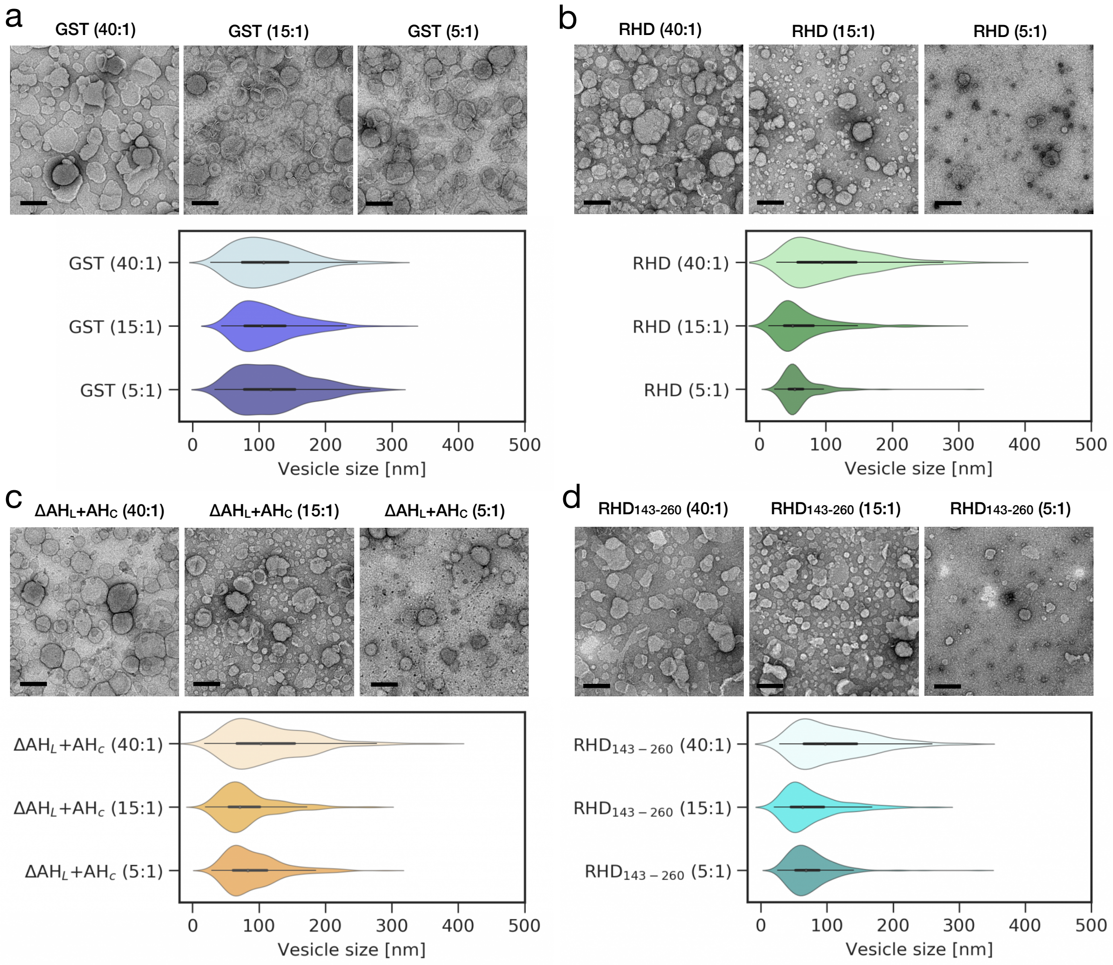

Supplementary Figure 24. **FAM134B-RHD remodels liposomes in a concentration-dependent manner.** Representative negative-stain transmission electron micrographs (nsTEM) of remodeled liposomes obtained after incubation of empty liposomes with purified (**a**) GST, (**b**) wild-type FAM134B-RHD, (**c**) $\Delta AH_L + AH_C$, and (**d**) $RHD_{143\text{-}260}$ for 18 h at 22 °C. Increasing proteins concentrations were incubated with liposome samples to obtain a different lipid-to-protein rations (left to right, 40:1, 15:1, and 5:1) respectively. Each sample was also subjected to image analysis using ImageJ[14] to characterize the overal distribution of sizes of remodeled liposomes (Scale bars 200 nm; violin plots, $n = 300$ each case). Violin plots show central box-plots (median with interquartile range, black line) with mirrored histograms on either sides (shaded by protein concentration).

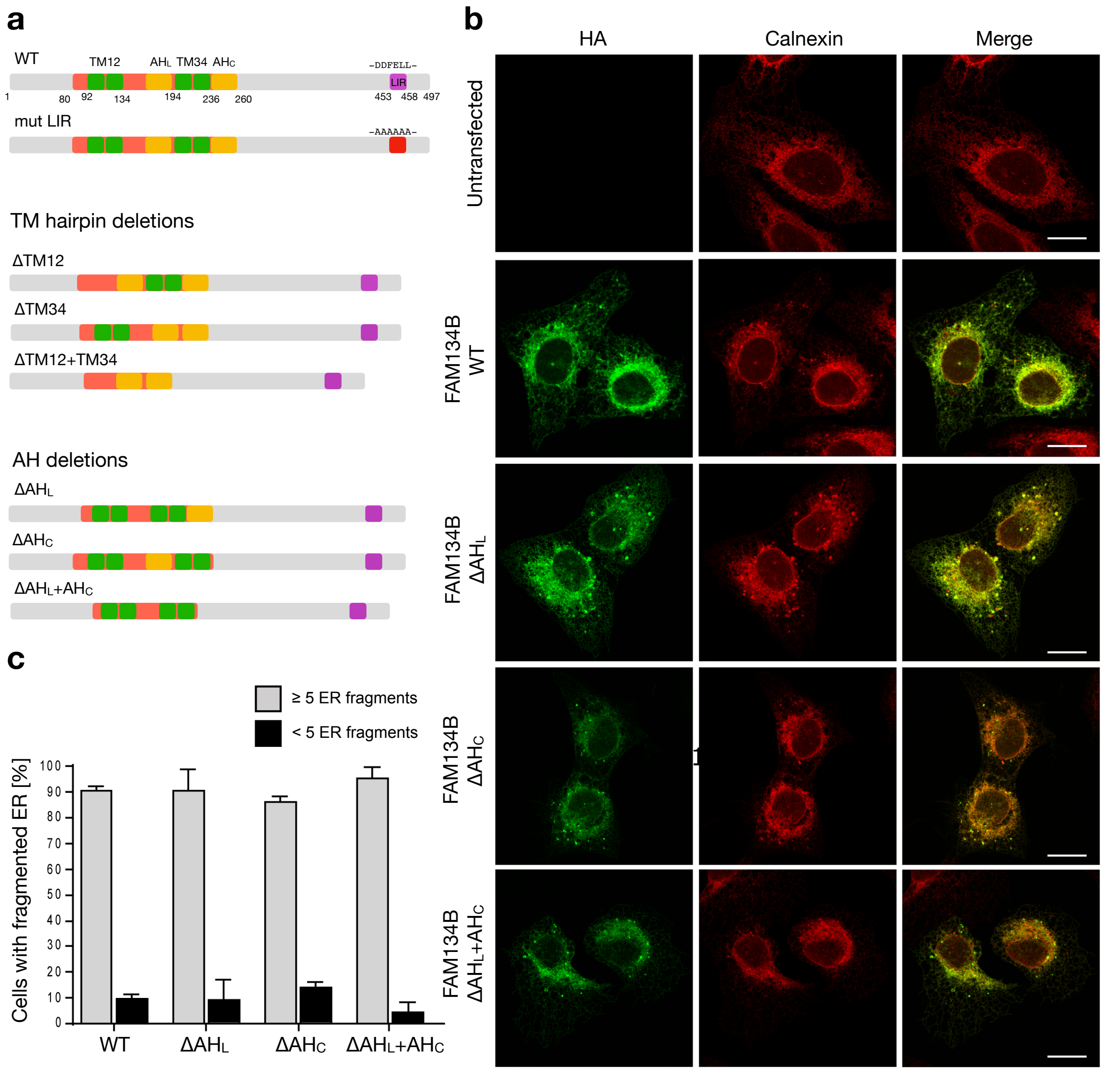

Supplementary Figure 25. **Role of AH segments in ER-fragmentation.** (**a**) Schematics of FAM134B WT, LIR mut, TM, and AH deletion constructs used for transient transfections and IF studies. (**b**) Immunofluorescence of HA and endogenous calnexin in U2OS cells transiently overexpressing HA tagged FAM134B (top to bottom): Untransfected, wildtype (WT), single AH deletions (ΔAH_L and ΔAH_C), and the double AH deletion (ΔAH_L+AH_C). Scale bars 10 μm. (**c**) Quantification of U2OS cells with fragmented ER ($\geq$ 5 ER fragments per cell) after transient over-expression of wild-type and mutant forms of FAM134B.

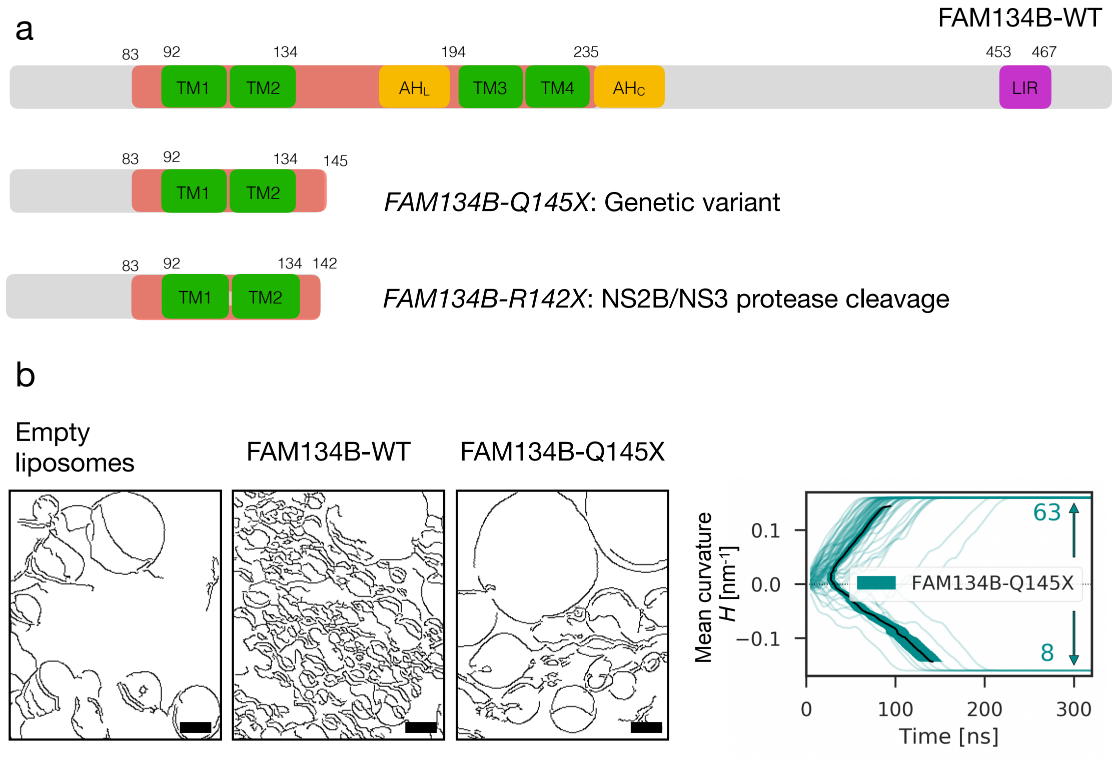

Supplementary Figure 26. **Disruption of FAM134B-RHD slows down membrane curvature induction.** (**a**) Schematic of WT and truncated variants of FAM134B. Truncation variants split the RHD and are a result of genetic polymorphism (Q145X), or NS2B/NS3 mediated proteolytic cleavage product (R142X) during Ebola and Zika vital infections. (**b**) Schematics of protein-liposome complexes redrawn from original freeze fracture EM studies on protein-liposome complexes after flotation[15] (Scale bar 200 nm). Insertion of FAM134B-WT protein in liposomes drastically reduced the liposome size in experiments. For the truncation mutant and control (empty liposomes), larger vesicles were observed. In the patch-closure simulations, the FAM134B-Q145X truncation variant (cyan) induces patch-closure more slowly and with less directionality.

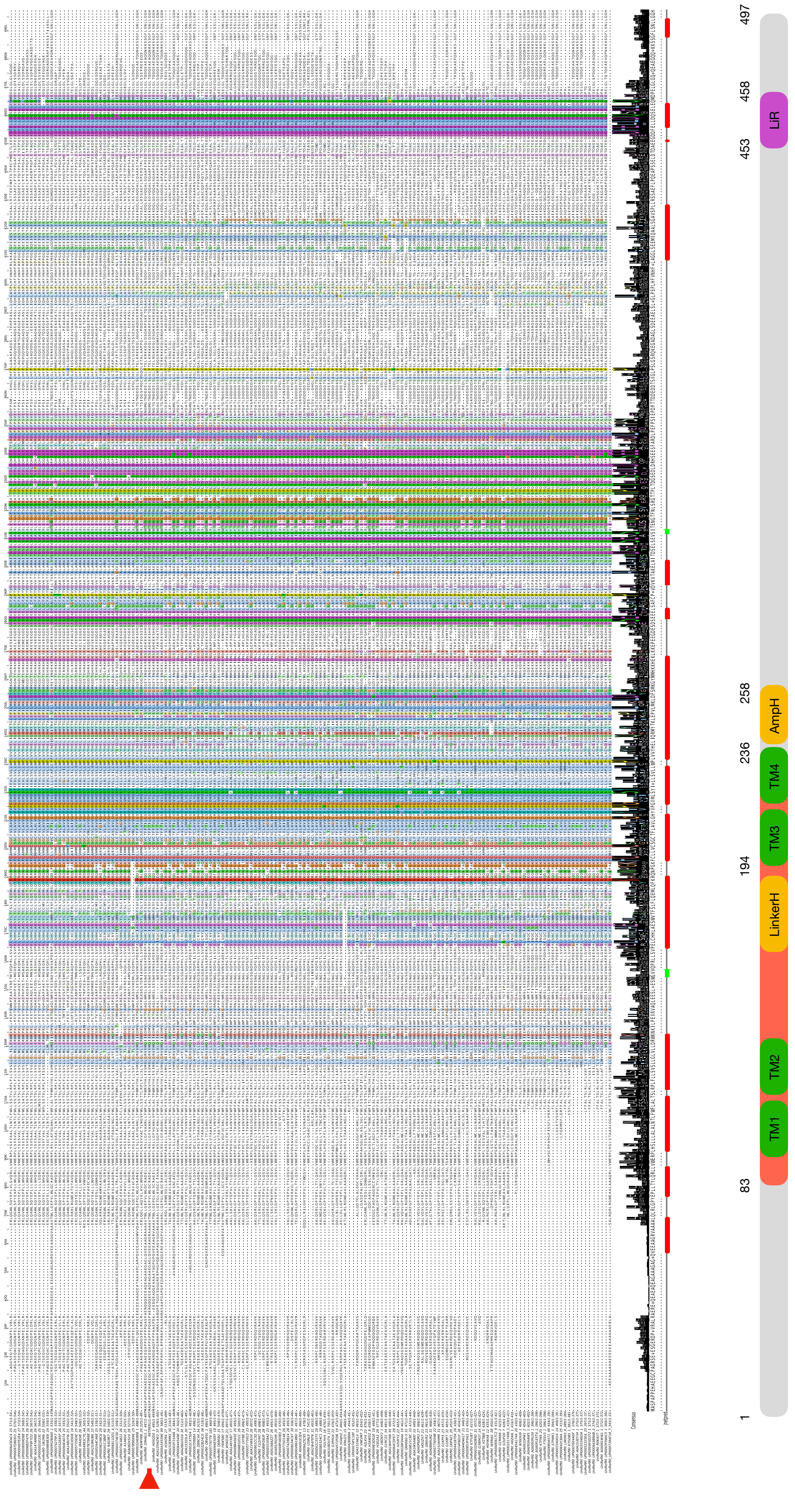

Supplementary Figure 27. **FAM134B conservation.** Schematic showing the MAFFT multiple sequence alignment of FAM134B (red triangle) and its close homologs obtained after 5 iterations of PSI-BLAST against NCBI database showing conserved regions (columns with > 30 % conservation are colored according to ClustalX scheme). Summary of various regions of sequence is provided at the bottom, along with secondary structure prediction and family consensus. See supporting alignment file for details.

SUPPLEMENTARY TABLES

Supplementary Table S1. **Comparative modeling of FAM134B-RHD fragments.** Sequences corresponding to individual fragments of the FAM134B-RHD were used to identify template protein structures from the PDB using Raptor-X[16]. RaptorX is a template-based structure prediction tool known to detect remote target-template relationships with preserved secondary structure patterns (position and lengths). High scoring alignments from remote-templates were used to obtain the rough initial 3D fragment structures. These models resemble template fragment 3D structures (RMSD) despite low sequence identity.

Target fragment	Sequence position	Template protein (Organism)	PDB code	Seq. id./ sim. (%)	RMSD (Å)
N-term	80-91	N-Myristroyl transferase (*E. coli*)	2wskA	8.33/ (8.33)	2.3
TM12	92-134	Respiratory complex I (*E. coli*)	3rkoC	13.95/ (23.25)	0.94
Linker/ AH_L	135-193	STAT-1 (*E. coli*)	1bf5A	18.42/ (27.90)	4.8
TM34	194-236	Glutaryl-CoA dehydrogenase (*T. thermophilus*)<br>Butaryl-CoA dehydrogenase (*M. elsdenii*)	2ebaA<br>1bucA	7.69/ (18.60)	2.67
C-term/ AH_C	237-260	Angiotensin-converting enzyme 2 (*H. sapiens*)	1r42A	8.57/ (28.57)	1.75

Supplementary Table S2. **Refinement of FAM134B-RHD fragments.** Extensive coarse-grained (CG) and all-atom (AA) MD simulations of membrane-embedded and membrane-adhered fragments were used to obtain final refined fragment structures. Coarse-grained simulations of individual fragments were used to sample conformations and obtain stable conformational states. These simulations also provided optimal position and orientation information with respect to the bilayer, and allowed us to assess peripheral membrane binding of amphipathic helices and membrane anchoring and thinning by helical hairpins.

Fragment	Model AA/CG	N_{Lipids}	N_W	N_{Ions} Na^+/Cl^-	N_{Total}	Simulation time (μs)
TM12	CG	434	10157	109/114	16146	102.637
TM12-c1	AA	135	4453	10/15	32376	2.463
TM12-c2	AA	135	4415	10/15	32262	1.080
TM34	CG	437	10150	111/112	16167	105.078
TM34-c 1	AA	136	4852	12/13	33603	6.308
TM34-c2	AA	135	5265	12/13	34708	6.036
TM34-c3	AA	136	5343	12/13	35076	4.729
Linker	CG	450	10082	110/112	16320	23.058
Linker	AA	Sol	23658	67/69	72290	3.522
Linker	AA	283	13476	42/44	79616	3.199
C-ter	CG	450	10147	111/113	16270	12.561
C-ter	AA	Sol	5960	17/19	18281	3.959
C-ter	AA	297	9909	31/32	68614	5.438
C-ter	AA	287	11342	36/38	72923	3.459

Supplementary Table S3. **Molecular dynamics simulations of intact FAM134B-RHD.** Coarse-grained (CG) and all-atom (AA) MD simulations were performed on FAM134B-RHD to relax and refine the intact FAM134B-RHD structure, respectively. All-atom MD simulations were also used to analyze the local membrane structure after equilibration. The coarse-grained symmetric ER membrane model was built using *insane.py* to match relative lipid head group composition observed in the mammalian ER membranes. Equal proportions of C34:1 and C36:2 chains were used to model acyl chains with individual head groups (PC, PE, PI, PS, and PA) to mimic the saturation content of the ER membrane.

System	Lipid (N_{Lipids})	N_W	N_{Ions} (Na^+/Cl^-)	N_{Total}	Simulation time (μs)
RHD_{AA}	POPC (626)	29433	80 /88	175346	2.42
RHD_{CG}	POPC (1308)	53576	586/584	72181	28.66
RHD_{CG}	POPC (326)<br>DOPC (326)<br>POPE (195)<br>DOPE (195)<br>POPI (130)<br>POPS (51)<br>DOPS (38)<br>POPA (12)<br>DOPA (12)<br>CHOL (12)	50040	615/486	67314	37.19

Supplementary Table S4. **In silico vesiculation simulations.** Coarse-grained MD simulations were performed to characterize bilayer-to-vesicle and bicelle-to-vesicle transitions. POPC bilayers and bicelles (DMPC+DHPC) with $KALP_{15}$, FAM134B-RHD, FAM134B variants and individual fragments were used to probe their curvature-induction abilities. From 100 replicates simulated for each system, data were collected from n_{runs}. Remaining runs could not be analyzed as the bilayer/bicelles discs fused with their periodic images to form a continuous bilayer system.

System	N_{Lipids}	N_W	N_{Ions} Na^+/Cl^-	N_{Total}	Simulation time (ns)	n_{runs}
Empty bilayer disc	1352	112222	587/587	130972	100 × 500	80
$KALP_{15}$	1344	112192	585/589	130866	100 × 500	78
FAM134B-RHD	1308	112311	582/590	130908	100 × 500	93
FAM134B-Q145X	1339	112274	587/593	131012	100 × 500	71
FAM134B-RHD	1297_{ER}	109582	615/486	126856	100 × 500	95
TM12	1339	112229	585/588	130903	100 × 500	72
TM34	1342	112191	586/587	130902	100 × 500	74
C-ter/AH_C	1349	112176	589/591	130942	100 × 500	85
Linker/AH_L	1330	112217	589/591	130853	100 × 500	66
Empty bicelle	1352+500	213156	0/0	230676	100 × 1000	97
$KALP_{15}$	1340+498	213151	0/4	230567	100 × 1000	96
FAM134B-RHD	1298+496	212823	0/9	230195	100 × 1000	95
TM12	1328+499	213151	0/3	230520	100 × 1000	97
TM34	1332+499	213153	0/1	230558	100 × 1000	96
C-ter/AH_C	1323+497	213113	0/2	230487	100 × 1000	100
Linker/AH_L	1340+500	213129	0/2	230580	100 × 1000	99

Supplementary Table S5. **Kinetics of in silico curvature induction.** Coarse-grained MD simulations were performed to study bilayer-to-vesicle and bicelle-to-vesicle transitions. The vesicle formation was characterized by an increase in curvature, $\pm k$. The numbers n_+ and n_- indicate vesicle transitions with bilayer curvature away and towards the upper, cytoplasmic leaflet. The P-values indicate the one-tailed probability in binomial tests, $P(n \geq n_+)$ in a total of $n_+ + n_-$ trails with $p = 0.5$. Waiting times (t) for vesicle formation were recorded as the time taken for the bilayer or bicelle discs to reach a curvature of $|H| = 0.15$ nm^{-1}. The vesicle formation rate $k_{\text{sys}} = 1/(t' + \tau)$ was estimated from exponential fits of the cumulative distribution functions of Poisson distributed waiting times, t' and a constant lag time, τ. The acceleration in vesicle formation due to a protein inclusion were estimated as the ratio $k_{\text{sys}}/k_{\text{POPC}}$. Due to the small number of spontaneous bicelle-to-vesicle transitions observed for the pure bicelle, bicelles with KALP_{15}, TM12, and TM34 fragments we could not estimate rates from fitting observed waiting times. Instead, we report a maximum likelihood estimate of the rates for vesicle formation.

System	n_{runs}	$\frac{n_+}{n_+ + n_{n_-}}$	p-value	$t_{\text{mean}} \pm$ s.d. (ns)	t' (ns)	τ (ns)	$\frac{1}{(t'+\tau)}$ (ns^{-1})	$\frac{k_{\text{sys}}}{k_{\text{POPC}}}$
Empty bilayer	80	38/80	0.368	130.01 ± 45.22	67.07	71.74	0.0072	1.00
KALP_{15}	78	40/78	0.454	98.96 ± 29.71	39.37	62.78	0.0097	1.35
FAM134B-RHD	93	93/93	$< 10^{-6}$	59.15 ± 7.79	13.61	47.94	0.0162	2.25
FAM134B-Q145X	71	63/71	$< 10^{-6}$	95.30 ± 33.24	38.59	58.76	0.0102	1.42
TM12	72	51/72	0.0003	96.26 ± 28.02	42.75	59.00	0.0098	1.36
TM34	74	38/74	0.4537	102.59 ± 26.53	44.11	65.48	0.0091	1.26
C-ter /AH_C	85	82/85	$< 10^{-6}$	108.91 ± 39.51	50.70	62.49	0.0088	1.22
Linker/AH_L	66	66/66	$< 10^{-6}$	77.45 ± 13.74	23.22	57.87	0.0123	1.71
Empty bicelle	97	2/2	0.2397	-	-	-	1.09×10^{-5}	1.00
KALP_{15}	96	4/5	0.1855	-	-	-	5.19×10^{-5}	4.72
FAM134B-RHD	95	92/92	$< 10^{-6}$	534.21 ± 176.08	235.7	320.4	0.0017	163.48
TM12	97	18/24	0.0123	> 755.58 ± 96.84	-	-	1.35×10^{-5}	1.23
TM34	96	22/37	0.1619	> 819.46 ± 162.22	-	-	1.67×10^{-5}	1.53
C-ter /AH_C	99	42/43	$< 10^{-6}$	776.54 ± 182.34	339.34	465.14	0.0012	110.09
Linker/AH_L	100	91/92	$< 10^{-6}$	725.52 ± 234.63	417.74	347.99	0.0013	119.26

Supplementary Table S6. **Molecular dynamics simulations of curvature sampling and clustering by FAM134B-RHDs.** Coarse-grained (CG) buckled membranes were simulated for estimating curvature preferences of FAM134B and its constituent fragments. To test the effect of membrane curvature on protein-clustering, a large ER-model membrane with FAM134B-RHD molecules was simulated by replicating the previous system 9 times along the x-y plane (see Supplementary Table 2). To demonstrate curvature-mediated protein sorting/clustering, proteins with correct (2 RHDs) and inverted topologies (2 RHDs′) were embedded in buckled membrane and simulated. Simulations of a large closed tubule with 10 RHD molecules was used to study cluster formation and organization in curved bilayer.

System	Lipid (N_{Lipids})	N_W	N_{Ions} (Na^+/Cl^-)	N_{Total}	Simulation time (μs)
Buckle	POPC (5322)	283682	- /-	352868	23.04
Buckle + $KALP_{15}$	POPC (5312)	283661	0/4	352749	53.32
Buckle + RHD	POPC (5264)	283626	0/8	352487	50.56
Buckle + Fragments	POPC (5233)	283603	0/8	352041	25.30
Flat bilayer + 9 RHDs	POPC (2934)<br>DOPC (2934)<br>POPE (1755)<br>DOPE (1755)<br>POPI (1170)<br>POPS (459)<br>DOPS (342)<br>POPA (108)<br>DOPA (108)<br>CHOL (108)	450360	5535/4374	67314	12.74
Buckle + 2 RHDs	POPC (5186)	283502	0/16	351778	22.91
Buckle + 2 RHDs′	POPC (5177)	283549	0/16	351708	22.88
Tubule + 10 RHDs	POPC (9616)	3699934	0/80	3829232	10.00

SUPPLEMENTARY METHODS

Fragment conformational sampling and refinement

Initial TM fragment structures (TM12 and TM34; Supplementary Table 2) were first transformed into a CG representation, embedded in lipid bilayers, and subjected to long equilibrium simulations ($\approx 100\ \mu$s) for conformational sampling. Long-lived (>20 μs), highly populated fragment conformations were obtained after clustering evenly sampled conformations (n = 10000). Clusters were obtained using backbone RMSD (cut-off = 3.0) by employing the *gromos* method, as implemented in the *g_ cluster* tool[17]. Representative structures corresponding to top clusters for each fragment, were back-mapped to all-atom representation using *backward.py*[18] followed by all-atom simulations for fragment structure refinement. Cross-helical interactions, characteristic of TM hairpins, were quantified by measuring helix-helix contacts,

$$HH_{\text{contacts}} = \left[\sum_{i \in h_1} \sum_{j \in h_2} \sigma(|r_{ij}|) \right] \quad (1)$$

where the sums extend over heavy atoms (beads in the CG models) of interacting helices and $\sigma(|r_{ij}|)$ is a smooth sigmoidal counting function to limit interactions below cut-off ($r_{ij} \leq 6$ Å and ≤ 10 Å for all-atom and CG models, respectively).

CG simulations of Linker and C-terminal fragments were performed in the presence of lipid bilayers to study membrane docking and binding. These simulations confirmed the assignments of the membrane-binding amphipathic helices, and provided detailed information on their membrane-binding modes. Correct fragment binding-modes were back-mapped and used to initiate all-atom MD simulations for further model refinement. Refined fragment models were obtained after $\approx 3\ \mu$s simulation in lipid bilayers. Additional solution simulations were performed for fragments with presumed amphipathic helices (i.e., Linker and C-ter fragments) to study helix stability in solution. All simulations performed for sampling and assessment of fragment models are summarized in Supplementary Table 2.

In silico curvature induction: Patch closure simulations

Discontinuous POPC (16:0-18:1 PC) bilayer patches (20×20 nm^2) with a bilayer-embedded peptide or protein were centered in a large cubic box ($25 \times 25 \times 25$ nm^3) and solvated with CG water and 150 mM NaCl. The initial system configuration was energy minimized, and equilibrated with position restraints on the protein backbone particles. Additional position restraints on phosphate beads along the z-direction were applied during system equilibration. This procedure restricted the bilayer curvature while ensuring free mixing of lipids in the xy plane and the formation of bilayer discs during the equilibration phase. A similar procedure was employed to construct a membrane patch with ER-lipid composition. Discontinuous bicelle systems containing saturated DMPC (14:0 PC) and DHPC (7:0 PC) lipids were assembled using a similar protocol. First, a discontinuous bilayer patch of DMPC embedded with protein was built and equilibrated to obtain a flat circular disc. Then, excess DMPC molecules were added using the gromacs:*genbox* tool close to the hydrophobic open edges of the DMPC disc and allowed to equilibrate. DHPC molecules preferentially coated the hydrophobic edges of the disc and integrated with the bilayer edge, forming a bicelle. Non-interacting excess DHPC molecules in solution were removed to obtain well equilibrated flat bicelle structures with DMPC and DHPC (338:125).

In the production phase, position restraints on phosphate beads were removed to study bilayer disc and bicelle shape changes. Simulations were performed for 1 μs using a 20 fs time step.

Bilayer discs and bicelles with $KALP_{15}$ peptide and without protein served as controls. We employed an anisotropic Parrinello-Rahman barostat[19] to avert possible fusion of the bilayer discs and bicelles across the periodic boundary (in the xy plane). Additionally, bilayer discs with individual FAM134B-fragments and the truncation variant, FAM134B-RHD-Q145X were simulated. 100 replicates for each system were simulated with different initial velocities to obtain statistics on the transition times to vesicles. Shape transformations from flat bilayer discs and bicelles to curved vesicles were monitored by measuring the signed membrane curvature ($\pm H(t)$). Lipid coordinates were fitted to spherical surfaces using least squares optimization to compute membrane curvature along simulations. Curvature away from and toward the upper/cytoplasmic leaflet are reported as positive and negative values, respectively. The statistics of waiting times (t) for the formation of vesicles (bilayer curvature, $|H| \geq 0.15$ nm^{-1}) for different systems were determined from individual replicates. The kinetics of the bilayer-to-vesicle transition was modeled using a Poisson process with a lag time ($t = t' + \tau$). The time t' describes the Poisson process with rate k'.

$$t' = 1/k' \tag{2}$$

The constant lag time τ, captures the time required for vesicle closure from the curved bilayer-disc. The distributions of waiting times are thus,

$$p(t) = k' e^{-k'(t-\tau)} \tag{3}$$

for $t > \tau$. We determined the rate of vesicle formation ($k_{\text{vesicle}} = 1/(t' + \tau)$ for different systems, from fitting the cumulative distribution function for the probability density, $p(t)$, to the observed waiting time distributions estimated from replicates. Maximum likelihood estimates of the vesiculation rate were computed for empty bicelles and bicelles with $KALP_{15}$ peptide, due to low number of vesiculation events.

Curvature sensing: Buckled membrane simulations

A coarse-grained POPC bilayer was used to tile a tessellated buckled surface using *Lipid-Wrapper*[20]. The buckled membrane was solvated with CG water and ions and equilibrated in a periodic box with fixed xy plane (57×28 nm^2) and excess membrane area ($\approx 17\,\text{nm}^2$). This preserved the buckled shape of the bilayer, offering a range of curvature values to be sampled by embedded proteins ($H(x, y) = -0.05 \leq 0 \leq 0.05\,\text{nm}^{-1}$). Proteins (FAM134B-RHD and $KALP_{15}$ in separate simulations) were initially embedded in regions with small local curvature ($H(x, y) \simeq 0$). Following another equilibration phase with proteins, the systems were simulated for $> 20\,\mu$s at 310 K. The membrane profiles of the buckled surface with and without embedded proteins were analyzed by employing the Monge representation to compute local principal curvatures $k_1(x, y)$ and $k_2(x, y)$, the mean curvature $H(x, y)$, and the Gaussian curvature $K_G(x, y)$ (Supplementary Fig. 15; Supplementary Methods; Supplementary Table 6). In preparing the buckled system with FAM134B-RHD, we first computed the projected area difference between the upper and lower leaflets for FAM134B-RHD in flat POPC bilayers in long atomistic (area difference ~ 17.72 nm^2) and coarse-grained (CG) simulations (15.45 nm^2). In the equilibrated buckled membrane, we placed FAM134B-RHD at the correct depth and removed an excess of 30 lipids from the upper leaflet resulting in an area difference of -19.5 nm^2 (using 0.65 nm^2 as area per lipid). Accordingly, the overall area difference is only $\sim$1 % of the total membrane area, and the difference of 19.5 nm^2 to the projected areas of 15.5-17.7 nm^2 is about 0.1-0.2 %. This ensured that there are no significant excess curvature gradients due to the lipid-number asymmetry.

Local membrane curvature calculations

A sinusoidal buckled membrane allowed us to simulate a bilayer with a range of different local curvatures. An initial buckled membrane was built using *Lipid-Wrapper*[20]. A sinusoidal height profile was imposed in the x-direction (length $\approx$ 58 nm; width $\approx$ 28 nm). The local membrane curvature at any given point (x, y) along the membrane was computed using lipid coordinates from each frame of the trajectory. We first approximated the membrane profile of each frame by a height function, $h(x, y)$, using a 2D Fourier expansion,

$$
\begin{aligned}
h(x,y) = &\sum_{m=0}^{k-1}\sum_{n=0}^{k-1}\alpha_{n,m}\cos\left(\frac{2\pi nx}{l_x}\right)\cos\left(\frac{2\pi my}{l_y}\right)\\
&+\sum_{m=0}^{k-1}\sum_{n=0}^{k-1}\beta_{n,m}\cos\left(\frac{2\pi nx}{l_x}\right)\sin\left(\frac{2\pi my}{l_y}\right)\\
&+\sum_{m=0}^{k-1}\sum_{n=0}^{k-1}\gamma_{n,m}\sin\left(\frac{2\pi nx}{l_x}\right)\cos\left(\frac{2\pi my}{l_y}\right)\\
&+\sum_{m=0}^{k-1}\sum_{n=0}^{k-1}\delta_{n,m}\sin\left(\frac{2\pi nx}{l_x}\right)\sin\left(\frac{2\pi my}{l_y}\right)\ ,
\end{aligned}
\tag{4}
$$

where, l_x and l_y impose periodicity of the box vectors (along x and y), $\alpha_{n,m}, \beta_{n,m}, \gamma_{n,m}$, and, $\delta_{n,m}$ are $k \times k$ matrices containing real-valued height coefficients. These Fourier coefficients were optimized by least squares minimization of the sum of squared differences in actual and estimated membrane height,

$$\chi^2 = \sum_{i=1}^{N}(h(x_i, y_i) - z_i)^2 \tag{5}$$

The summation runs over the number N of PO4 beads of the buckled membrane. Using $k = 3$ provided the best approximation of membrane profile without overfitting. The optimized membrane profile $h(x, y)$ was then used to compute the shape operator, $\mathbf{S}(x, y)$, at any given point (x, y) along the membrane buckle.

$$\mathbf{S}(x,y) = \frac{1}{g^{3/2}}\begin{pmatrix}(1+h_y^2)h_{xx} - h_xh_yh_{xy} & (1+h_x^2)h_{xy} - h_xh_yh_{xx}\\(1+h_y^2)h_{xy} - h_xh_yh_{yy} & (1+h_x^2)h_{yy} - h_xh_yh_{xy}\end{pmatrix} \tag{6}$$

where h_x, h_y, h_{xx}, h_{yy}, and h_{xy} are local 1st order and 2nd order partial derivatives of the surface $h(x, y)$ at the given point (x, y), respectively, and $g = (1 + h_x^2 + h_y^2)$.

The determinant of the shape operator $\mathbf{S}(x, y)$ is the Gaussian curvature, $K_G(x, y)$; half its trace is the mean curvature, $H(x, y)$; and its eigenvalues are the principal curvatures, $k_1(x, y)$ and $k_2(x, y)$,

$$\det(\mathbf{S}) = K_G \quad \text{and} \quad \frac{1}{2}\mathrm{tr}(\mathbf{S}) = H \tag{7}$$

$$k_1 = H + \sqrt{H^2 - K_G} \quad \text{and} \quad k_2 = H - \sqrt{H^2 - K_G} \tag{8}$$

The local mean, Gaussian, and principal curvatures were computed over a 2D-grid (0-l_x, and 0-l_y with width 0.1 nm) spanning the buckled membrane for each frame of the trajectory (Supplementary Fig. 15).

Comparing residue covariance and residue contacts of FAM134B-RHD

We determined evolutionary coupling (EC) values of FAM134B-RHD residue pairs using EVfold[12,13]. We first identified homologs containing RHDs using the default search procedure (5 iterations of jackhmmer[21] with e-value cut-off −3) to obtain a good balance between sequence coverage (70% overlap) and total count (n = 1380). After sequence reweighting, the effective number of sequences n_{eff} reduced to 620, resulting in an n_{eff}/L value of 3.4 (where $L = 180$ is the RHD domain size). We obtained EC residue pairs by computing pseudo-likelihood maximization (PLM) scores[12]) for every valid residue pair from the multiple sequence alignment. For comparison, we computed the pair-wise residue contacts between the CG beads of the given residue pair averaged over the FAM134B-RHD CG MD trajectory (every 100 ns for 20 μs). The top ranking EC residue pairs (top 500; see Source Data File) were superimposed onto the structural contact-map for comparison (Supplementary Fig. 20).

FAM134B-RHD cluster formation

Clustering of multiple FAM134B-RHD molecules was simulated under periodic boundary conditions using flat, buckled membrane structures. We generated an initial configuration of the large flat bilayer (62×62 nm^2) with ER-lipid composition embedded with FAM134B-RHDs by duplication and translation (9× along x-y plane) of the coordinates obtained from an earlier simulation of a single RHD (Supplementary Tables 2 and 6). This resulted in RHDs on a square grid 2 nm apart from each other. The new system was then equilibrated followed by production run of 10 μs (Supplementary Fig. 21). To establish curvature mediated sorting and clustering of FAM134B-RHDs, we simulated buckled systems with two distantly placed RHD molecules embedded with both correct and inverted orientations (Supplementary Fig. 22). We monitored RHD-sorting and clustering as a function of sampled local curvatures from both topologies. We then obtained the configuration of a closed tubule structure (length ≈ 97-100 nm; diameter ≈ 12-15 nm) from previous work on stability of membrane nanotubes[22]. Ten FAM134B-RHD molecules were embedded radially along the tubule's length such that the individual proteins were spaced maximally away from each other. RHD containing tubule structure was well equilibrated in explicit solvent ($\sim 3.6 \times 10^6$ beads) using NVT and NPT runs along with position restraints on protein back-bone beads. Production run for 10 μs was carried out, in which we released position restraints to observe curvature-mediated protein sorting and formation of RHD-clusters (Supplementary Fig. 23).

List of primers used in this study

The following primers were used for cloning FAM134B-RHD and its various deletion constructs used for in vitro liposome binding and remodeling assays.

pGEX-6p-1 RHD$_{1\text{-}260}$ QC
5′ -gtgcgcggatccatggcgagcccggcgc-3′
5′ -gcagtcgaattctcatcaatactcgccaataccgaagtcc-3′

pGEX-6p-1 ΔTM12 QC
5′ -gctgagctggaagcgtgatatggttctgagcc-3′
5′ -ggctcagaaccatatcacgcttccagctcagc-3′

pGEX-6p-1 ΔTM34 QC

5′ -gttcaagcaacagagcccggatatcggccaaaaaattt-3′
5′ -aaatttttggccgatatccgggctctgttgcttgaac-3′

pGEX-6p-1 ΔAH$_{L}$ QC
5′ -caaaccggacgaacgtaagcaacagagcccgg-3′
5′ -ccgggctctgttgcttacgttcgtccggtttg-3′
pGEX-6p-1 ΔAH$_{C}$ QC

5′ -ccgctgtttaagtgcaacgattgatgagaattcgactgc-3′
5′ -gcagtcgaattctcatcaatcgttgcacttaaacagcgg-3′

pGEX-6p-1 RHD$_{143\text{-}260}$ QC
5′ -cccctgggatccggcgcgcagctg-3′
5′ -cagctgcgcgccggatcccagggg-3′

The following primers were used for generating various deletion constructs of full-length FAM134B used to study ER-fragmentation in cells. For WT and LIR mutant of FAM134B, previously designed primers were used[15].

FAM134B ΔTM12 (Δ 92-134)
5′ -ctgctgagctggaagagggatatggttttgtctaga-3′
5′ -tctagacaaaaccatatccctcttccagctcagcag-3′

FAM134B ΔTM34 (Δ 194-236)
5′ -gctgtaaatttttgtccaatatcagggctctgctgtttaaaaagaga-3′
5′ -tctctttttaaacagcagagccctgatattggacaaaaatttacagc-3′

FAM134B ΔAH$_{L}$ (Δ 164-188)
5′ -aattccaaaccagatgaaaaacagcagagccctggc-3′
5′ -gccagggctctgctgtttttcatctggtttggaatt-3′

FAM134B ΔAH$_{C}$ (Δ 237-260)
5′ -tctctcacgtttcttctgattaatattacatttaaacaatggacacaaaaatgca-3′
5′ -tgcatttttgtgtccattgtttaaatgtaatattaatcagaagaaacgtgagaga-3′

SUPPLEMENTARY NOTE 1

FAM134B: Sequence annotation and domain organization

Comparison of FAM134B sequence with its homologs revealed a well preserved, well-aligned structured region of the protein (80-260). Profile-based search methods, against both the NCBI conserved domain database (PSSM profile; 85-233; E-value = 4.9e-04), and the Superfamily database (HMM profile; 83-235; E-value = 8.5e-05) identified a conserved reticulon-homology domain (RHD) like sequence (83-235; Supplementary Fig. 1a) in FAM134B. The RHD is a shared evolutionary element present in membrane shaping reticulon proteins (RTN1-4 in humans). RHDs are characterized by the presence of two long hydrophobic regions separated by a ∼60-residue-long linker fragment. Alignments scoring overlap of hydrophobicity profiles illustrate the similarity of the transmembrane (TM) segments of FAM134 and the reticulon family, establishing a likely evolutionary relationship between the two families despite low overall sequence similarity (Supplementary Fig. 1b-c). These characteristics are well captured in the alignment of FAM134B and the Pfam HMM profile for RHD (PF:02453; Supplementary Fig. 1a).

Homologs of FAM134B display a wide range of sequence lengths. In multiple sequence alignments, of FAM134B with its close homologs (Supplementary Fig. 27), the length of the N-terminal disordered region varies. The RHD, including the C-terminal amphipathic helix, and the "LC3 interacting region" (LIR) are well preserved in almost all the homologs, indicating functional importance. The RHD of FAM134B is flanked by two long disordered regions (1-80 and 260-497; red line in Supplementary Fig. 2a). The N-terminal disordered fragment contains a proline-rich sequence (≈1-35) and two short predicted helical regions (Supplementary Fig. 2a). The C-terminal disordered tail contains predicted protein-protein interacting regions (Supplementary Fig. 2a). Among the predicted LIRs, the C-terminal LIR (453-458, DDFELL motif) has been experimentally validated[15]. The sequence stretch adjacent to this LIR, shows strong signals of a coiled-coil-like sequence (colored blue, 458-488 in Supplementary Fig. 2a) with parallel arrangement, thereby suggesting a role in protein dimerization.

Consensus TM assignments revealed an in-out TM topology for each of the two hydrophobic segments (Supplementary Fig. 2). The presence of polar residues in between the two hydrophobic stretches of each segment implies a hairpin-like arrangement (TM12 and TM34; Supplementary Fig. 2b). Profiles of the free energy of membrane insertion are consistent with this assignment of the TM helices (black curve in Supplementary Fig. 2b)[10]. The data in Supplementary Fig. 1 also point to significant membrane affinities of the flanking amino acids. In particular, there are two well conserved segments which are predicted as amphipathic helices that flank TM34 on either side (Supplementary Fig. 2b). Overall, the RHD (80-260) is predicted to contain structured elements that associate tightly with the endoplasmic reticulum (ER) membrane (Supplementary Fig. 2c). By integrating secondary structure prediction, disorder prediction, TM topology prediction, and conservation information, we divided the FAM134B-RHD into five distinct parts and modeled their 3D structure independently (see Methods).

SUPPLEMENTARY NOTE 2

Transmembrane segments of FAM134B fold into TM hairpin structures

The transmembrane fragments TM12 and TM34 were modeled using TM regions of membrane protein and lipid binding regions of globular proteins as templates, respectively (Table 1). Consensus TM prediction methods assigned an in-out topology for each of these regions (Supplementary Fig. 2a). The length of each TM fragment (≈ 40 residues) is too long for a typical

single pass TM helix (22-24 residues) and too short for the two canonical double pass TM helices with additional loop residues. Each TM segment comprises two predicted helical segments connected by a short polar loop (three to four residues). Independent sequence-based TM-kink prediction[23] also identified these short polar residue stretches as turn promoting regions. Therefore, the most plausible structure for the TM fragments constitutes a TM hairpin formed by two short hydrophobic helices (four to five turns each) with a polar hairpin turn connecting them. Given the thickness of the ER membrane (3.75 ± 0.04 nm), this organization results in the least hydrophobic mismatch[24]. This membrane topology is consistent with those of closely related reticulon and reticulon-like proteins, DP1/YOP/REEP[25–27].

In the absence of homologs of the RHD with known structure and reliable remote relationships within structure databases, we used context specific alignments of individual fragments, maximizing the overlap of secondary structure patterns and hydrophobicity patterns, for e.g. helix-tight-turn-helix motifs with overlapping hydrophobicity for both TM12 and TM34 fragments. We were able to obtain initial rough model of the hairpin structures for TM12 fragment using a fragment within the Respiratory complex I protein from *E. coli*, a membrane protein. For the TM34 fragment we used a combination of Glutaryl-CoA DHase and Butaryl-CoA DHase proteins. These proteins oxidize short fatty acyl-CoA and therefore, interact with hydrophobic fatty acyl chains. Despite choosing a non-membrane protein as the initial template, the aligned template-TM34 fragments have matching secondary-structure patterns and reflect the hydrophobicity character required to interact with acyl chains. Furthermore, any inaccuracies in the initial template-target alignments and bias of the initial models are refined using molecular dynamics simulations with physics-based potentials in the presence of lipid membrane.

To identify correct tertiary contacts across the TM helices of the hairpin structure, we performed extensive molecular dynamics (MD) simulations of the initial fragment structures. We first performed long coarse-grained MD simulations in POPC bilayers (Supplementary Fig. 3). The TM fragment models quickly rearranged into a favorable position within the bilayer, at depths of $d_{\mathrm{TM12}} \approx 1.5$ nm and $d_{\mathrm{TM34}} \approx 2.0$ nm, and assumed characteristic equilibrium tilt angles of θ_{TM12} and $\theta_{\mathrm{TM34}} \approx$ 10-12° with respect to the bilayer normal (Supplementary Fig. 3). This organization remained unchanged over the entire coarse-grained (CG) simulation time (100 μs), indicating that the fragment structures had relaxed to an equilibrium depth and orientation with respect to the bilayer. To recover the appropriate helix-helix interfaces for each of the TM hairpins, we sampled 10000 conformations from each of the CG trajectories at equal intervals and clustered them to obtain the most populated conformations for each TM fragment (see Supplementary Methods). We then back-mapped representative CG configurations from the most populated clusters to all-atom representations and performed all-atom MD simulations to assess the helix-helix interfaces in more detail.

FAM134B:TM12

Clustering of conformations sampled in the CG TM12-fragment trajectory resulted in a total of 5 clusters with a single large cluster (99.48 %; Supplementary Fig. 3a-c). This indicated that TM12 model is relatively stable with $\approx$ 160 contacts stabilizing the helix-helix interface (Supplementary Fig. 3b). The two largest clusters have a common helix-helix interface, but vary in their specific residue contacts (Supplementary Fig. 3d-4e). A back-mapped TM12-cluster-1 representative provided us with a starting structure to refine the all-atom model for the hairpin (Supplementary Fig. 4). In all-atom MD simulations this structure remained stable for $>$2.5 μs (Supplementary Fig. 4a-b). Helices TM1 and TM2 are appropriately positioned between the bilayer phosphate groups (Supplementary Fig. 4c), whereas the loop residues are closer to the luminal/lower leaflet of the bilayer. The polar residues at helix termini and the polar loop help anchor the hairpin structure to both the cytosolic leaflet and the luminal leaflet, respectively

(Supplementary Fig. 4d). Each TM helix is slightly tilted with respect to the bilayer normal ($\theta_{\mathrm{TM1}} = 20°$ and $\theta_{\mathrm{TM2}} = 20°$). The two helices cross at an angle of 52°. Helix-helix contacts are concentrated close to the crossing regions (TM1: N103, F108; TM2: V122, L125) stabilizing the hairpin (Supplementary Fig. 4e-f).

FAM134B:TM34

A total of 15 clusters were obtained for the TM34 fragment from the CG TM34-trajectory (Supplementary Fig. 3f-h). Clusters 1, 2, and 3 account for 50.7, 29.7, and 9.9 % of the population, respectively (Supplementary Fig. 3g). The TM34-cluster-1 helix-helix interface is dominated by non-polar contacts, whereas clusters 2 and 3 contain both polar and non-polar groups at their helix-helix interfaces (Supplementary Fig. 3i-k). TM34-cluster-3 structure showed more extensive contacts among the same set of interface residues as cluster 2 (Supplementary Fig. 3k). We back-mapped the top three cluster representatives of the TM34 fragment and performed all-atom MD simulations ($> 3\,\mu$s each). In all three simulations, the glycine-rich loop (G211-G216) underwent conformational changes. In simulations started from representatives of cluster 1 and 2, the inter-helix interactions reorganized. The two helices transiently disassociated, re-associating on a longer time scale ($> 10\,\mu$s; not shown). By contrast, in TM34-cluster-3 the helix-helix contacts remained stable (Supplementary Fig. 5a-b) despite relaxation of the glycine-rich loop (Supplementary Fig. 5b). The structure of the relaxed TM34-cluster-3 resembles both the initial TM34-fragment model (used for conformational sampling) and the structure in the MD simulation starting from TM34-cluster-1 after helix dissociation and re-association, with a root-mean-square distance (RMSD) of the C_α atoms of less than 3.0 Å). Together, these results point to the TM34-cluster-3 conformation as native-like and stable. Accordingly, the TM34-cluster-3 was used for further analysis (Supplementary Fig. 5a). Two lysine residues flanking the TM34 fragment (K195 and K234) anchor the termini of the helical hairpin at the cytosolic leaflet (Supplementary Fig. 5c). The polar loop residues (S212, Y213, and P215) form contacts at the water-bilayer interface of the luminal leaflet (Supplementary Fig. 5d). The TM34 hairpin is stabilized by polar interactions ($>$ 10 heavy atom contacts) between TM3 (C197, C201, T205, and T208) and TM4 (Y221, F228), as illustrated in Supplementary Figs. 5e-f.

SUPPLEMENTARY NOTE 3

Structure and organization of linker fragment

The linker-fragment (129-196) connects the two TM hairpin structures on the cytosolic side of the ER membrane. Upon detailed comparison of aligned linker sequences from FAM134B/RETR1 and Nogo/RTN4 (Supplementary Figure 1c), we found that 54/60 residues were non-identical. There are two conformations of the Nogo-66 protein: (1) A solution conformation derived from solution NMR in aqueous solution[28] and (2) a "membrane-adhered" conformation resolved using NMR in the presence of DPC detergent micelles[29]. In solution, the Nogo-66 fragment is functional and adopts an extended structure with a central long helix, consistent with secondary structure predictions[28]. However, in DPC micelles, the Nogo-66 adopts a more compact shape with bent helices (at -IESS- and -ALGHV-)[29]. Comparison of helix stretches to FAM134B sequence, showed amino-acid differences in this region (-PDER- and -AESWM- aligned to RTN4/Nogo; Supplementary Fig. 1c). Moreover, in FAM134B, this helical segment also has features characteristic of an amphipathic helix (169-186, $\langle H \rangle = 0.76$ and $\langle \mu_H \rangle = 0.35$; Supplementary Fig. 6a) and could in principle interact strongly with the membrane. Although this amphipathic nature is preserved in the entire RHD-family including RTN4/Nogo (Supplementary Figs. 6a),

the FAM134B-sequences have a slightly higher mean hydrophobicity than the RTN4 sequences (due to more bulky aromatic residues in AH_L), which indicates stronger membrane binding (Supplementary Fig. 6b). Furthermore, in DPC adhered structures of Nogo-66, this helix is oriented with its hydrophobic face towards the protein interior on the opposite side of the presumed membrane interaction site[29]. Due to the variations in solution and DPC micelle-bound structures of Nogo-66, its functional diversity; Nogo-66 acts as signalling molecule, inhibiting axonal growth by binding to Nogo receptor[30], and observed sequence differences (54/60 residues are non identical; Supplementary Fig. 1b), we modeled the FAM134B-linker fragment independently with a single rigid helix and assessed membrane binding using MD simulations. We refer to this amphipathic-helix region as AH_L in our linker-fragment structure.

We assessed the linker fragment model by performing all-atom MD simulations both in aqueous solution (Supplementary Fig. 6b-c) and in the presence of POPC bilayers (Supplementary Fig. 6d-h). In free solution, the helical structure of AH_L persisted, displaying moderate kinking motions near N177 (Supplementary Fig. 6c-d and S4). The rest of the peptide adopted diverse coil-like conformations (Supplementary Fig. 6b-c). An additional short helix (≈ 1 turn corresponding to 146-QLWRSL-151) persisted in solution.

AH_L is immediately adjacent to the TM fragment TM34 (196-234), which would place the helix at the membrane-water interface on the cytosolic side. To test if the predicted AH_L has membrane binding ability, we performed coarse-grained MD simulations (Supplementary Fig. 6d). We observed docking of the linker-fragment to the membrane surface, mediated by the predicted hydrophobic face of the AH_L (Supplementary Fig. 6d). After several docking attempts, AH_L successfully inserted into the membrane interface. Docking was initiated by a steep approach of AH_L to the bilayer, promoting AH_L-membrane contacts, and subsequent sliding of the AH_L onto the membrane surface (Supplementary Fig. 6d-e). These simulations support the membrane binding ability of AH_L.

In an all-atom MD simulations in the presence of a POPC bilayer, starting from a pre-oriented linker-fragment placed at a distance of 3 nm from the bilayer, the AH_L (164-184) spontaneously embedded into the membrane surface (Supplementary Fig. 6f). The hydrophobic face of AH_L (I171, W175, F178, L182, and M185) formed extensive hydrophobic interactions with the acyl chains of lipids. The polar and charged face (H169, E173, N177, Q183, and E184) of the helix interacted with lipid head groups and water (Supplementary Fig. 6g). The AH_L docked on the membrane was shortened (169-186) by ≈ 2 helical turns in comparison to the solution structure (Supplementary Fig. 6h). The corresponding residues adopted coil-like extended states on the membrane. The N-terminal overhang of the linker fragment (corresponding to the hydrophobic residues of TM2) adopted helical conformations with membrane contacts.

These results taken together are indicative of a strong helical structure within the linker fragment with membrane binding ability. Therefore, the AH_L behaves as a potential amphipathic helix that provides additional membrane interactions.

SUPPLEMENTARY NOTE 4

Structure and organization of C-terminal amphipathic helix

C-terminal to TM34 (residues 196-234), the RHD contains a second predicted amphipathic helix, AH_C, formed by residues 237 to 254 with hydrophobicity[6] $\langle H \rangle = 0.382$ and hydrophobic moment $\langle \mu_H \rangle = 0.481$ (Supplementary Fig. 7a). Despite being excluded from the RHD profile (83 to 235), this region is well conserved (Supplementary Fig. 27). Comparison of this stretch in FAM134 and RTN sequences shows similar hydrophobicity and hydrophobic moments[6] consistent with an amphipathic helix (Supplementary Fig. 7a). The slightly larger $\langle H \rangle$ and $\langle \mu_H \rangle$ values for FAM134B homologs compared to other RHDs indicate an even stronger amphipathic character.

In free-solution all-atom MD simulations, the AH_C unfolded quickly (within the first 50 ns) and assumed a predominantly coil-like extended conformation (Supplementary Fig. 7b-c). Using CG-MD simulations, we observed docking of the prefolded AH_C structure onto the bilayer (Supplementary Fig. 7c-d). The docked configuration resembled the orientation corresponding to maximal hydrophobic moment, with the hydrophobic face embedded in the cytosolic leaflet (Supplementary Fig. 7d). To confirm this peripheral membrane binding mode of the AH_C, we performed all-atom MD simulations in the presence of lipid bilayer, initiated from a pre-oriented helix configuration. In these simulations, the helical structure remained stably embedded in the cytosolic leaflet (Supplementary Fig. 7e-f), showing strong interactions between the predicted non-polar, aliphatic face (I238, I242, I246, V249, and, L253) and the lipid acyl chains (Supplementary Fig. 7f). The water-facing side of the AH_C was predominantly charged (D237, Q240, K241, S244, K245, K247, S248, K252, and, D254), fixing the orientation of the helix at the bilayer-water interface and confirming its amphipathic nature (Supplementary Fig. 7f). The AH_C unfolded in solution (Supplementary Fig. 7g), and remained helical on the POPC bilayer (Supplementary Fig. 7h). This feature is typical of amphipathic helices (e.g., YOP1P-AmpH), where the helical conformation is stabilized only in the presence of lipids micelles or vesicles[27].

SUPPLEMENTARY NOTE 5

Assembly of FAM134B-RHD

Equilibrium positions and orientations of the all the modelled FAM134B-RHD fragments (TM12, linker, TM34, C-ter) were obtained from all-atom MD simulations in the presence of POPC bilayers. In MD simulations, the short (11 residues) N-terminal fragment adopted predominantly extended coil structures and was thus treated as an overhang of TM12. By aligning common overhang residues, we joined fragments adjacent in sequence, ensuring that their membrane orientation was maintained. The oriented fragment models were then stitched into a single consecutive all-atom model using Modeller[31] after remodeling overhang residues connecting individual fragments using its loop modelling protocol. The resulting FAM134B-RHD model was energy minimized and equilibrated in the presence of POPC bilayers using CG simulations. The intact CG RHD structure was then back-mapped to all-atom representation and subjected to long all-atom MD simulations for refinement. We also used this simulation to assess local membrane properties (Supplementary Fig. 8). Additional long CG simulations ($> 20\ \mu$s) were performed with POPC and model ER membranes.

Sequence covariance analysis.

The structure and dynamics of the FAM134B-RHD model is supported by sequence covariance analysis. In Supplementary Fig. 20, we compare the contact map obtained from our MD simulations with predicted coevolving residue pairs (see Supplementary Methods). Despite the relatively small number of sequences and the resulting noise in the EC estimates, we found that at least 25% of top-ranking ECs (top 500) overlap with the observed tertiary contacts from the dynamic RHD structure. In particular, ECs between TM1-TM2 and TM3-TM4, and between TM12-TM34 hairpins are consistent with the calculated contact map (red, blue, and green circles in Supplementary Fig. 20). The good consistency of EC values and contact maps, despite the inherent noise in the EC calculation resulting from the small number of sequences, further supports our dynamic RHD model.

Structural studies on RHDs.

The relatively loose interaction between TM12 and TM34 hairpins is also supported by structural studies. Structural studies on YOP1 provide a partial picture on the packing of RHD transmembrane hairpins. YOP1 is the only RHD-like protein whose structure has been studied in some detail (though not fully resolved because of the inherent dynamics). NMR experiments on YOP1P in micelles by Schnell and coworkers[27] could not resolve NOEs across the two TM-hairpins, thereby supporting a model in which the two TM regions are only loosely interacting. Furthermore, ^{15}N R1 and R2 relaxation measurements showed that the linker connecting the two TM segments is highly dynamic (over nanoseconds time scales), indicating that the distance distributions between TM12 and TM34 hairpins could be broad. The measurements also showed that the luminal hydrophilic loops are more rigid, comparable to the TM helical residues, indicating that the individual hairpin structures are stable but their relative orientations might not be restrained to a fixed structure. It is noteworthy that the linker between TM12 and TM34 in FAM134B is considerably longer than in YOP1, making stable TM12:TM34 interactions in FAM134B even less likely. Moreover, the amphipathic linker helix AH_L of FAM134B shared with reticulons and other RHDs but, likely, not with YOP1, provides an additional spacer between the cytosolic ends of TM12 and TM34. RHDs and RHD-like proteins are grouped under two different Pfam families, proteins containing canonical RHDs (PF02453) and proteins with RHD-like TB2/DP1/HVA22 domain structure (PF03134). The main difference between these profiles reveals the presence of a ~60-residue-long linker in PF02453 and a shorter linker in PF03134. Moreover, the conservation status for TM12 and TM34 is also different. TM34 is well preserved and more conserved in comparison to TM12 (TM1 is absent in related REEPs 1-4). These data indicate that interactions between TM12 and TM34 of RHDs and RHD-like proteins if any, are at least not extensive.

SUPPLEMENTARY NOTE 6

FAM134B-RHD induces curvature in open bilayer patches

In MD simulations of open bilayer patches with and without embedded protein, we monitored bilayer curvature changes, $\pm H(t)$ (Supplementary Fig. 9; positive and negative values refer to curving away from or towards the cytosolic side of the RHD). FAM134B-RHD embedded in discontinuous bilayer discs induces membrane curvature and enhances the rates of vesicle formation (Supplementary Fig. 9a). In repeated simulations (93 runs) we found that the RHD induces strong positive curvature ($H \approx +0.15$ nm^{-1}) of the bilayer disc, resulting in swift bilayer-to-vesicle transitions ($<$ 100 ns; Supplementary Fig. 9b; Supplementary movie 1). In control simulations, bilayer discs containing the $KALP_{15}$ peptide, curvature changes are stochastic and vesiculation events are slower (up to 200 ns; inset; Fig. 3b). In contrast to the RHD, both positive ($n_+ = 40$) and negative curvature ($n_- = 38$) changes in bilayer disc are observed with nearly equal probability (78 runs; inset; Supplementary Fig. 9b). In additional simulations using membrane patches with ER-lipid composition (Supplementary Fig. 10a), we tested the effect of RHD-mediated in silico curvature induction on ER-membranes. We found that the bilayer patches mimicking ER-lipid composition were less stiff in comparison to pure POPC bilayer patches and in the presence of embedded FAM134B-RHD formed closed vesicles even more swiftly (Supplementary Fig. 10b). In 90 % of the cases (95 runs), we observed vesicle formation within the first 70 ns of the simulation, demonstrating that the RHDs can induce curvature faster in more fluid bilayers.

SUPPLEMENTARY NOTE 7

Molecular signatures determining curvature induction by FAM134B-RHD

To find out signature structural motifs/fragments responsible for maximal curvature induction, we performed ensemble simulations using both bilayer-to-vesicle and bicelle-to-vesicle simulations with embedded FAM134B-RHD fragments (Supplementary Figs. 12-14). Interestingly, individual TM fragments (i.e. only TM12 and only TM34) display only moderate effects on both bilayer-to-vesicle transitions and bicelle-to-vesicle-transitions. TM fragments orient in the bilayer such that their hairpin tips are anchored into the surface of the luminal leaflet and the termini face the cytoplasmic leaflet, respectively (Fig. 2a-b). A wedging motion of the two helices within a single TM hairpin embedded in bilayer discs can in principle induce strong preferential curvature, away from the cytoplasmic leaflet. However, we found that the presence of single short TM hairpin fragments in bilayers has only a moderate effect on the rate of vesicle formation in bilayer-discs (acceleration by factors of 1.36 for TM12 and 1.26 for TM34; Supplementary Fig. 13b-d). The number of vesiculation events along the cytoplasmic leaflet and luminal leaflets is marginally higher for TM12 ($n_+ > n_-$; Supplementary Fig. 12b), and comparable for TM34 ($n_+ \simeq n_-$; Supplementary Fig. 12c). In more challenging bicelles-to-vesicle simulations, we observed even fewer total vesiculation events for individual TM12 and TM34 fragments (24/100 and 37/100; Fig. 13a-b) with no clear directional preference.

By contrast, the linker and C-terminal fragments show strong and specific bilayer and bicelle curvature induction (with $n_+ \gg n_-$; Fig. 5d-e). Both fragments harbor amphipathic helices (AH_L and AH_C, respectively) that engage in strong peripheral membrane binding. We found that the linker fragment including the amphipathic helix induced bilayer curvature much faster than any other fragment of the RHD (factor 1.71; Supplementary Fig. 12d-f). This acceleration is comparable to the intact RHD (acceleration factor 2.25), indicating its relative importance in curvature generation. The linker fragment occupies a substantial fraction of the cytoplasmic leaflet, creating a large asymmetric footprint of the RHD. Moreover, hydrophobic interactions of the AH_L with the lipid tails enable high positive curvature induction, inducing fast vesicle formation (Fig. 5f; Supplementary Table 5). In bicelle-to-vesicle simulations, the AH_L and AH_C fragments induced strong directional curvatures (with $n_+ \gg n_-$; Supplementary Fig. 13c-d) resulting in more frequent and directional vesiculation events (92 and 42 respectively). These studies reveal that the single TM hairpins alone cannot induce directional and quick curvature in bilayers and bicelles. Presence of both TM fragments, separated by symmetry-breaking amphipathic helices (AH_L and AH_C) is essential at least for in silico curvature induction.

SUPPLEMENTARY NOTE S8

AH segments induce anisotropic curvature which enable clustering of RHD structural elements

FAM134B-RHD adopted a preferred orientation relative to the local curvature field. The amphipathic helices of FAM134B induce maximal membrane curvature and therefore tend to orient with respect to the local curvature field (Supplementary Fig. 17). AH_L flanked by the two TM hairpin segments is constrained and adopts a diagonal orientation with respect to the local principal curvature field ($\psi \approx 135°/315°$; Supplementary Fig. 17a). AH_C prefers highly curved surfaces and orients perpendicular to the local principal curvature axis ($\psi \approx 90°/270°$; Supplementary Fig. 17b). These results indicate that the FAM134B-RHD orients to match the deformations induced by its membrane-adhered amphipathic helices to the curvature field of the membrane buckle. The orientation anisotropy observed is indicative of an underlying anisotropic

curvature induction behavior.

To explore the curvature preference and assembly pathway of the FAM134B structural elements, we performed a simulation of a buckled membrane containing the FAM134B-RHD fragments TM12, TM34, AH_L, and AH_C (Supplementary Fig. 18). We found that the AH_L and AH_C fragments quickly moved to regions of the buckle with high positive curvature (within 500 ns). The hairpin fragment TM34, first, diffused to the positively curved region of the buckle and clustered with the AH_L fragment (at 3387 ns; Supplementary Fig. 18b). Then the hairpin fragment TM12 merged with the cluster (at 10240 ns; Supplementary Fig. 18b). The terminal short AH_C fragment transiently attached to the AH_L:TM34:TM12 cluster (Supplementary Fig. 18b). The AH_L fragment appears to form the nucleus for clustering of the other fragments at the highly curved region of the buckle.

SUPPLEMENTARY REFERENCES

[1] Stamm, M., Staritzbichler, R., Khafizov, K. & Forrest, L. R. AlignMe–a membrane protein sequence alignment web server. *Nucleic Acids Res.* **42**, W246–W251 (2014).

[2] Thompson, J. D., Gibson, T. J., Plewniak, F., Jeanmougin, F. & Higgins, D. G. The CLUSTAL_X windows interface: flexible strategies for multiple sequence alignment aided by quality analysis tools. *Nucleic Acids Res.* **25**, 4876–4882 (1997).

[3] Waterhouse, A. M., Procter, J. B., Martin, D. M., Clamp, M. & Barton, G. J. Jalview Version 2-A multiple sequence alignment editor and analysis workbench. *Bioinformatics* **25**, 1189–1191 (2009).

[4] Jones, D. D. T. D. Protein secondary structure prediction based on position-specific scoring matrices. *J. Mol. Biol.* **292**, 195–202 (1999).

[5] Tsirigos, K. D., Peters, C., Shu, N., Käll, L. & Elofsson, A. The TOPCONS web server for consensus prediction of membrane protein topology and signal peptides. *Nucleic Acids Res.* **43**, W401–W407 (2015).

[6] Gautier, R., Douguet, D., Antonny, B. & Drin, G. HELIQUEST: A web server to screen sequences with specific α-helical properties. *Bioinformatics* **24**, 2101–2102 (2008).

[7] Kozlowski, L. P. & Bujnicki, J. M. MetaDisorder: a meta-server for the prediction of intrinsic disorder in proteins. *BMC Bioinformatics* **13** (2012).

[8] Kalvari, I., Tsompanis, S., Mulakkal, N. C., Osgood, R., Johansen, T., Nezis, I. P. & Promponas, V. J. iLIR: A web resource for prediction of Atg8-family interacting proteins (2014).

[9] Delorenzi, M. & Speed, T. An HMM model for coiled-coil domains and a comparison with PSSM-based predictions. *Bioinformatics* **18**, 617–625 (2002).

[10] Hessa, T., Meindl-Beinker, N. M., Bernsel, A., Kim, H., Sato, Y., Lerch-Bader, M., Nilsson, I., White, S. H. & Von Heijne, G. Molecular code for transmembrane-helix recognition by the Sec61 translocon. *Nature* **450**, 1026–1030 (2007).

[11] Gapsys, V., De Groot, B. L. & Briones, R. Computational analysis of local membrane properties. *J. Comput. Aided. Mol. Des.* **27**, 845–858 (2013).

[12] Marks, D. S., Colwell, L. J., Sheridan, R., Hopf, T. A., Pagnani, A., Zecchina, R. & Sander, C. Protein 3D structure computed from evolutionary sequence variation. *PLoS One* **6**, 28766 (2011).

[13] Hopf, T. A., Colwell, L. J., Sheridan, R., Rost, B., Sander, C. & Marks, D. S. Three-dimensional structures of membrane proteins from genomic sequencing. *Cell* **149**, 1607–1621 (2012).

[14] Schneider, C. A., Rasband, W. S. & Eliceiri, K. W. NIH Image to ImageJ: 25 years of image analysis. *Nat. Methods* **9**, 671–5 (2012).

[15] Khaminets, A., Heinrich, T., Mari, M., Grumati, P., Huebner, A. K., Akutsu, M., Liebmann, L., Stolz, A., Nietzsche, S., Koch, N., Mauthe, M., Katona, I., Qualmann, B., Weis, J., Reggiori, F., Kurth, I., Hübner, C. A. & Dikic, I. Regulation of endoplasmic reticulum turnover by selective autophagy. *Nature* **522**, 354–358 (2015).

[16] Källberg, M., Wang, H., Wang, S., Peng, J., Wang, Z., Lu, H. & Xu, J. Template-based protein structure modeling using the RaptorX web server. *Nat. Protoc.* **7**, 1511–1522 (2012).

[17] Daura, X., Gademann, K., Jaun, B., Seebach, D., van Gunsteren, W. F. & Mark, A. E. Peptide Folding: When Simulation Meets Experiment. *Angew. Chemie Int. Ed.* **38**, 236–240 (1999).

[18] Wassenaar, T. A., Pluhackova, K., Böckmann, R. A., Marrink, S. J. & Tieleman, D. P. Going backward: A flexible geometric approach to reverse transformation from coarse grained to atomistic models. *J. Chem. Theory Comput.* **10**, 676–690 (2014).

[19] Parrinello, M. & Rahman, A. Polymorphic transitions in single crystals: A new molecular dynamics method. *J. Appl. Phys.* **52**, 7182–7190 (1981).

[20]Durrant, J. D. & Amaro, R. E. LipidWrapper: An Algorithm for Generating Large-Scale Membrane Models of Arbitrary Geometry. *PLoS Comput. Biol.* **10** (2014).

[21]Potter, S. C., Luciani, A., Eddy, S. R., Park, Y., Lopez, R. & Finn, R. D. HMMER web server: 2018 update. *Nucleic Acids Res.* **46**, W200–W204 (2018).

[22]Bahrami, A. H. & Hummer, G. Formation and Stability of Lipid Membrane Nanotubes. *ACS Nano* **11**, 9558–9565 (2017).

[23]Meruelo, A. D., Samish, I. & Bowie, J. U. TMKink: A method to predict transmembrane helix kinks. *Protein Sci.* **20**, 1256–1264 (2011).

[24]Mitra, K., Ubarretxena-Belandia, I., Taguchi, T., Warren, G. & Engelman, D. M. Modulation of the bilayer thickness of exocytic pathway membranes by membrane proteins rather than cholesterol. *Proc. Natl. Acad. Sci.* **101**, 4083–4088 (2004).

[25]Voeltz, G. K., Prinz, W. a., Shibata, Y., Rist, J. M. & Rapoport, T. A. A class of membrane proteins shaping the tubular endoplasmic reticulum. *Cell* **124**, 573–586 (2006).

[26]Hu, J., Shibata, Y., Voss, C., Shemesh, T., Li, Z., Coughlin, M., Kozlov, M. M., Rapoport, T. A. & Prinz, W. A. Membrane proteins of the endoplasmic reticulum induce high-curvature tubules. *Science (80-.).* **319**, 1247–1250 (2008).

[27]Brady, J. P., Claridge, J. K., Smith, P. G. & Schnell, J. R. A conserved amphipathic helix is required for membrane tubule formation by Yop1p. *Proc. Natl. Acad. Sci.* **2014**, 201415882 (2015).

[28]Li, M., Liu, J. & Song, J. Nogo goes in the pure water: solution structure of Nogo-60 and design of the structured and buffer-soluble Nogo-54 for enhancing CNS regeneration. *Protein Sci.* **15**, 1835–1841 (2006).

[29]Vasudevan, S. V., Schulz, J., Zhou, C. & Cocco, M. J. Protein folding at the membrane interface, the structure of Nogo-66 requires interactions with a phosphocholine surface. *Proc. Natl. Acad. Sci. U. S. A.* **107**, 6847–6851 (2010).

[30]GrandPré, T., Nakamura, F., Vartanlan, T. & Strittmatter, S. M. Identification of the Nogo inhibitor of axon regeneration as a Reticulon protein. *Nature* **403**, 439–444 (2000).

[31]Šali, A. & Blundell, T. L. Comparative protein modelling by satisfaction of spatial restraints. *J. Mol. Biol.* **234**, 779–815 (1993).
