# Supplementary figures and images for: Curvature induction and membrane remodeling by FAM134B reticulon homology domain assist selective ER-phagy
Source: Nat Commun. 2019 May 30;10:2370. doi: 10.1038/s41467-019-10345-3 (PMC6542808; doi:10.1038/s41467-019-10345-3)

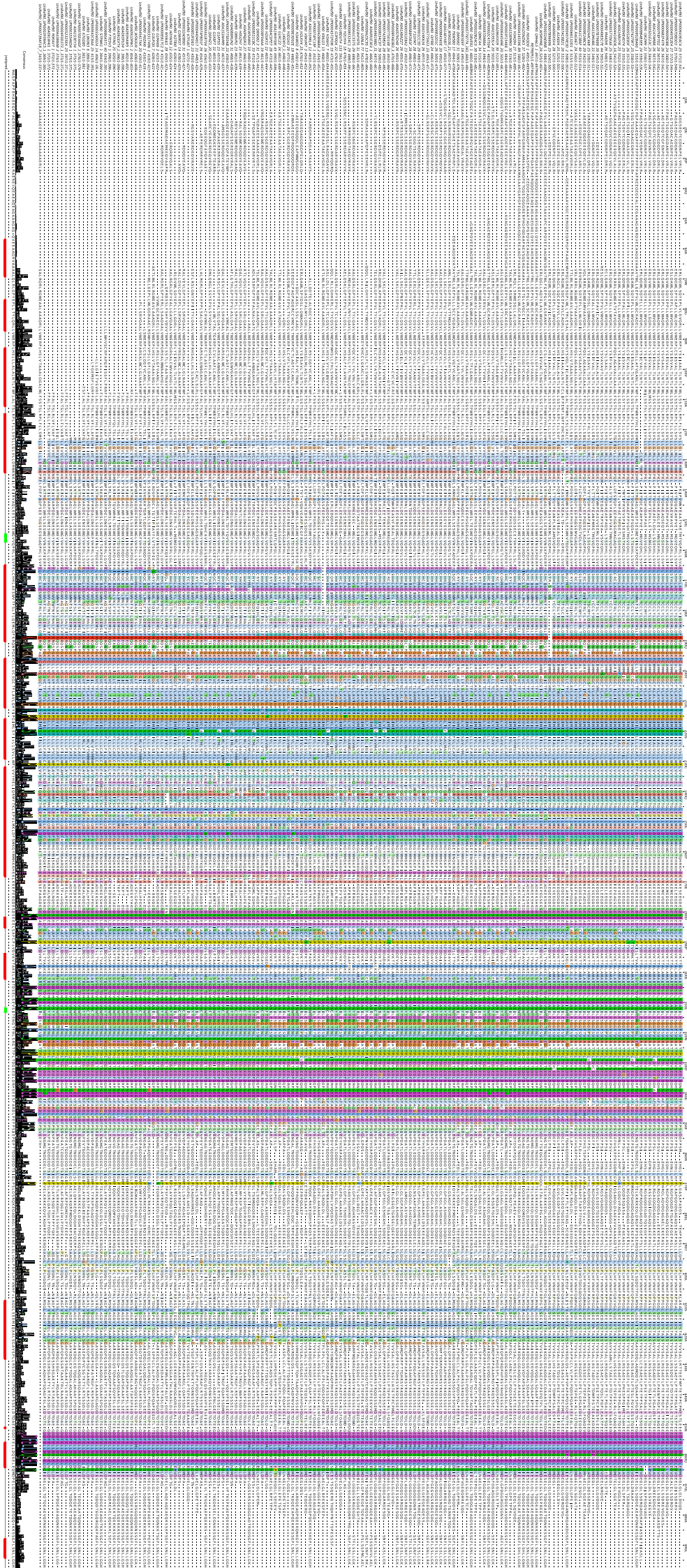

Chromosome: 100.0 Mb

Supplement: Supplementary file 8 — Supplementary Data 1 [file 41467_2019_10345_MOESM8_ESM.pdf]
