# Supplementary material for: Curvature induction and membrane remodeling by FAM134B reticulon homology domain assist selective ER-phagy
Source: Nat Commun. 2019 May 30;10:2370. doi: 10.1038/s41467-019-10345-3 (PMC6542808; doi:10.1038/s41467-019-10345-3)
Supplement: Supplementary file 9 — Description of Additional Supplementary Files [file 41467_2019_10345_MOESM9_ESM.docx]

**Title:** Supplementary Movie 1:
**Description:** Movie showing curvature induction and vesiculation of pure POPC bilayers disc by FAM134B-RHD (green; representative simulation; 500 ns). The cross section along x-z plane provides the detailed picture of the vesicle closure.

**Title:** Supplementary Movie 2:
**Description:**  Movie showing MD simulation of empty bicelles made from DMPC (grey) and DHPC (red) lipids (Representative control simulation; 1000 ns).

**Title:** Supplementary Movie 3:
**Description:**  Movie showing MD simulation of vesicle formation from bicelles induced by FAM134B-RHD (green; representative simulation; 1000 ns).

**Title:** Supplementary Movie 4:
**Description:**  Movie showing the top and side views of the simulation trajectory of a buckled membrane in the presence of FAM134B-RHD (green; 5000 ns).

**Title:** Supplementary Movie 5:
**Description:**  Movie showing the top and side views of the MD trajectory of a buckled membrane in the presence of KALP15 peptide (blue; control simulation; 5000 ns).

**Title:** Supplementary Movie 6:
**Description:**  Movie showing the starting configuration of the giant tubule (PO4 beads, orange) with 10 FAM134B-RHDs molecules (colored by chain) along with simulation trajectory (3.6 million particles; ~7000 ns) showing the formation and three inverted-pyramid-shaped RHD clusters which amplify local tubule curvature.

**Title:** Supplementary Data file:
**Description:**  Detailed MAFFT alignment file showing the multiple sequence alignment of the full-length FAM134B (RETR1). Schematic with summary shown in Supplementary Fig. 27.
